# Comprehensive molecular dissection of TIFY Transcription factors reveal their dynamic responses to biotic and abiotic stress in wheat (*Triticum aestivum* L*.*)

**DOI:** 10.1038/s41598-021-87722-w

**Published:** 2021-05-06

**Authors:** Poonam Singh, Kunal Mukhopadhyay

**Affiliations:** Department of Bio-Engineering, Birla Institute of Technology, Mesra, Ranchi, 835215 Jharkhand India

**Keywords:** Functional genomics, Genomics, Plant biotechnology

## Abstract

The plant specific TIFY (previously known as ZIM) transcription factor (TF) family plays crucial roles in cross talk between Jasmonic Acid and other phytohormones like gibberellins, salicylic acid, abscisic acid, auxin, and ethylene signaling pathways. Wheat yield is severely affected by rust diseases and many abiotic stresses, where different phytohormone signaling pathways are involved. *TIFYs* have been studied in many plants yet reports describing their molecular structure and function in wheat are lacking. In the present study, we have identified 23 novel *TIFY* genes in wheat genome using in silico approaches. The identified proteins were characterized based on their conserved domains and phylogenetically classified into nine subfamilies. Chromosomal localization of the identified *TIFY* genes showed arbitrary distribution. Forty *cis*-acting elements including phytohormone, stress and light receptive elements were detected in the upstream regions of TIFY genes. Seventeen wheat microRNAs targeted the identified wheat *TIFY* genes. Gene ontological studies revealed their major contribution in defense response and phytohormone signaling. Secondary structure of TIFY proteins displayed the characteristic alpha–alpha–beta fold. Synteny analyses indicated all wheat *TIFY* genes had orthologous sequences in sorghum, rice, maize, barley and *Brachypodium* indicating presence of similar TIFY domains in monocot plants. Six *TIFY* genes had been cloned from wheat genomic and cDNA. Sequence characterization revealed similar characteristics as the in silico identified novel *TIFY* genes. Tertiary structures predicted the active sites in these proteins to play critical roles in DNA binding. Expression profiling of *TIFY* genes showed their contribution during incompatible and compatible leaf rust infestation. *TIFY* genes were also highly expressed during the initial hours of phytohormone induced stress. This study furnishes fundamental information on characterization and putative functions of *TIFY* genes in wheat.

## Introduction

The plant-specific *TIFY* TF gene family, previously known as ZIM (Zinc finger protein expressed in Inflorescence Meristem), constitute of four phylogenetic clusters: (i) TIFY (Threonine, Isoleucine, Phenylalanine, Tyrosine); (ii) JAZ (Jasmonate TIFY-domain); (iii) PPD (PEAPOD) and (iv) ZML (zinc finger protein expressed in inflorescence meristem, TIFY-like) proteins^1,2^. The highly conserved TIFY domain consisting of 36 amino acids is the characteristics the TIFY subfamily^1^. The TIFY domain comprises of conserved motif variants like TII[F/Y]XG, TLV[F/Y]XG, TLF[F/Y]XG, TIS[F/Y]XG, TLS[F/Y]XG, TMF[F/Y]XG, TLL [F/Y]XG and VIF[F/Y]XG. Glycine in the core motif TIF[F/Y]XG is highly conserved while the other hydrophobic amino acids are variable. Secondary structures of the TIFY domain follows alpha-alpha–beta fold arrangement. The JAZ subfamily orchestrates cross talks between Jasmonic Acid (JA) and other hormone signaling pathways, including auxins, gibberellins (GAs), abscisic acid (ABA), salicylic acid (SA), and ethylene (ET)^3,4^. This subfamily consists of a C-terminal conserved characteristic domain Jas (SLX2FX2KRX2RX5PY), which has sequence similarity to N-terminal of CCT domain^5,6^. The PPD subfamily consists of a unique PPD domain of about 50 amino acids at the N terminal. Additionally, a diverged Jas motif lacking the conserved amino acids PY is also present at the C-terminus^6^. Members of the ZML subfamily contain CCT domain, C2C2-GATA zinc finger domain, and the TIFY domain^7^.

Jasmonates are fatty acid-derived hormone molecules that regulate many plant physiological and stress-related processes like senescence, root growth, fruit maturing, wounding and pathogenesis^8,9^. JAs and their bioactive derivatives also control plant responses to abiotic stresses and exposure to ozone^10^. Over-expression of the *AtTIFY1* gene in *Arabidopsis* helped in elongation of petioles and hypocotyls^11^, whereas *AtTIFY4a* (PPD1) and *AtTIFY4b* (PPD2) enhanced leaf growth by controlling lamina size and leaf curvature^12^. In *Arabidopsis*, *TIFY* genes negatively regulate the key transcriptional activator of JA responses^13^. Additionally, JA signals integrate with other plant hormone signals such as those produced by auxins, ABA, ETs, GAs and SA, thereby modify diverse plant defense responses^14^. Coronatine, a bacterial phytotoxin is a structural and functional analogue to (+)-7-iso-Jasmonoyl-L-isoleucine (JA-Ile), the biologically active form of Jas; the Jas domain perceives JA-Ile and represses JA signaling pathway^15^.

Many TIFY proteins belonging to JAZ subfamily in *Brassica oleracea* were activated in response to Methyl Jasmonate (MeJa) but downregulated after SA/ET treatment or differentially modulated after *Fusarium oxysporum* infection^16^. Most *TIFY* genes belonging to the JAZ subfamily were activated in response to JA, salt and drought stress in watermelon^17^. Different JAZ subfamily genes displayed different levels of expression in the three cultivated cotton species *Gossypium hirsutum*, *G. barbadense* and *G. arboretum* in retort to Verticillium wilt due to *Verticillium dahliae* infection^18^. The JAZ genes also exhibited distinct expression patterns in the cotton seedlings treated with JA, MeJA, GA and ABA indicating their specific response to phytohormone signals in cultivated cotton species. The JAZ genes in tomato differentially responded to salinity and osmotic stress as well as were strongly induced in leaves and roots by JA and ABA^19^. So far, 18 TIFY genes had been identified in *Arabidopsis*^1^, 20 in rice^20^, 21 in *Brachypodium distachyon*^21^, 50 in *Gossypium hirsutum*, 54 in *G. barbadense*, 28 in *G. arboretum*^18^, 24 in *Populus tricocarpa*^21^, 20 in tomato^19^, 15 in watermelon^17^ and 36 in *Brassica oleracea*^16^ through bioinformatics-based approaches. Most of these identified *TIFY* genes, excepting Arabidopsis and rice, are not annotated. However, the discovery and functional annotation of this gene family in wheat is still limited.

Wheat (*Triticum aestivum* L.) is one of the most important cereal crops in terms of both cultivated area (~ 215 mha) and grain acreage (~ 765 million mt) in the crop year 2019–2020 worldwide (www.fao.org). In nature, most crop plants are exposed to multiple stress conditions either simultaneously or sequentially rather than by a single biotic or abiotic stress. Numerous biotic as well as abiotic stresses cause significant losses in wheat yield and reduce grain quality. Of the biotic stresses, the rust diseases, particularly the leaf rust, caused by the obligate biotrophic basidiomycetous fungi *Puccinia triticina* Eriks., is most prominent and occurs widely^23^. The disease is difficult to control as the pathogen continually develops new virulence profiles with high adaptability to wide agroclimatic conditions^24^. Among the abiotic stresses, drought, salinity, heat and waterlogging severely affect wheat production. The allohexaploid wheat genome (~ 16.94 Gb, AABBDD) originated from three closely related progenitor species, inflicted major challenges for molecular and functional genomics-based improvements^25^. A low (5 ×) coverage^26^ and of late a chromosome arm based high-quality reference genome sequence of wheat^27^ became available that provides the opportunity to study gene structures and regulatory functional networks in wheat. Availability of the genome sequences of two progenitors of hexaploid wheat, *Triticum urartu* and *Aegilops tauschii*, also unlocked sub-genome level exploration of wheat genes^28,29^.

Since JAs contribute modulation of defenses against both biotic and abiotic stresses in plants^30^, a better understanding of JA-controlled processes contributing to stress tolerance in wheat would provide substantial knowledge. Therefore, a comprehensive study was undertaken with the objective to identify and characterize the TIFY TFs in wheat.

## Results

### Identification and nomenclature of TIFY TF gene family in wheat

**Figure 1:**
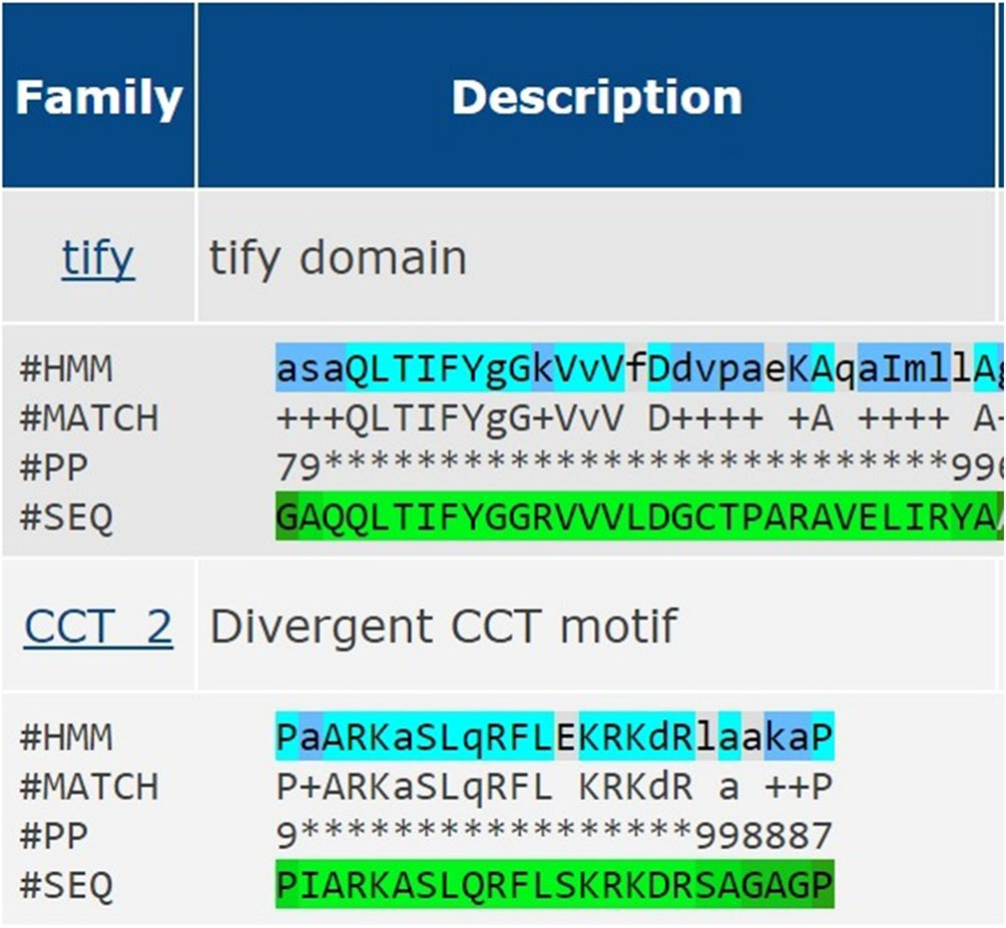
Conserved domain identified in TaTIFY proteins.

In order to generate a robust dataset of TIFY in wheat, a total of 45 completely annotated TIFY protein sequences of *Oryza sativa* (21)*, Sorghum bicolor* (06) and *Arabidopsis thaliana* (18)*,* were retrieved from Plant TFDB (Accession IDs provided in Supplementary Table 1). No wheat TIFY sequence was present in these databases. BLASTN with wheat genomic sequences provided 92 matches. These sequences were inspected for the presence of conserved TIFY (QLTIFYGGR) domain that provided 37 sequences. Of them, 23 sequences displayed complete ORF and were designated as putative novel TIFY genes in wheat. This also suggests that about four fifth, i.e., 103 out of initially identified 126 *TaTIFY* genes became non-operational in wheat, most likely due to transposon activity mediated genome rearrangements, which is very common in wheat^25^. The incomplete pseudo-genes were excluded (pipeline mentioned in Supplementary Figure S1). These novel genes were assigned unique identifier as *TaTIFY* (*Triticum aestivum TIFY*) followed by alpha numeric credential from TaTIFY1 to TaTIFY23 (Table 1). TaTIFY8 is the largest protein having 235 amino acids while TaTIFY 19 is the smallest having 57 amino acids. Five genes have their coding sequence in antisense orientation (Table 1). To further confirm, Pfam web server was used to examine the conserved domains. Twenty-one of these proteins have a TIFY domain with CCT2 motif (Jas) while TaTIFY4 and TaTIFY19 have only the TIFY domain (Fig. 1).

**Table 1:** Novel *TaTIFY* genes identified in wheat, their genomic location, ORF lengths, TIFY domain positions and orientation of coding sequences.

Name	Genomic location	ORF length (nucleotides)	TIFY domain position	Coding sequence orientation
TaTIFY1	2D:120255117–120255392	696	94–124	Sense
TaTIFY2	2B:356583353–356583742	342	1–27	Antisense
TaTIFY3	4B:582572166–582572902	626	67–99	Antisense
TaTIFY4	4B:582559524–582559901	549	79–111	Sense
TaTIFY5	4D:465601112–465601873	492	44–75	Sense
TaTIFY6	2D:120255119–120255342	696	19–51	Sense
TaTIFY7	5B:369635874–369636276	309	76–501	Antisense
TaTIFY8	5B:369635874–369636276	708	4–36	Sense
TaTIFY9	5B:369635874–369636276	751	9–41	Sense
TaTIFY10	2B:730999990–731000307	456	44–75	Sense
TaTIFY11	4A:4848198–4848894	230	67–99	Sense
TaTIFY12	4B:582572021–582572879	760	64–96	Antisense
TaTIFY13	2B:731001366–731001593	332	79–109	Sense
TaTIFY14	2A:124212259–124212534	651	56–88	Sense
TaTIFY15	7D:162629049–162629804	444	1–57	Sense
TaTIFY16	2B:356583355–356583691	332	76–105	Antisense
TaTIFY17	2D:120255243–120255366	623	27–59	Sense
TaTIFY18	2D:120255243–120255366	396	9–26	Sense
TaTIFY19	2B:731001677–731001774	174	27–59	Sense
TaTIFY20	2D:120255243–120255366	483	4–36	Sense
TaTIFY21	2D:120255243–120255366	472	54–86	Sense
TaTIFY22	5D:319504985–319505387	508	44–75	Sense
TaTIFY23	7D:162214514–162215156	233	55–88	Sense

### Phylogenetic analysis and classification of identified novel TIFY TFs

**Figure 2:**
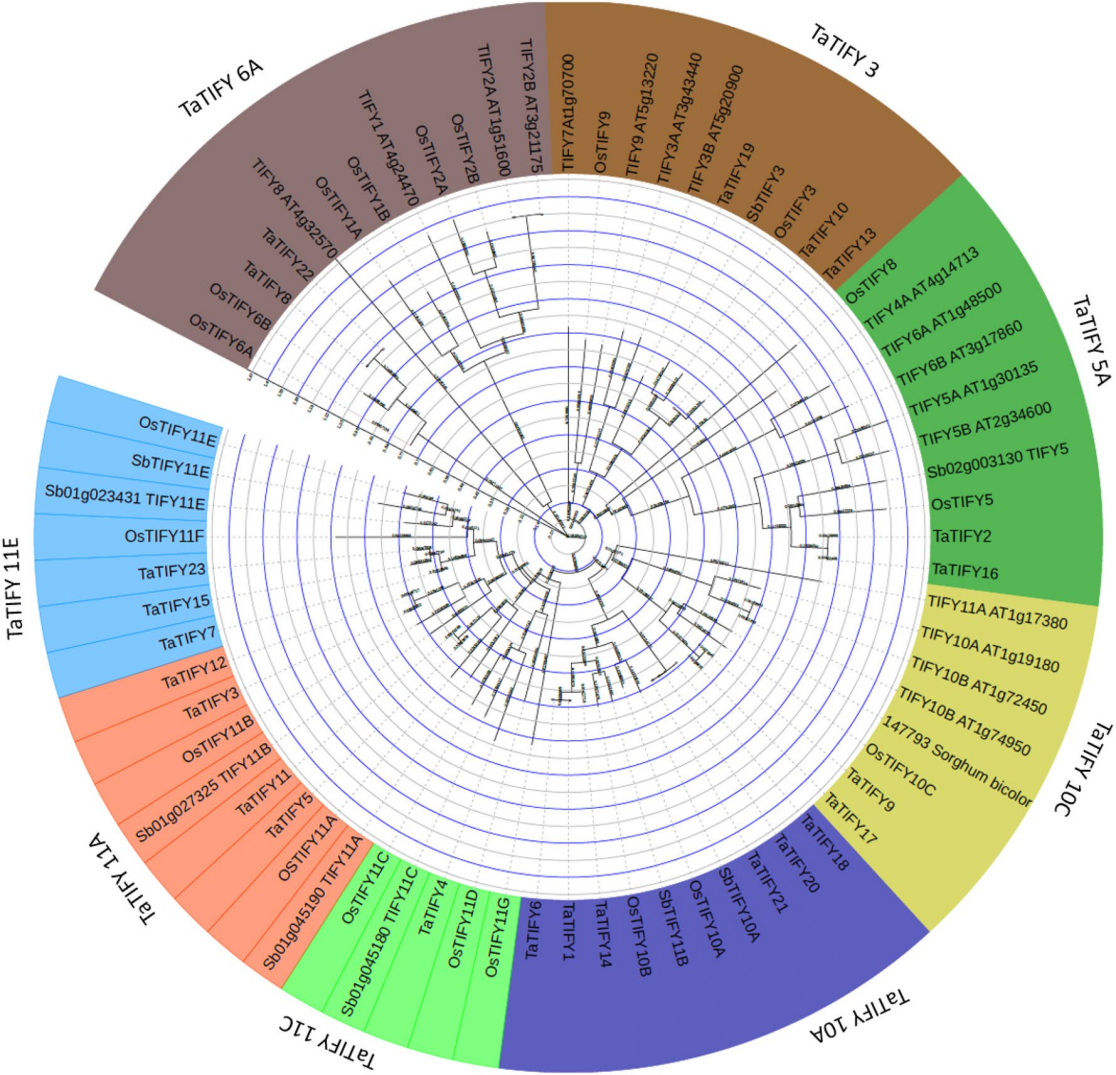
Phylogenetic relationships of 23 TaTIFY TF proteins of wheat and their evolutionary relationship with *Arabidopsis* and *Oryza sativa*.

MSA of the 23 identified TaTIFY sequences (Supplementary Figure S2) showed highly conserved motif QLTIFYGGR and PY domain at N-terminus of all sequences. To study evolutionary relationships among the TIFY, an unrooted tree was constructed to align the identified *TaTIFY* sequences with rice and *Arabidopsis TIFY* sequences since *TIFY* family members of these two species are well characterized (Fig. 2). Sequences of these three plants clustered into nine clades belonging to nine different subfamilies of TIFY according to the clade support value and topology of the tree^31,32^. The different subfamilies were: TIFY3, TIFY10A, TIFY10C, TIFY11A, TIFY11C, TIFY11E, TIFY5A, TIFY6A and TIFY11C (Fig. 2). Subfamily 10A contain six *TaTIFY* genes (*TaTIFY1*, *6*, *14*, *18*, *20*, *21*). Four genes belonged to TIFY11A (*TaTIFY3*, *5*, *12*, *13*) and three genes belonged to each TIFY11E (*TaTIFY*7, *15*, *23*) and TIFY3 (*TaTIFY10*, *13*, *19*). All these proteins had been characterized as salt and dehydration stress responsive genes in rice. Two genes belonged each to TIFY10C (*TaTIFY*9, *17*), TIFY6A (*TaTIFY8*, *22*), TIFY5A (*TaTIFY2*, *16*) and TIFY11C (*TaTIFY*3, 12), while one gene *TaTIFY*4 belonged to TIFY11C. Details of TaTIFY subclass distribution is provided in Table 1.

### Analysis of cis-acting elements and microRNA targets of *TaTIFY* genes

PlantCARE database revealed 40 *cis*-acting elements in *TaTIFY* promoter sequences many of which were involved in different stress responses (Supplementary Table S2). CAAT and TATA box, essential for RNA Polymerase II binding dependent transcription, were found in core promoter regions. *Cis*-acting phytohormone responsive elements (two elements responsive to MeJA, two for ABA, three for GA and one for SA signaling) had also been identified. Box W1, a fungal inducer receptive element along with other stress receptive elements (HSE, MBS, TC rich repeats) had also been identified that might have important roles in biotic stress. Several light responsive elements (as-2-box, GAG-motif, I-box, Sp1, G-Box, GT1-motif, MNF1and H-box) and anaerobic induction motifs (ARE, ABRE, C-repeat/DRE, GC-motif, LTR) were recognized. *Cis*-acting elements related with endosperm expression, seed germination and meristem were also detected.

Seventeen wheat miRNAs (tae-miR9780, tae-miR9677a, tae-miR5384-3p, tae-miR408, tae-miR1134, tae-miR1138, tae-miR9678-3p, tae-miR5384-3p, tae-miR1136, tae-miR399, tae-miR9657a-3p, tae-miR9653a-3p, tae-miR9669-5p, tae-miR171b, tae-miR9678-3p, tae-miR9670-3p, tae-miR531) were found to target the identified wheat *TIFY* genes (Supplementary Table S3). Both translation and cleavage inhibition modes were detected. The results revealed interesting observations that more than one miRNA targeted the same *TIFY* gene at different locations; conversely a single miRNA targeted more than one *TIFY* gene.

### Characterization of identified novel TaTIFY TF genes in wheat

The physico-chemical characterization of 23 TIFY proteins are provided in Supplementary Table S4. The theoretical pI values of all TaTIFY proteins (except TaTIFY9 and TaTIFY17) were greater than 7 and the average being 9.19, indicating that TIFY proteins were rich in basic amino acids. The instability index of a protein determines its structure and stability, values higher than 40 are considered unstable and those less than 40 are stable. The instability index of TaTIFY proteins ranged between 34.12 to 84.35. The aliphatic index, defined as the relative volume occupied by aliphatic side chain and determines thermo-stability of globular proteins, were found between 60.19 and 89.19 indicating their high thermal stability and flexibility. The Grand Average of hydropathicity (GRAVY) value, an indicator of hydrophilic or hydrophobic nature of the protein, varied from − 0.487 to + 0.095, the normal range of GRAVY being ± 2. Two proteins (TaTIFY 12 and 19) are hydrophobic in nature and the remaining 21 were hydrophilic in nature. Glycosylation indicates attachment of sugar moieties to the proteins through post translational modifications. Any potential crossing the threshold of 0.5 predicts glycosylation. N- and O-glycosylation sites had been identified in all 23 TaTIFY TF proteins. N-linked glycosylation sites were present in 18 TaTIFY proteins whereas O-linked glycosylation sites were present in all TaTIFY proteins (Supplementary Table S4).

Analysis of catalytic domains revealed 17 different motifs (Supplementary Table S5). The TIFY domain was invariably present in all TaTIFY proteins. Another highly conserved motif CCT2 (Jas) was found in all TaTIFY proteins except TaTIFY18 and 19. Besides, domains with functions in amidation (receptor recognition and signal transduction), phosphorylation of cyclic AMP (glycogen regulation, sugar and lipid metabolism), casein kinase2 phosphorylation domain (acidic protein phosphorylation), myristylation domain (membrane targeting and signal transduction in plants in response to various environmental stresses), alanine rich domain (stability of tertiary structure), protein kinase C domain (signal transduction cascade), ANS_Glycosylation domain, NLS_BP domain and arginine-rich domains were also identified. Specific domains were also identified in certain TaTIFY proteins. FARP motif (FARFamide related peptide) in TaTIFY17 and 22, DUF (DNA unwinding factor) in TaTIFY12 and 22, ChEC (Chromatin endogenous cleavage) in TaTIFY8 and 16, CCT (CONSTANS, CO-like, TOC1) in (TaTIFY5, 7, 8, 10, 11, 13, 16, 17, 20, 21, 22 and 23).

Two types of Nuclear localization signals (NLS) had been identified: monopartite (single stretch of basic amino acids) and bipartite (two stretches of basic amino acids) (Supplementary Table S6). Two proteins (TaTIFY5 and 11) had monopartite NLS, 20 proteins had bipartite NLS whereas one protein, TaTIFY10 did not contain any NLS.

### Identification of conserved motifs, secondary structure prediction and sub cellular localization

Ten most conserved motifs were identified in TaTIFY proteins (Supplementary Table S7). TIFY, the signature motif, was present in all 23 TaTIFY proteins; other motifs included QLTIFYGGK, KRKDRLHAKAPY, MTIFYNGR, ELGLGINKGE, NHEESLRLGR, PQSVGFSIKD, KGSPVVQN, KGSPVVQNVALPQPS, NASDLPIARKASLH (Supplementary Figure S3). The variations in the composition of different motifs present in TaTIFY proteins show their functional diversification in relation to different aspects of biological processes they regulate.

Secondary structure of TaTIFY proteins was predicted using a feed-forward neural network which performs analysis on outputs obtained from PSI-BLAST. The number and positions of helices and strands present in all TaTIFY proteins were determined. All proteins formed an alpha-alpha–beta fold, the characteristic of TIFY family (Supplementary Figure S4).

MEMSAT tool on PSIPRED server predicted trans-membrane structure and topology: eight TaTIFY proteins had pore lining helices and their respective positions are shown in Supplementary Figure S5. Proteins disordered state was predicted in all 23 proteins using DISOPRED2 tool (Supplementary Figure S6). Disorder is functionally important to be associated with recognition and binding.

Sub-cellular localization for all 23 newly identified TIFY TF protein sequences in wheat predicted 12 proteins (TaTIFY 1, 3, 6, 9, 12, 15, 17, 18, 19, 20, 21 and 23) to be localized outside nucleus in organelles and the remaining 11 proteins were localized within nucleus as well as in cytoplasmic organelles (Supplementary Table S8).

### Tertiary structure prediction and validation

**Figure 3:**
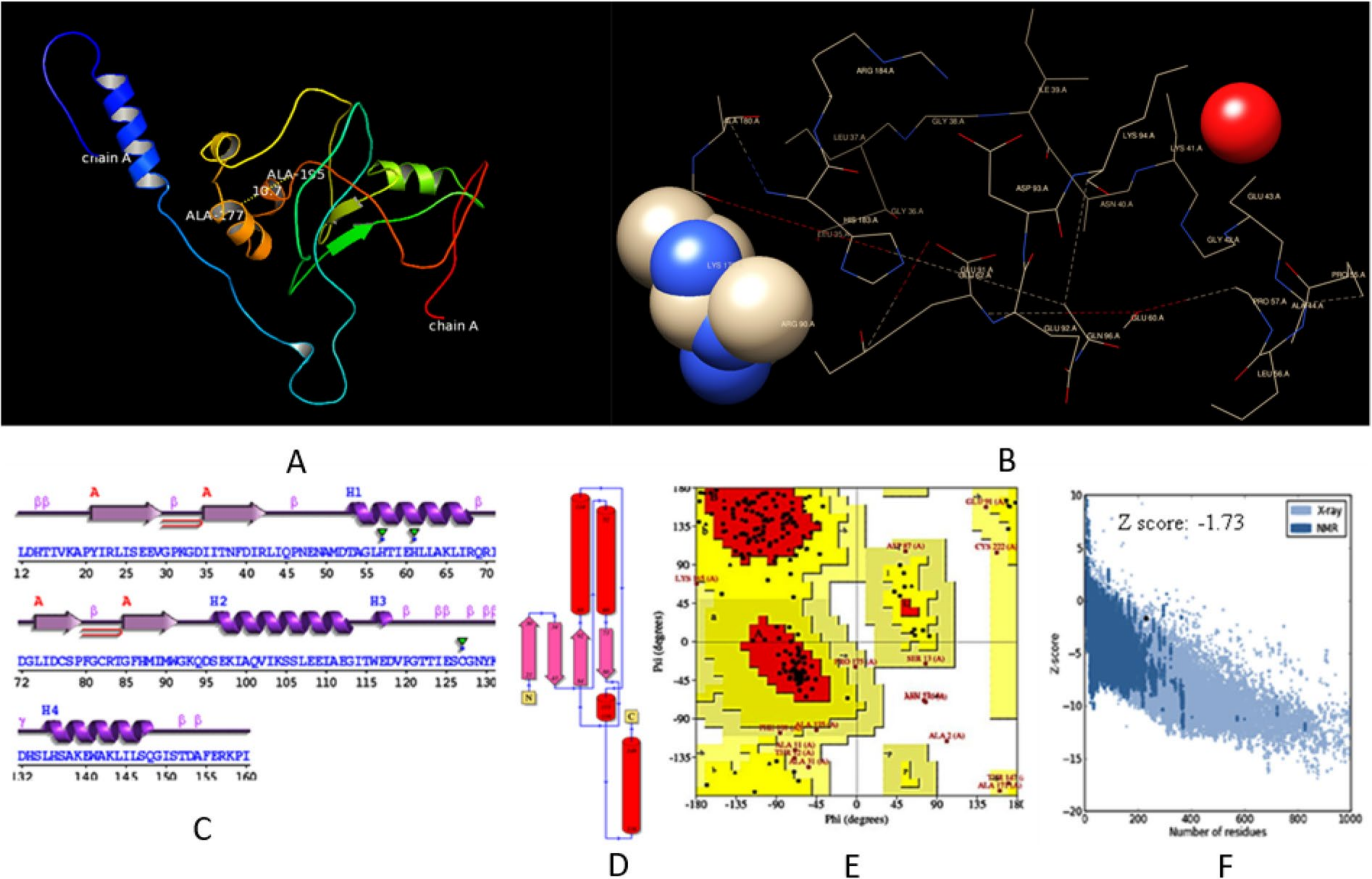
Tertiary structure of *TaTIFY*1 gene (**A**) showing a single chain of 231 amino acid having active site between Ala 117 and Ala 195 with atomic distance 10.7 Å. (**B**) Detailed structure of TaTIFY1 transcription factor showing major DNA binding site (Volume—2497.02 [Å^3^], Surface—3516.88 [Å^2^], Lipo surface—2343.34 [Å^2^], Depth—26.66 0.68[Å] Simple Score—0.68). Validation of stereo-chemical property of the tertiary structure of TaTIFY1 through (**C**). PDBsum server secondary structure, (**D**) wiring diagram, (**E**) Ramachandran plot, (**F**) ProSA web z-scores.

For better characterization of TaTIFY proteins, the translated sequences were subjected to ab initio modeling and threading using I-TASSER server. The server generated five 3D atomic models for threading and iterative structural assembly simulations. The 3D models were predicted by ten threading templates with PDB hits. The most apt secondary structure was chosen based on maximum C-score (confidence score), TM-score (topological similarity score), RMSD (Root Mean Square Deviation), cluster density and was subjected to internal evaluation of self-consistency tests. Tertiary structure of TaTIFY1 is provided in Fig. 3, and rest in Supplementary Figure S7.

The stereo-chemical quality and reliability of predicted TaTIFY models were validated by subjecting the PDB files to PROCHECK and PDBsum server (TaTIFY1 is provided in Fig. 3B-E, and rest in Supplementary Figure S8). The Ramachandran plot statistics depicted > 90% residues in most favorable combination of phi/psi values assuring high quality attributes of TaTIFY protein structures. The ProSA server displayed graphs containing z-scores of negative values of the models with comparable protein structures in PDB indicating the reliability of the structures. The binding sites of the proteins were predicted by DoGSiteScorer. The first two binding pockets were considered as prominent active sites (TaTIFY 1 in Fig. 3E; rest in Supplementary Figure S8, Supplementary Table S9).

### Gene ontology and enrichment analysis

**Figure 4:**
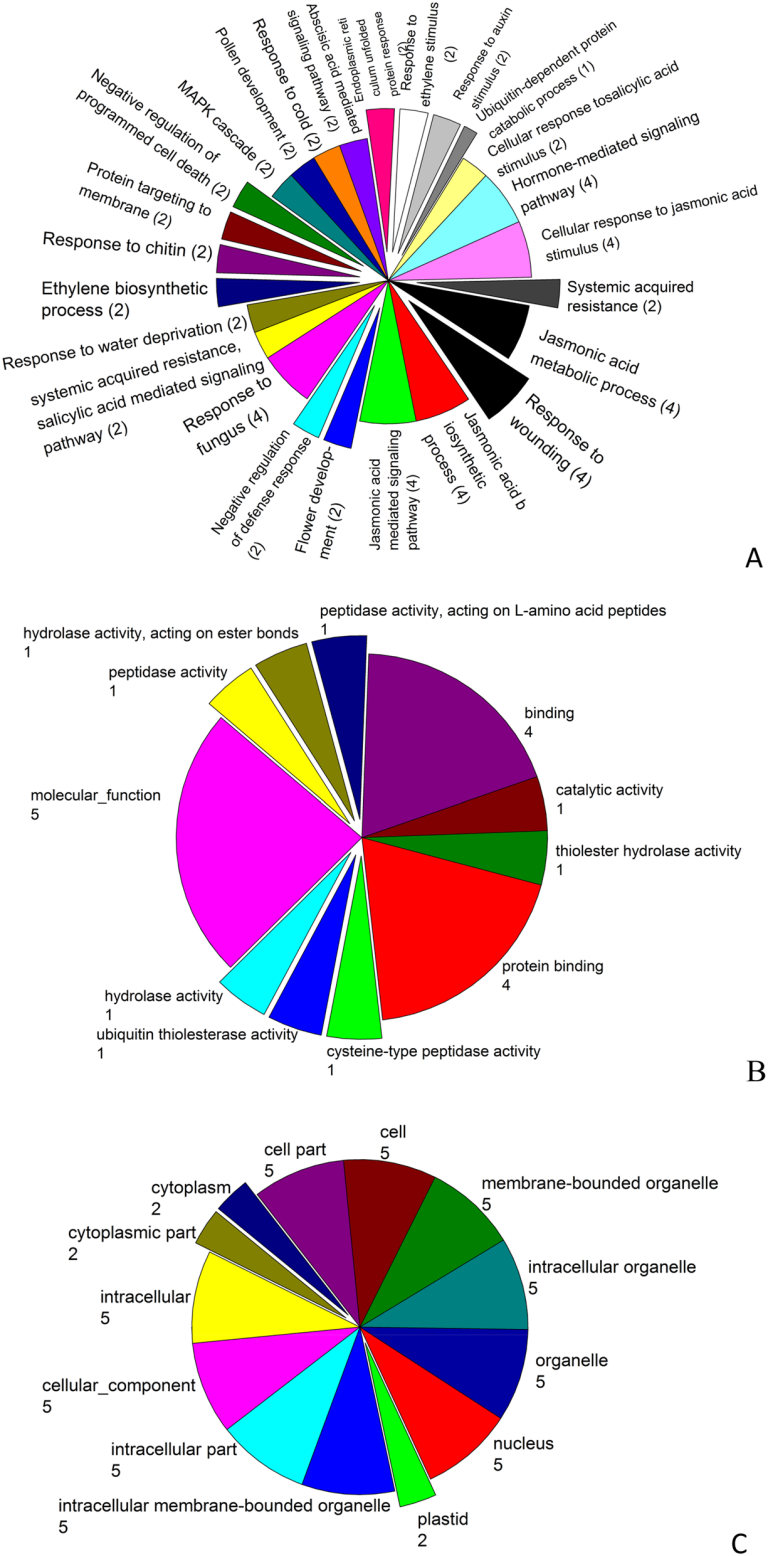
Gene Ontology term enrichment analysis of *TaTIFY* genes, (**A**) biological processes, (**B**) molecular function and (**C**) cellular component.

Blast2GO analysis classified TaTIFY genes into three main categories: biological process, molecular function and cellular component (Fig. 4). The biological processes associated GO terms were regulation of transcription (21 terms), ubiquitin dependent protein catalytic process (1), systemic acquired resistance (2), SA mediated signaling pathway (2), response to wounding (4), response to water deprivation (2), response to ethylene stimulus (2), negative regulation of defense response (2), JA mediated signaling pathway (4), JA biosynthesis process (4), hyperosmotic salinity response (2), flower development (2), defense response to fungus (2), defense response to bacteria (2), ABA mediated signaling pathway (2) etc. (Fig. 4A). The molecular function category included binding (23 terms: to DNA, protein, ATP, ADP), catalytic activity (6 terms), ubiquitin thiol esterase activity (15 terms), cysteine type peptidase activity (1 term) (Fig. 4B). The cellular component category included nucleus, cytoplasm, cytosol, plastid, mitochondria, chloroplast envelop, chloroplast, stroma, transcription factor complex (Fig. 4C). WEGO outputs of the enriched GO terms are shown in Supplementary Figure S10.

### Chromosomal localization and synteny analysis

**Figure 5:**
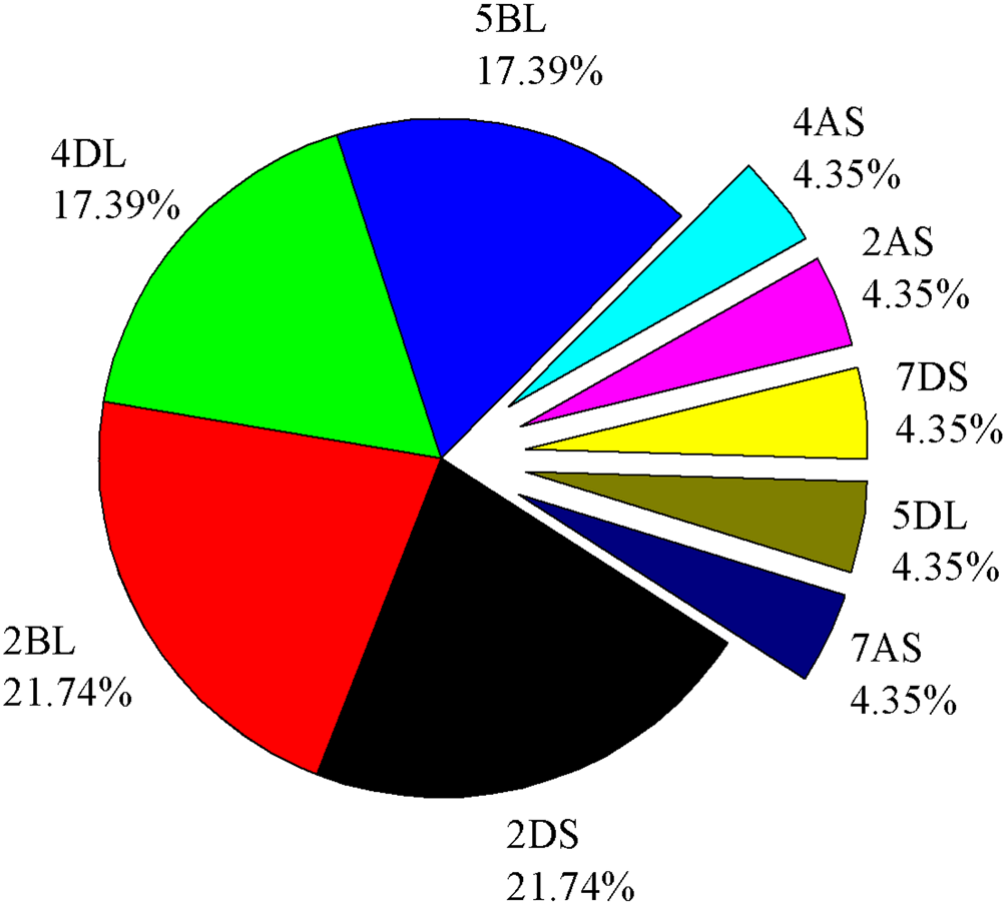
Distribution of the identified *TaTIF*Y TF genes on wheat chromosomes.

**Figure 6:**
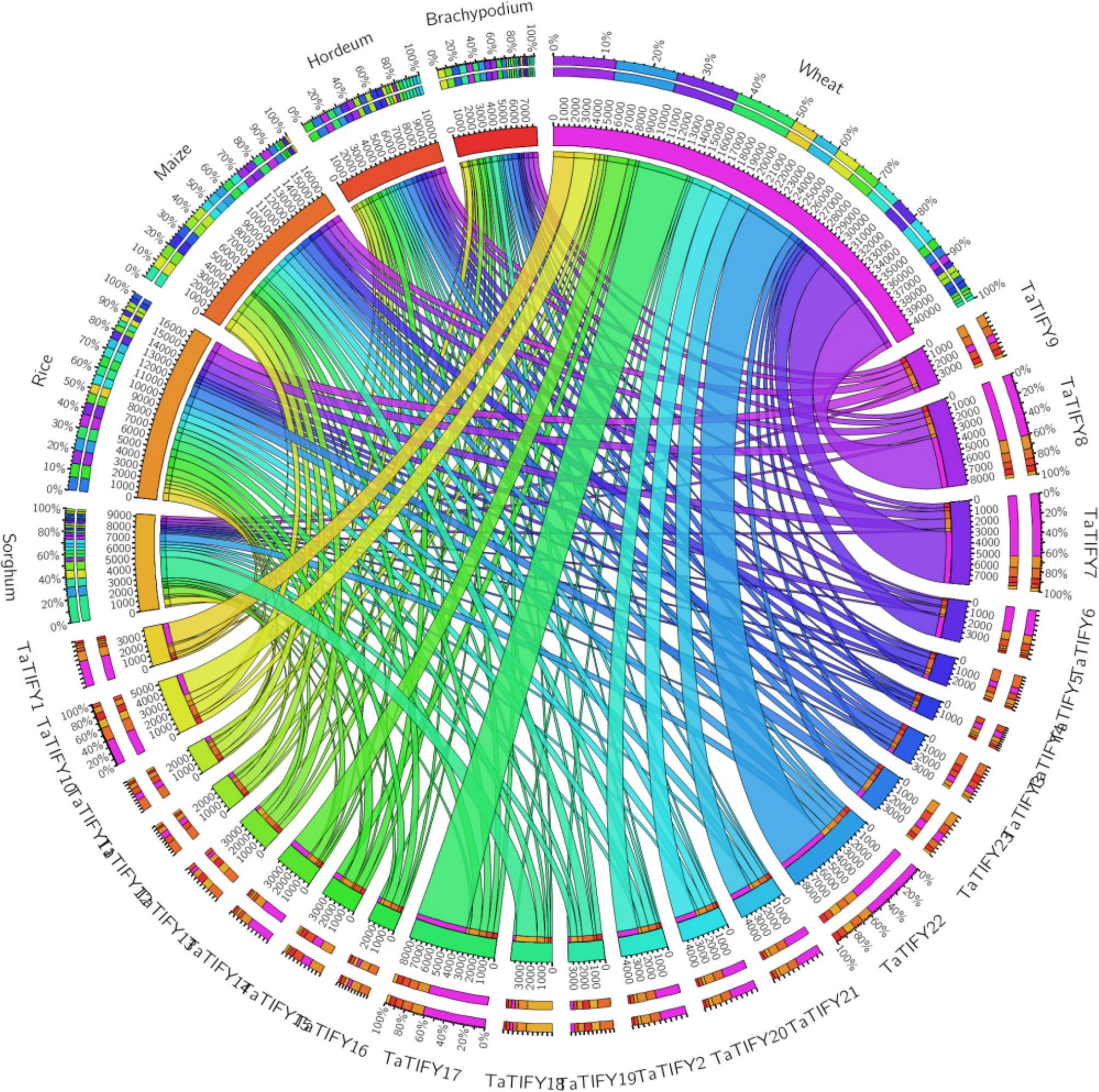
Comparative synteny and expansion analysis of *TaTIFY* TF genes with sorghum, rice, maize, *Hordeum* and *Brachypodium* based on orthologous and paralogous pair positions that demonstrates highly conserved synteny.

**Figure 7:**
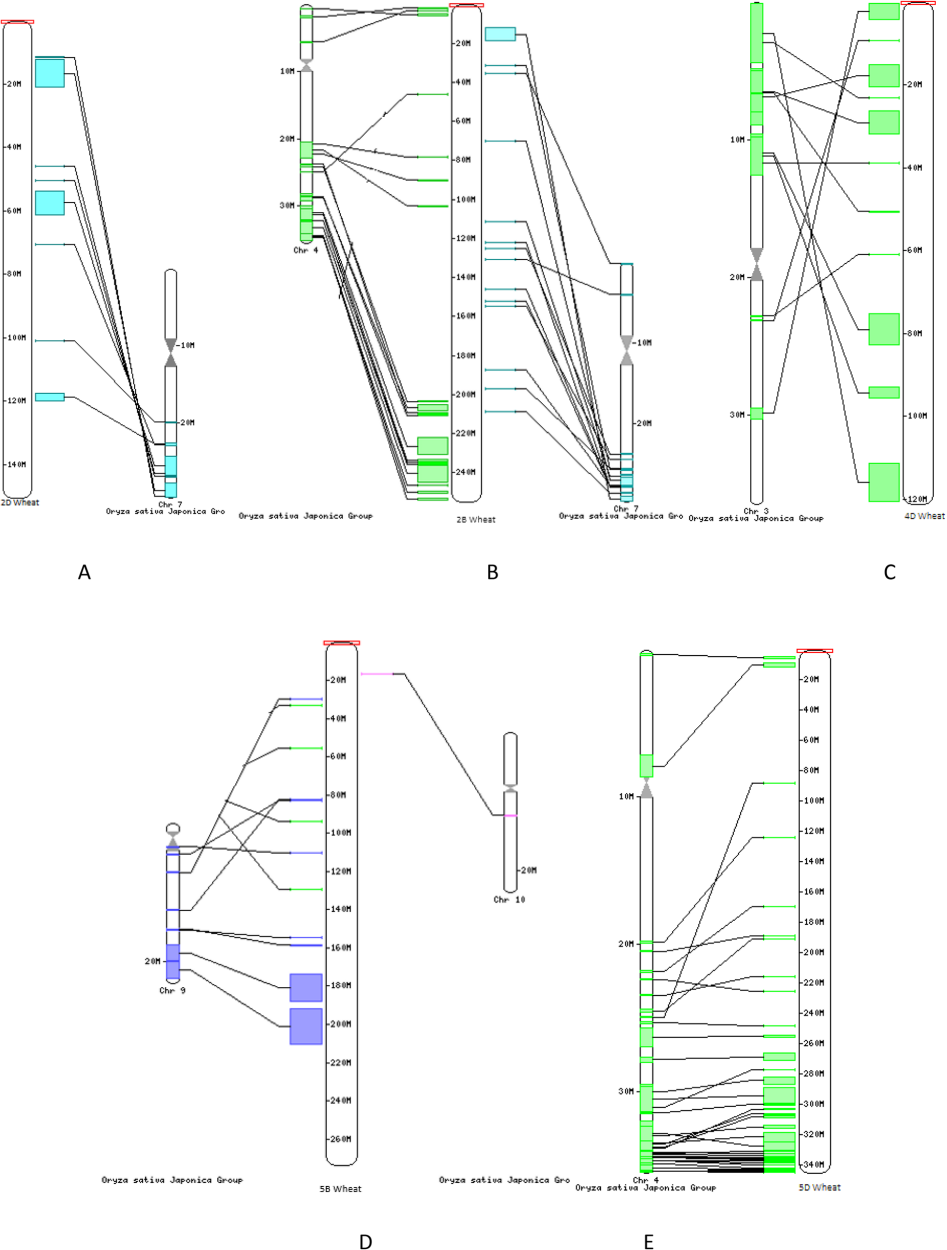
Comparative positional analysis of TaTIFY genes between wheat and rice chromosomes. (**A**) Comparative localization of TaTIFY genes (1, 6, 18, 20) on 2D of wheat and chromosome 7 of rice, (**B**) Comparative localization of TaTIFY genes (2, 10, 13, 16, 19) on 2B of wheat and chromosome 4 and 7 of rice, (**C**) Comparative localization of TaTIFY genes (3, 4, 5, 12) on 4D of wheat and chromosome 3 of rice, (**D**) comparative localization of TaTIFY genes (7, 8, 9, 17) on 5B of wheat and chromosome 9 and 10 of rice, (**E**) Comparative localization of TaTIFY21 on 5D of wheat and chromosome 6 of rice.

The *TaTIFY* genes mapped on wheat chromosomes with a clear non-random distribution (Fig. 5; Supplementary Table S10). Syntenic analysis revealed all *TaTIFY* genes had orthologous sequences in sorghum, rice, maize, barley and *Brachypodium* indicating presence of similar TIFY domains in monocots (Fig. 6). Comparative synteny indicated *TIFY* sequences shared 55–96% identity. Considering that orthologs with more than 55% identity often retain equivalent functions during evolution, we examined the orthologous relationship between *TaTIFY* genes with the other monocots using Circos software. *TaTIFY* genes (1, 6, 18 and 20) located on chromosome 2DS in wheat were found to be located on chromosome 1 of *Brachypodium*, 7 of rice, 2 of barley, chromosomes 1 and 2 of sorghum, and chromosome 1, 2 and 7 of maize respectively. The crucial point is that all four *TIFY* genes are located on a single chromosome of rice, barley and *Brachypodium* while on more than one chromosome of sorghum and maize. Thus, it can be inferred that these four TaTIFY genes are highly conserved in rice, barley and *Brachypodium* in comparison to the other two species. TaTIFY genes (2, 10, 13, 16 and 19) located on chromosome 2BL in wheat were distributed on chromosome 2 of barley and different chromosomes of the other monocot species (Fig. 6, Supplementary Table S11). Detailed synteny between *Oryza sativa* japonica and *Triticum aestivum* showed highly conserved *TIFY* gene distribution (Fig. 7).

### Molecular cloning, sequencing, and sequence characterization

Representative genomic and cDNAs were cloned from different clades of *TaTIFY* genes (Supplementary Figure S11) using primer pairs (Supplementary Table S12) and sequences were submitted to NCBI (Table 2). Pairwise alignment revealed intronic region in four *TIFY* genes (TaTIFY3, 19, 20, 23; Supplementary Figure S12). The physico-chemical characterization of deduced proteins (Supplementary Table S13) revealed high similarity with the in silico identified proteins. Some unique catalytic domains like PAAR, FARP, ADF-H and DUF 2149 were revealed (Supplementary Table S14). Bipartite NLS were identified in all proteins whereas monopartite NLS were found in four proteins (Supplementary Table S15). Secondary structure of these proteins exhibited the number and positions of helices and strands (Supplementary Figure S13, Supplementary Table S16). TaTIFY5 had the highest number of α-helices (6) while TaTIFY3 had five β-strands. All proteins had disordered state implicating roles in protein-DNA interactions (Supplementary Figure S14). Pore lining helices were identified in TaTIFY3 and TaTIFY5 (Supplementary Figure S15).

**Table 2:** Accession numbers of cDNA and genomic DNA of TIFY sequences submitted at NCBI.

Sl no.	Gene name	Subgroup	NCBI Accession numbers	
			cDNA	Genomic DNA
1	TaTIFY19	TIFY3	MH277603	MH151794
2	TaTIFY20	TIFY10A	MH161186	MH109179
3	TaTIFY9	TIFY10C	MH211595	MF059098
4	TaTIFY3	TIFY11A	MH161187	MH124204
5	TaTIFY5	TIFY11B	MH290739	MH136799
6	TaTIFY23	TIFY11E	MH151793	MH142187

Blast2GO revealed GO terms associated with biological process like metabolism, cellular process, response to stimulus, development, localization, multicellular organismal process, while molecular functions were related to catalytic activity and binding, specifically to nucleic acids (Supplementary Figure S16). Genome wide syntenic relationships of these TFs with other monocots (Rice, Sorghum, Maize, Barley and *Brachypodium*) displayed orthologous relationships (Supplementary Figure S17). The analysis showed high conservation of *TaTIFY23* among wheat, maize and *Brachypodium* while *TaTIFY3* between wheat and barley indicating maximum orthology between wheat and barley. 

Protein–protein interactions among TIFY with other proteins were studied using STRING database functional links^33^. Since wheat genome sequence became only recently available in the public domain, it is not present in this database. Therefore, we functionally annotated these proteins in rice, sorghum, *Brachypodium* and maize. The results were displayed as interaction networks of TIFY with their functional partners in a variety of functions like binding to different DNA binding domains, activation of transcription factors and post translation modifications (Supplementary Figure S18). Some important interacting proteins included JAZ 11 or TIFY 3A of *Arabidopsis*, which are repressors of JA responses, ZIM3 of *Zea mays*, MYC4-like protein, bHLH protein etc. They were specifically involved in activation of GID1 (GA Insensitive Dwarf1), a gibberellin receptor. Functional partners for binding included B3 DNA binding domain, bZIP TF, helix loop helix DNA binding domain which were mainly involved in inactivation of GID2. Gene occurrence studies revealed the organisms in which these proteins were highly conserved since functional partners often have similar occurrence pattern. These proteins were highly conserved in Streptophyta which included green plants comprising charophyceae (streptophyta green algae) and embryophyta (land plants) (Supplementary Figure S19).

### Study of expression of TIFY family TFs during biotic stress and abiotic stress

**Figure 8:**
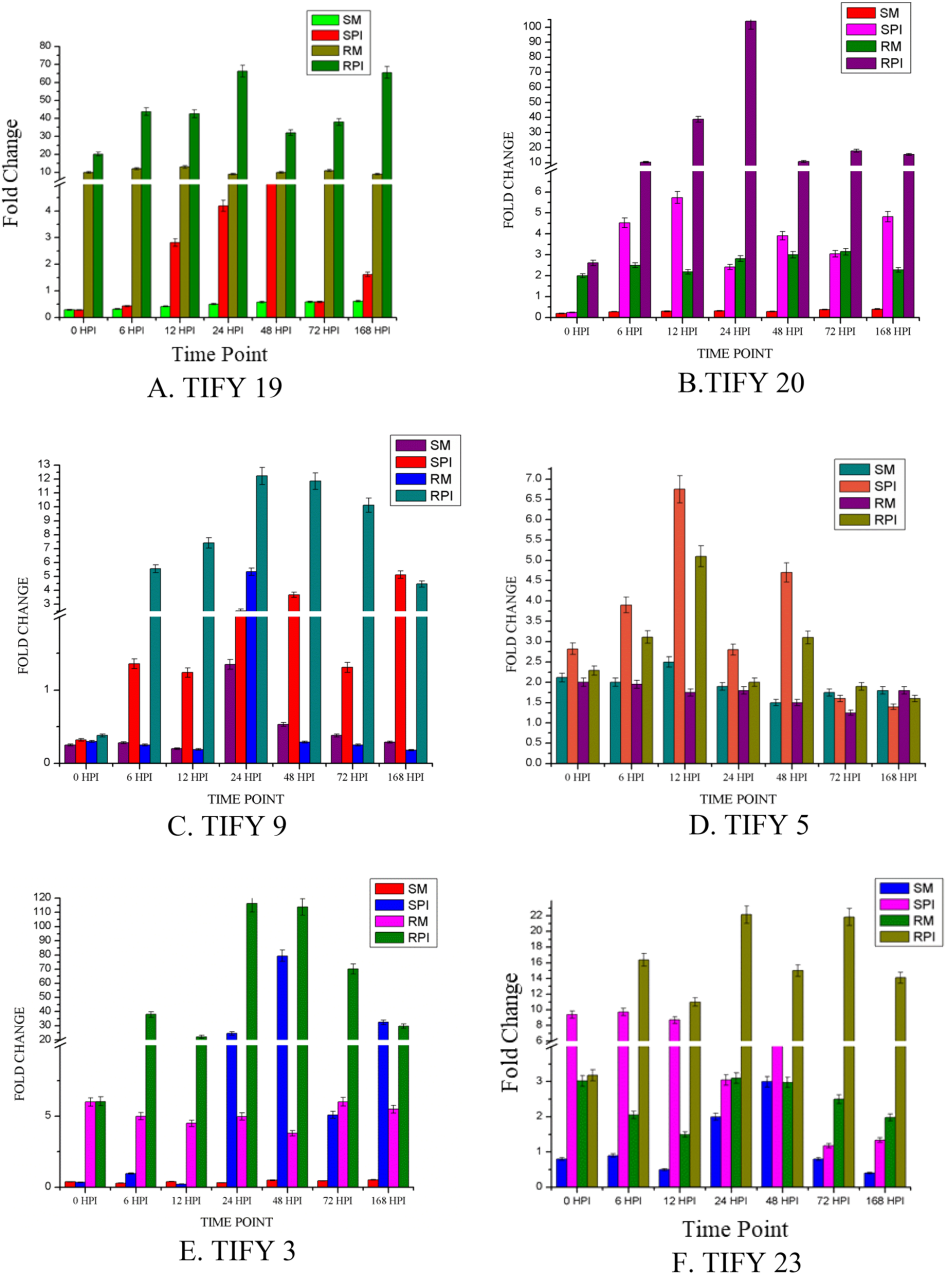
Spatio-temporal expression of *TaTIFY* family genes in response to biotic stress. Expression profiles of susceptible (HD2329) and resistant (HD2329 + *Lr28*) wheat plants in response to leaf rust infection compared with mock inoculated controls. Relative expression is expressed as fold changes relative to mock inoculated controls. (S-M, susceptible mock; S-PI, susceptible pathogen inoculated; R-M, resistant mock; R-PI, resistant pathogen inoculated).

QRT-PCR studies revealed the potential roles of TIFY genes during compatible and incompatible interactions involving the leaf rust pathogen. The time-points specific for development of leaf rust infection structures^34^ produced by pathogen were correlated with TIFY expression patterns. The spatial and temporal expression patterns were compared between mock and pathogen inoculated susceptible and resistant NILs. Highly increased expression was observed in the resistant NIL after infection relative to susceptible NIL and mock-inoculated controls (Fig. 8).

Reduced expression of most *TaTIFY* genes was observed in both NILs just before infection (0 hpi). During incompatible interaction, expression was highly induced from 6 hpi onwards. The period of maximum expression at 12, 24 and 48 hpi related to the phase of propagation and spread of secondary hyphae to adjacent cells. At 72 hpi surface mature appressoria collapses and intercellular ramification of secondary infection hyphae starts. To check the movement of infection to adjoining cells, high level expression persists till 168 hpi to prevent the expansion of SSVs in neighboring cells. While susceptible plants displayed much reduced expression. Negligible changes in expression profiles in mock inoculated susceptible and resistant plants were found due to absence of pathogen pressure.

The difference in expression pattern between pathogen and mock inoculated resistant and susceptible NILs was determined using Wilcoxon signed rank test, a non-parametric distribution free test used to illustrate null hypothesis that revealed significant differences (Supplementary Figure S20; Supplementary Table S17). The significance of difference in expression of *TIFY* genes were evaluated using ∆Ct values at p ≥ 0.01 (Supplementary Tables S18A-F). The results revealed significant differences in expression between mock and pathogen inoculated susceptible and resistant cultivars.

**Figure 9:**
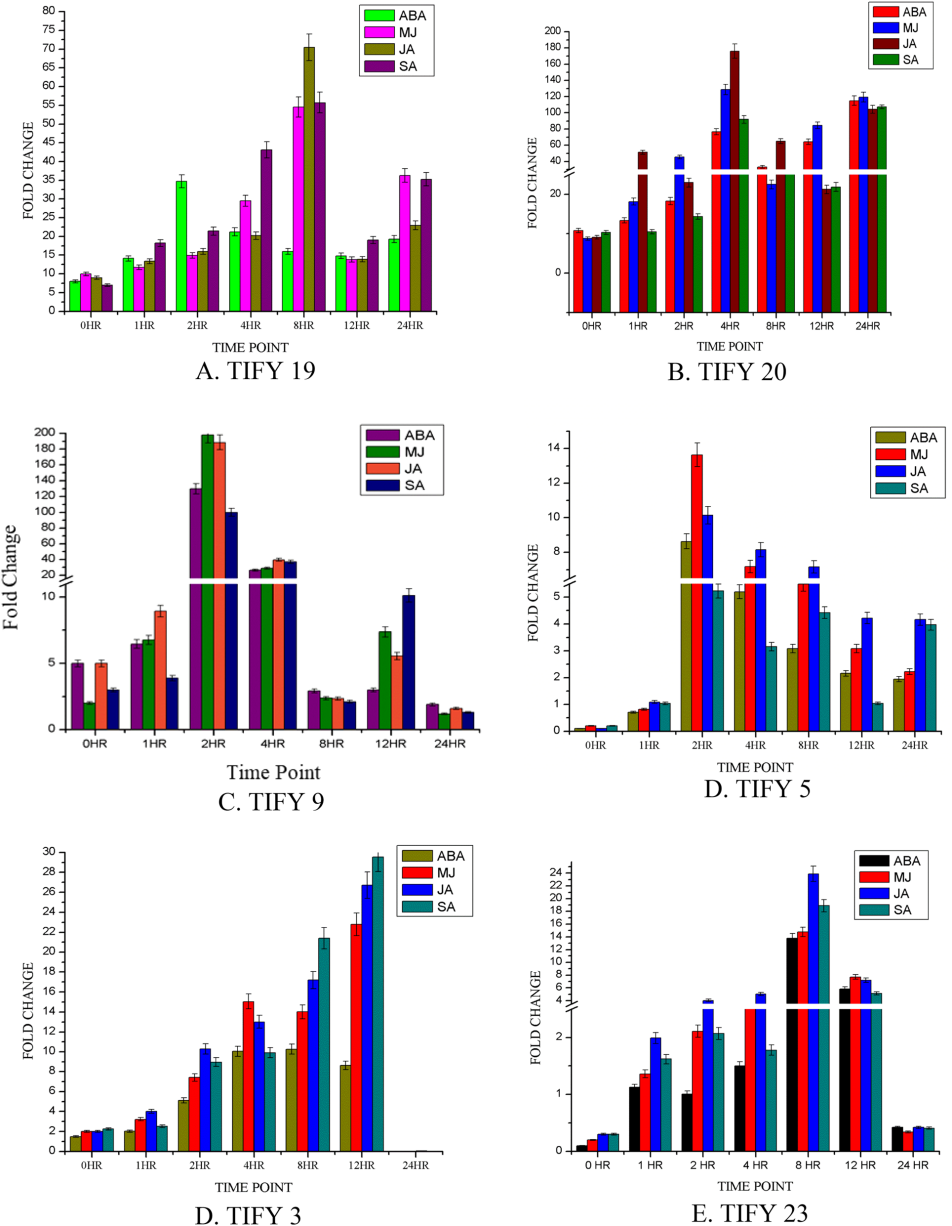
Spatio-temporal expression of *TaTIFY* family genes during abiotic responses.

QRT-PCR was also performed for *TIFY* genes under different abiotic stresses (Fig. 9; Supplementary Table S19). Most *TIFY* genes were highly induced at two and four hours post phytohormone treatment, highest being for MJ (198-fold) and JA (189-fold) for TIFY9 followed by JA (176-fold) and MJ (129-fold) for TIFY20. Their expression decreased after 12 h. It can be inferred that different *TIFY* genes respond differently to different phytohormones and the response was mediated through JA pathway since maximum expression was observed during JA and MJ treatment followed by SA.

The significance of differences observed in different *TIFY* genes expression during different phytohormone treatments and was assessed using ∆Ct values (Supplementary Tables S20A-F). The correlation between different phytohormones used in this study was established using Spearman's correlation rank-order, a non-parametric version of the Pearson correlation. The correlation coefficient (*ρ*, also signified by r_s_) measures the strength of association between two ranked variables accepting values from + 1 to − 1. A r_s_ of + 1 indicates a perfect association of ranks, a r_s_ of zero indicates no association between ranks and a r_s_ of − 1 indicates a perfect negative association of ranks. Highest correlation was found in TIFY5 (ABA and MJ, ABA and JA and MJ and JA); TIFY3 also showed high correlation (Supplementary Table S19). The distinct spatio-temporal expression pattern of *TIFY* genes during phytohormone treatments demonstrates their positive roles during abiotic stress in wheat plants.

## Discussion

Knowledge of definite TF repertoires in specific plant species provides insights into the diverse roles it contributes to the plant. Besides TFs, plant hormones are also involved in many aspects of plant growth, development, and response to environmental cues. As a result, cross talks between signaling pathways of various phytohormones and TFs balance development and stress responses in plants. The TIFY families of TF proteins play pivotal roles in various developmental and physiological processes as well as responses to biotic and abiotic stress conditions in plants.

In this study, a total of 92 TIFY genes had been initially identified, of which 23 genes had complete ORF containing the TIFY domain. Since limited resources of *TIFY* sequences are available at different TFDBs, in silico data mining represented an effective approach to identify and predict putative functions of TIFY TF families in wheat. After careful comparison at Pfam database together with TIFY from other plants we found that CCT2 and TIFY domains were highly conserved in these proteins. Based on this observation, TaTIFY TFs were classified into two sub-families JAZ and TIFY. An N-terminal Jas (CCT2) domain characterizes JAZ proteins. This domain interacts with MYC2, a bHLH transcription factor, to inhibit JA response together with TIFY domain^6^. *TaTIFY* genes were classified into nine different sub-families which were involved in stress responses in rice and *Arabidopsis*. As genes with sequence similarities generally have similar functions across different species, so the identified *TIFY* genes could be predicted to have similar functions in wheat also. Thus, construction of the phylogenetic tree of *TIFY* genes among different species provided plausible functions of the newly identified TIFY TF proteins in wheat.

Chromosomal synteny is important in genome comparison to reveal genomic evolution of related species^35^. Although it cannot be proclaimed with high accuracy, certainly gene duplications had played important roles leading to series of genomic rearrangements and expansion of this gene family among the different monocot species as manifested by synteny analysis. Shared synteny denotes the genomic fragments in different species had originated from identical ancestor^36^. With well-defined synteny relationships between well studied genomes (such as *Oryza sativa*, *Brachypodium distachyon*, *Hordeum vulgare*, *Zea mays* and *Sorghum bicolor*) and newly sequenced genomes like *T. aestivum* provide knowledge on differentiation of the less-studied gene family like *TIFY* after species divergence. More importantly, it helps improve the gene annotation of newly sequenced genomes.

We propose a model depicting the function of *TIFY* TFs in wheat built on *P. triticina* induced biotic stress response analysis (Supplementary Figure S21A). Based on our earlier study^37^ and published literature on histo-pathological data^34^ we correlated the expression of *TIFY* genes during pathogen infestation. The pathogen induces mechano-sensory reactions when encounters leaf surfaces, thereby activating local defense responses that stimulate systemic responses, like early responsive genes that in turn activate different defense and stress responsive genes. The outcome is stimulation of downstream responses in terms of increased inhibition of fungal growth, disease resistance and development of hypersensitive responses depending upon the nature and function of up- or down-regulated genes and their cross talk between infested and neighboring cells.

Various plant growth regulatory phytohormones are also involved in plant responses to biotic and abiotic stresses. The phytohormone network has the regulatory potential that allows plants to quickly react to changes environment, thereby modulate plants to efficiently use available nutrient resources. In nature, plants being sessile, are exposed to many biotic and abiotic stresses. Hormone homeostasis is critical in the establishment of appropriate and effective defensive responses in plants against natural attackers and abiotic stresses (Supplementary Figure S21B). The interaction between these two types of environmental stresses requires a complex adaptive molecular response involving many factors^38^. The significant differences between the NILs regarding gene expression can be ascribed to the *Lr28* seedling leaf rust resistance gene which enabled deterrence of leaf rust infection in resistant wheat plants.

It is well established that SA, JA and ET signaling is involved in the regulation of plant-pathogen interactions. SA-mediated plant responses generally govern biotrophic pathogens while JA and ET regulate plant responses to necrotrophic pathogens. Whereas ABA-mediated plant responses are commonly associated with abiotic stresses. Various cross talks between *Arabidopsis* and its necrotrophic fungal pathogen *Botrytis cinera* revealed the involvement of eliciting molecules like cyclopentenones to modulate several stress-responsive transcription factors including WRKY33^39^ and RAP2.4^40^. Since TIFY TFs administer regulation of a variety of phytohormones involved in biotic and abiotic stresses, this study uncovered the dynamic roles of TIFY genes.

In conclusion, TIFY TFs proteins regulate a wide range of biological processes, with their most distinctive role in plant defense response. To the best of our knowledge, this is the first study of structural and functional attributes of *TIFY* TF genes in wheat. Generally, TFs within same groups show recent common evolutionary relationships and conserved specific motifs that are related to similar molecular functions^41^. The close relationship between wheat, rice and *Arabidopsis* permitted the identification of highly homologous *TIFY* genes that allowed predicting their probable functions in wheat. This study provides useful information for future studies of TIFY TFs in regulation of wheat growth and development as well as for their incorporation into wheat breeding programs.

## Materials and methods

### Identification and nomenclature of *TIFY* gene family in wheat

To identify sequences belonging to *TIFY* gene family in wheat, TIFY protein sequences of *Arabidopsis thaliana*, *Sorghum bicolor*, and *Oryza sativa* (both *indica* and *japonica* subspecies) were downloaded from Plant Transcription Factor Database (Plant TFDB; http://plntfdb.bio.uni-potsdam.de/v3.0/). The wheat genomic sequences (https://www.wheatgenome.org/Tools-and-Resources/IWGSC-RefSeq-v2.0) were obtained from Ensembl Plants (https://plants.ensembl.org/Triticum_aestivum/) and translated into six reading frames using EMBOSS version 6.6.0.0. (ftp://emboss.open-bio.org/pub/EMBOSS/). The retrieved TIFY protein sequences were used for similarity search with the translated wheat genomic sequences using TBLASTN with an e-value cutoff of 10 to predict the *TIFY* sequences in wheat^42,43^. The redundant sequences were removed using CD-HIT (http://weizhongli-lab.org/cd-hit/) and the remaining sequences displaying complete ORF were further examined for TIFY domain using the Hidden Markov Model (HMM) of Simple Modular Architecture Research Tool (SMART; http://smart.embl-heidelberg.de/) and Pfam (http://pfam.sanger.ac.uk/). A pipeline designed is shown in Supplementary Figure S1.

### Phylogenetic analysis

To elucidate the phylogenetic relationships of TIFY family of wheat with those of rice and *Arabidopsis*, we performed sequence based phylogenetic analysis using Clustal X 2.0^44^ and CLC Genomics Workbench 11.0 (https://www.qiagenbioinformatics.com/) at default settings. Another phylogenetic tree based on representative TIFY domains was constructed using MEGA 7.0 and Neighbor Joining method^45^. Bootstrap values were calculated from 1000 iterations.

### Characterization of identified novel *TIFY* genes in wheat

The physico-chemical characterization of the identified novel *TIFY* genes were carried out using ProtParam tool (http://web.expasy.org/protparam/). The gene structure of the identified TFs was studied using two HMM-based most accurate gene prediction tools: FGENESH (http://linux1.softberry.com/) and GenSCan (http://genes.mit.edu/GenSCan.html). N- and O-glycosylation potential sites were predicted by NetNGlyc 1.0 and NetOGlyc 1.0 Server (http://www.cbs.dtu.dk) respectively. Different catalytic domains and Nuclear localization signals (NLS) were determined using Motif Scan (https://www.ncbi.nlm.nih.gov/Structure/cdd/cdd.shtml) and NLStradamus software (http://www.moseslab.csb.utoronto.ca/NLStradamus/) respectively.

To identify presence of additional conserved motifs, Multiple Expectation Maximization for Motif Elicitation (MEME) version 5.1.1 was used (http://meme-suite.org/). The parameters used were minimum width 3, maximum width 50 and the maximum number of motifs to identify any number of repetitions. The subcellular localization and secondary structures of these sequences were predicted by WoLF PSORT (http://wolfpsort.org/) and PSIPRED Protein Analysis Workbench (http://bioinf.cs.ucl.ac.uk/) respectively. Transmembrane domains and pore lining helices were identified using MEMSAT-SVM tool at PSIPRED that utilizes support vector machine algorithm.

*Cis*-regulatory elements present in the 2.0 Kb upstream regions of all the identified *TaTIFY* genes were investigated using PLACE database (http://www.dna.affrc.go.jp/htdocs/PLACE/). Further, all identified *TIFY* genes were searched as target genes for conserved and novel wheat microRNAs available at miRBase, release 21 with psRNATarget tool (http://plantgm.noble.org/psRNATarget/) using an E-value cut off 3 for filtering microRNAs because low E-value specifies high resemblance between small RNAs and target genes.

### Tertiary structure prediction and validation

Tertiary structures were predicted at I-TASSER^46^ server based on ab initio methods for structure prediction. Five different models for each TaTIFY protein were generated and the model showing overall best stereo-chemical quality was selected for further assessment. The SAVES (http://services.mbi.ucla.edu/SAVES/) server was used to examine stereo-chemical properties of protein structure and ProSA-web server (http://prosa.services.came.sbg.ac.at/prosa.php) to calculate overall quality score to select the most reliable model. The 3D models were subjected to PyMOL Molecular Graphics System (http://pymol.org/ep) to obtain the final structures. The PDB files of the modeled TaTIFY proteins were subjected to PDBsum server (http://www.ebi.ac.uk/thorntonsrv/databases/pdbsum/Generate.html) for structural motif analysis and to ProSA-web server for obtaining the reliable values for the model generated. The final models were subjected to DoGSiteScorer (http://dogsite.zbh.uni-hamburg.de/calcPockets.php) to identify the probable binding sites.

### Gene ontology and enrichment analysis

To assign putative functions to the identified *TaTIFY* genes, Blast2GO^47^ program was used to BLAST against annot8r databases that stores UniProt entries with their associated Gene Ontologies (GO), Enzyme Commission (EC) and Kyoto Encyclopedia of Genes and Genomes (KEGG) annotations (http://www.nematodes.org/bioinformatics/annot8r/). The Web Gene Ontology Annotation Plot (WEGO; http://wego.genomics.org.cn) online tool was used to perform GO enrichment analysis of identified *TaTIFY* genes.

### Chromosomal localization and synteny analysis

Each identified *TaTIFY* gene was mapped on wheat chromosomes using BLAST tool at UR INRA "Génomique Info" (URGI, https://urgi.versailles.inra.fr/), database selecting all wheat chromosomes with an e-value cutoff of 0.001 and identity > 80%. The identified *TaTIFY* genes were also mapped to rice, sorghum, maize, barley and *Brachypodium* genomes using Ensembl Plant Database by selecting e-value 0.001 as cutoff criteria^48^. Multiple genome comparison between the selected pairs of chromosomal regions containing *TaTIFY* genes was performed for synteny analysis using identified orthologous and paralogous genes of the selected species. The syntenic relationships were visualized using the online Circos tool^49^.

### Molecular cloning, sequencing, and sequence characterization

Genomic DNA of wheat cultivar HD2329 + *Lr28* was isolated using DNeasy Plant Mini Kit (Qiagen). DNA amplifications were conducted in 20 µL volumes containing 100 ng of genomic DNA and 10 pM of each primer (sequences provided in Supplementary Table S19). The amplified products were cloned and plasmids from five independent clones, obtained with each primer set, were sequenced from both ends commercially. Total RNA was extracted using TriReagent (Sigma-Aldrich) and 5 µg was reverse-transcribed to cDNA using Transcriptor First Strand cDNA Synthesis Kit (Roche Diagnostic, Indianapolis, USA). PCR, cloning and sequencing was performed as mentioned earlier and each consensus sequence was analyzed^50,51^. The nucleotide and deduced amino acid sequences were characterized using the same bioinformatics tools as mentioned earlier.

### Response of TIFY TF to biotic and abiotic stresses

#### Plant and pathogen material, stress treatment of plants and quantitative Real Time PCR

Wheat near-isogenic lines (NILs) HD2329 (seedling leaf rust susceptible, infection type 3+) and HD2329 + *Lr28* (seedling leaf rust resistant, nest immune, infection type 0–0) were used. The *Lr28* gene, effective against all pathotypes of the pathogen in India, was derived from *Aegilops speltoides* (Teusch) and introgressed on the long arm of chromosome 4AL^52^. *Puccinia triticina* pathotype 77–5, the most predominant and devastating pathotype in all parts of the Indian subcontinent, was selected as experimental pathogen. Experiments were conducted at the National Phytotron Facility, IARI, New Delhi and seedlings of the isogenic lines were inoculated with either urediniospores of race 77–5 mixed with talc (1:1) or mock inoculated with talc. Leaf samples were collected at different time points for RNA isolation: 0 h (i.e. just before inoculation) and at 6, 12, 24, 48, 72- and 168-h post inoculation (hpi) from five independently treated susceptible and resistant NILs. The collected samples (three leaves from each plant) were immediately frozen in liquid Nitrogen and used for RNA isolation. Susceptible mock inoculated was named S-M, Susceptible Pathogen inoculated as S-PI, Resistant mock inoculated R-M and Resistant Pathogen inoculated as R-PI.

The wheat cultivar Chinese Spring was used for the abiotic stress study. Four different phytohormones [SA, ABA, Methyl Jasmonate (MJ) and Jasmonic Acid (JA)] at concentration of 5 mM were used for abiotic stress treatments. The samples were divided in two groups: control: 4 plants and treated: 7 plants for each treatment. Treatments were performed in duplicates. Seeds were germinated on autoclaved composite soil containing peat, sand and soil (2:1:1 ratio), grown to three leaf stage (~ 14 days after germination) at the Green House facility, BIT, Mesra, Ranchi under ideal conditions (temperature 20 °C, relative humidity 80%, 14 h light at 100 µmol m^−2^ s^−1^ and 10 h of darkness). The pots were watered well regularly. The test seedlings were sprayed with respective phytohormones, while control plants were sprayed with only milli-Q water having no phytohormone. Leaf samples for RNA isolation were collected at 0, 1, 2, 4, 8, 12 and 24 h post phytohormone application. The collected samples (five leaves from each set) were immediately used for RNA isolation.

Primers used for qRT-PCR are mentioned in Supplementary Table S21. Samples were run in two biological and three technical replicates, and the experiment was repeated once again to check reproducibility. Wheat Glyceraldehyde-3-Phosphate Dehydrogenase (GAPDH; GenBank Accession No. AF521191), was chosen as the endogenous control for normalization of input RNA differences and examine efficiencies of reverse transcription among the samples^53^ and gene expression levels were computed using the 2^−∆∆Ct^ method^54^.

### Statistical analysis

The qRT-PCR data were statistically analyzed using Wilcoxon signed rank sum test to compare expression data from infected and mock-inoculated materials while, Spearman's correlation was used to compare expression data from phytohormone treated and control plants.

## Supplementary Information

Supplementary Information

## Data Availability

All the processed sequences were deposited in the National Center for Biotechnology Information (NCBI) and can be accessed as mentioned in Table 2.
