# Supplementary material for: Comprehensive molecular dissection of TIFY Transcription factors reveal their dynamic responses to biotic and abiotic stress in wheat (Triticum aestivum L.)
Source: Sci Rep. 2021 May 6;11:9739. doi: 10.1038/s41598-021-87722-w (PMC8102568; doi:10.1038/s41598-021-87722-w)
Supplement: Supplementary file 1 — Supplementary Information [file 41598_2021_87722_MOESM1_ESM.pdf]

Comprehensive molecular dissection of TIFY transcription factor gene family in wheat (*Triticum aestivum* L.)

Singh Poonam and Mukhopadhyay Kunal[*]

Department of Bio-Engineering, Birla Institute of Technology, Mesra, Ranchi-835215 Jharkhand, India.

[*] Corresponding author E-mail ID: kmukhopadhyay@bitmesra.ac.in

Phone: 919431382720

Fax: 916512275401

Supplementary Table 1 List of sequences used to construct phylogenetic tree.

Sl. No.	Plant name	Sequence name in phylogenetic tree	GenBank Accession ID
1	*Arabidopsis thaliana*	TIFY10B_AT1g74950	AT1g74950
2	*Arabidopsis thaliana*	TIFY11A_AT1g17380	AT1g17380
3	*Arabidopsis thaliana*	TIFY10B_AT1g72450	AT1g72450
4	*Arabidopsis thaliana*	TIFY10A_AT1g19180	AT1g19180
5	*Arabidopsis thaliana*	TIFY9_AT5g13220	AT5g13220
6	*Arabidopsis thaliana*	TIFY8_AT4g32570	AT4g32570
7	*Arabidopsis thaliana*	TIFY7At1g70700	At1g70700
8	*Arabidopsis thaliana*	TIFY6B_AT3g17860	AT3g17860
9	*Arabidopsis thaliana*	TIFY6A_AT1g48500	AT1g48500
10	*Arabidopsis thaliana*	TIFY5B_AT2g34600	AT2g34600
11	*Arabidopsis thaliana*	TIFY5A_AT1g30135	AT1g30135
12	*Arabidopsis thaliana*	TIFY4B_AT4g14720	AT4g14720
13	*Arabidopsis thaliana*	TIFY4A_AT4g14713	AT4g14713
14	*Arabidopsis thaliana*	TIFY3B_AT5g20900	AT5g20900
15	*Arabidopsis thaliana*	TIFY3A_AT3g43440	AT3g43440
16	*Arabidopsis thaliana*	TIFY2B_AT3g21175	AT3g21175
17	*Arabidopsis thaliana*	TIFY2A_AT1g51600	AT1g51600
18	*Arabidopsis thaliana*	TIFY1_AT4g24470	AT4g24470

19	*Sorghum bicolor*	Sb01g023431 TIFY11E	Sb01g023431
20	*Sorghum bicolor*	Sb01g027325 TIFY11B	Sb01g027325
21	*Sorghum bicolor*	Sb02g003130 TIFY5	Sb02g003130
22	*Sorghum bicolor*	Sb01g045180 TIFY11C	Sb01g045180
23	*Sorghum bicolor*	Sb01g045190 TIFY11A	Sb01g045190
24	*Sorghum bicolor*	Sb03g044485	Sb03g044485
25	*Oryza sativa*	OsTIFY1A	Os03g47970.1
26	*Oryza sativa*	OsTIFY1B	Os03g52450.1
27	*Oryza sativa*	OsTIFY2A	Os02g05510.3
28	*Oryza sativa*	OsTIFY2B	Os06g48534.1
29	*Oryza sativa*	OsTIFY2B	Os06g48534.1
30	*Oryza sativa*	OsTIFY3	Os04g55920.2
31	*Oryza sativa*	OsTIFY5	Os07g05830.1
32	*Oryza sativa*	OsTIFY6A	Os08g33160.1
33	*Oryza sativa*	OsTIFY6B	Os09g23660.1
34	*Oryza sativa*	OsTIFY8	Os02g49970.1
35	*Oryza sativa*	OsTIFY9	Os04g32480.1
36	*Oryza sativa*	OsTIFY10A	Os03g28940.1
37	*Oryza sativa*	OsTIFY10B	Os07g42370.2
38	*Oryza sativa*	OsTIFY10C	Os09g26780.1
39	*Oryza sativa*	OSTIFY11A	Os03g08310.1

40	*Oryza sativa*	OsTIFY11B	Os03g08330.1
41	*Oryza sativa*	OsTIFY11C	Os03g08320.1
42	*Oryza sativa*	OsTIFY11D	Os10g25290.1
43	*Oryza sativa*	OsTIFY11E	Os10g25230.1
44	*Oryza sativa*	OsTIFY11F	Os10g25250.1
45	*Oryza sativa*	OsTIFY11G	Os03g27900.1

Supplementary Figure S1. Pipeline representing the strategies used for identification novel TIFY transcription factors in wheat.

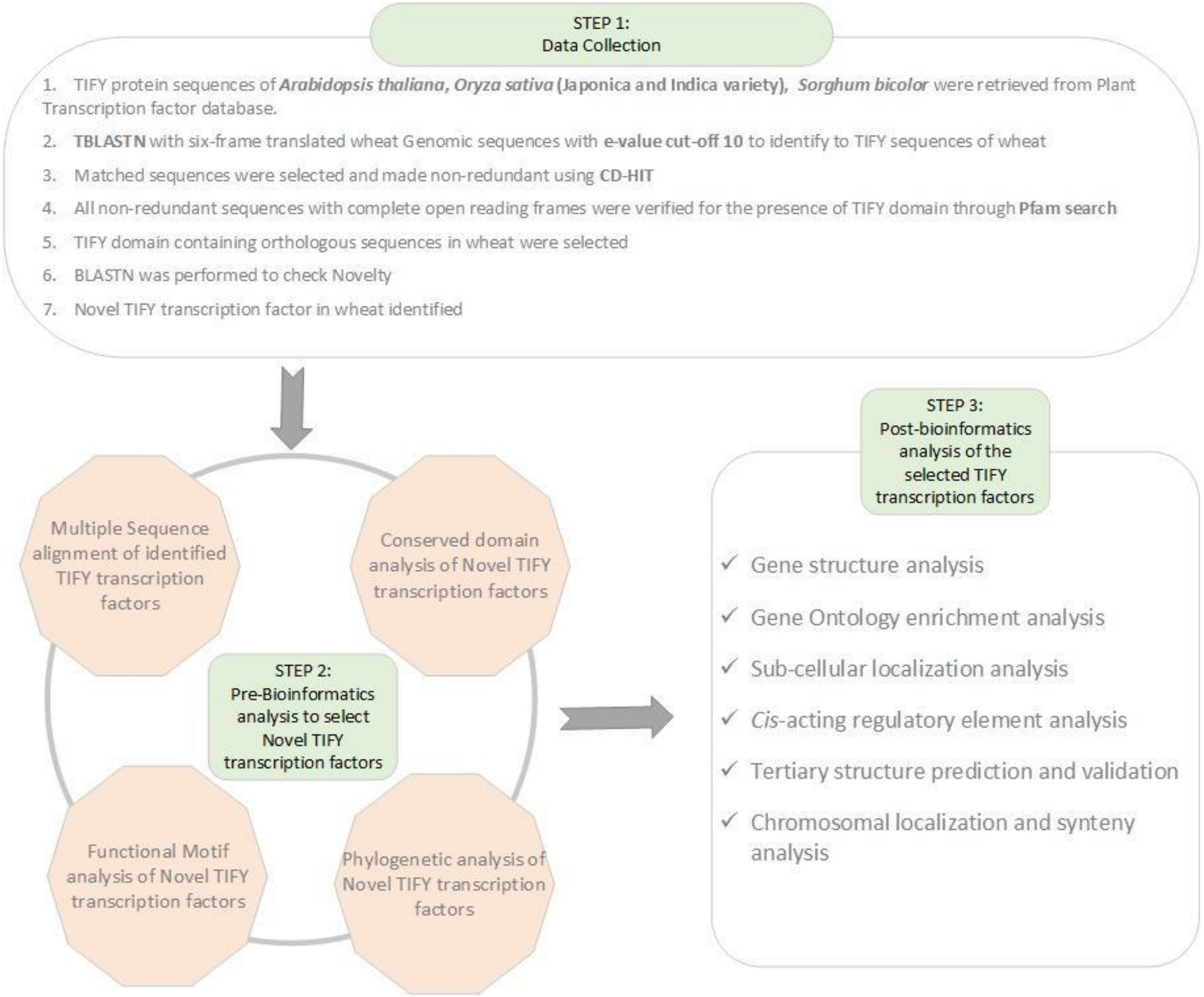

Supplementary Figure S2. Multiple sequence alignment of identified 23 TaTIFY protein sequences showing QLTIFYGGR, PY, RKASL and KRKDR domains.

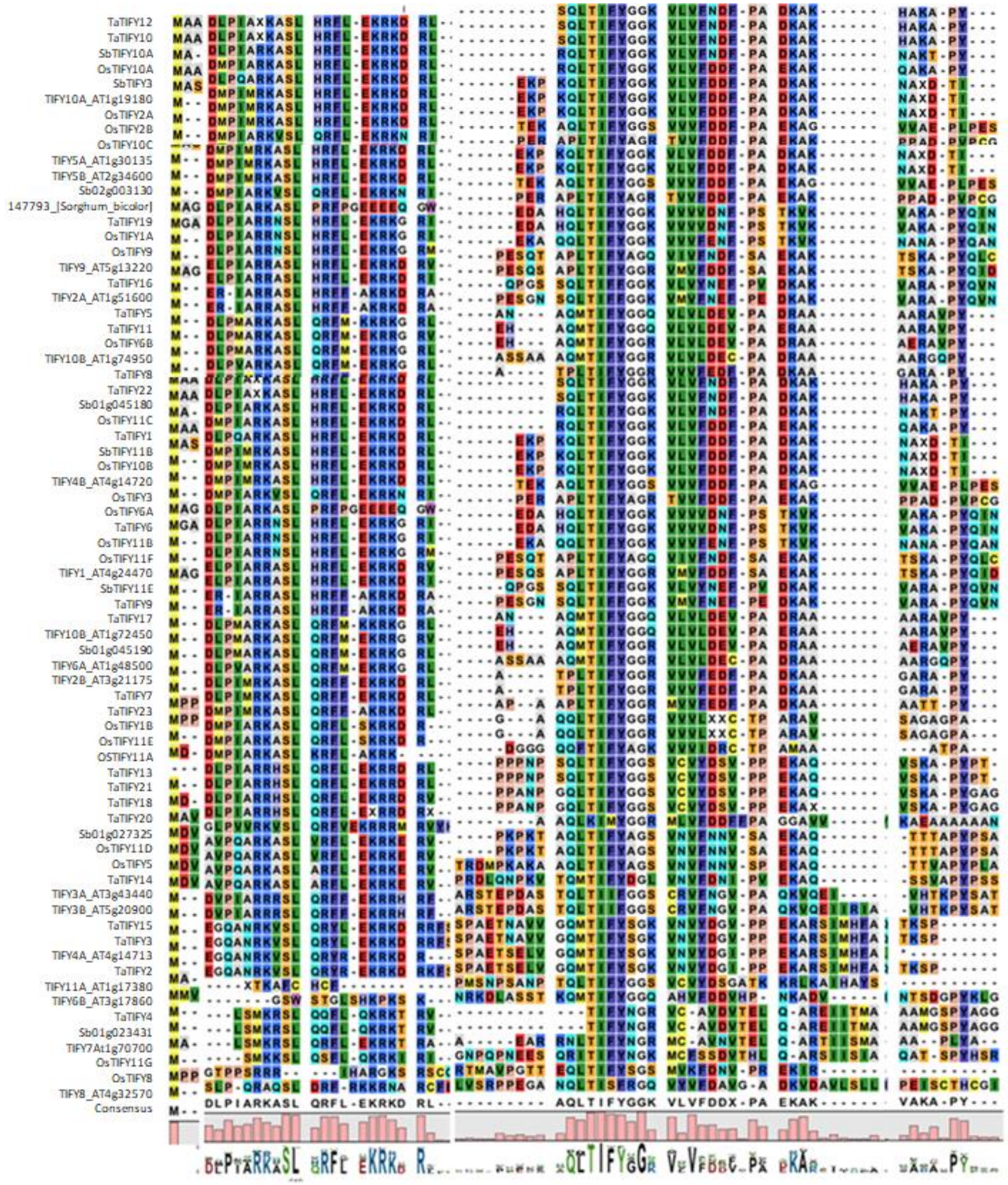

Supplementary Table S2 *cis*-elements analysis for *TaTIFY* genes.

Sl. No.	***Motifs found***	***Function***
1.	ARE	cis-acting regulatory element essential for the anaerobic induction
2.	CAAT-box	cis-acting element in promoter and enhancer regions
3.	CATT-motif	cis-acting regulatory element involved in the MeJA-responsiveness
4.	GAG-motif	part of a light responsive element
5.	GCN4_motif	cis-regulatory element involved in endosperm expression
6.	HSE	cis-acting element involved in heat stress responsiveness
7.	I-box	part of a light responsive element
8.	MBS	MYB binding site involved in drought-inducibility
9.	O2-site	cis-acting regulatory element involved in zein metabolism regulation
10.	Skn-1_motif	cis-acting regulatory element required for endosperm expression
11.	Sp1	Light Responsive Element
12.	TATA-box	core promoter element around -30 of transcription start
13.	TATC-box	cis-acting element involved in gibberellin-responsiveness
14.	TGACG-motif	cis-acting regulatory element involved in the MeJA-responsiveness
15.	Circadian	cis-acting regulatory element involved in circadian control
16.	motif IIb	abscisic acid responsive element
17.	ABRE	Dehydration responsive element
18.	CAT-box	cis-acting regulatory element related to meristem expression
19.	CGTCA-motif	cis-acting regulatory element involved in the MeJA-responsiveness
20.	EIRE	elicitor-responsive element
21.	G-box	cis-acting regulatory element involved in light responsiveness
22.	GT1-motif	light responsive element
23.	MNF1	light responsive element

24.	TGA-element	auxin-responsive element
25.	ACE	cis-acting element involved in light responsiveness
26.	Box-W1	fungal elicitor responsive element
27.	C-repeat/DRE	regulatory element involved in cold- and dehydration-responsiveness
28.	CCGTCC-box	cis-acting regulatory element related to meristem specific activation
29.	GC-motif	enhancer-like element involved in anoxic specific inducibility
30.	chs-CMA2a	part of a light responsive element
31.	rbcS-CMA7a	part of a light responsive element
32.	AE-box	part of a module for light response
33.	TC-rich repeats	cis-acting element involved in defense and stress responsiveness
34.	TCA-element	cis-acting element involved in salicylic acid responsiveness
35.	5UTR Py-rich stretch	cis-acting element conferring high transcription levels
36.	GARE-motif	gibberellin-responsive element
37.	LTR	cis-acting element involved in low-temperature responsiveness
38.	RY-element	cis-acting regulatory element involved in seed-specific regulation
39.	as-2-box	involved in shoot-specific expression and light responsiveness
40.	P-box	gibberellin-responsive element

Supplementary Table S3 Wheat miRNAs targeting wheat TIFY transcripts.

SL. No.	miRNA_Acc.	Target Accession ID	Target_TIFY Gene	Target region	Inhibition	Multiplicity
1.	tae-miR9780	2DS_5359716	TaTIFY1	335-355	Translation	1
2.	tae-miR9677a	2BL_10845579	TaTIFY2	189-212	Cleavage	1
3.	tae-miR5384-3p	4DL_14413893	TaTIFY3	614-636	Cleavage	1
4.	tae-miR408	4DL_14373184	TaTIFY4	294-312	Cleavage	1
5.	tae-miR1134	4DL_14351878	TaTIFY5	660-680	Cleavage	1
6.	tae-miR9780	2DS_5348573	TaTIFY6	571-591	Cleavage	1
7.	tae-miR1138	5BL_10857778	TaTIFY7	514-534	Cleavage	1
8.	tae-miR9678-3p	5BL_10845579	TaTIFY8	517-538	Cleavage	1
9.	tae-miR5384-3p	5BL_10896624	TaTIFY9	499-519	Cleavage	1
10.	tae-miR1136	2BL_7870876	TaTIFY10	511-532	Cleavage	1
11.	tae-miR399	4AS_5934783	TaTIFY11	322-345	Translation	1
12.	tae-miR9657a-3p	4DL_14448131	TaTIFY12	398-421	Cleavage	1
13.	tae-miR1136	2BL_7870876	TaTIFY13	410-430	Cleavage	1
14.	tae-miR9677a	2AS_5157670	TaTIFY14	1099-1119	Translation	1
15.	tae-miR9653a-3p	7DS_2913568	TaTIFY15	503-523	Cleavage	1
16.	tae-miR9669-5p	2BL_7975941	TaTIFY16	828-848	Cleavage	1
17.	tae-miR9780	5BL_339996	TaTIFY17	482-502	Translation	1
18.	tae-miR1134	2DS_5344665	TaTIFY18	465-485	Cleavage	1
19.	tae-miR171b	2BL_7870876	TaTIFY19	113-133	Cleavage	1

20.	tae-miR9678-3p	2DS_5344665	TaTIFY20	46-66	Cleavage	1
21.	tae-miR9657a-3p	2DS_93551	TaTIFY21	131-152	Cleavage	1
22.	tae-miR9670-3p	5DL_4513175	TaTIFY22	226-246	Cleavage	1
23.	tae-miR531	2AS_3183635	TaTIFY23	220-240	Cleavage	1

Multiplicity indicates that miRNA have multiple target sites on a specific target transcript. This increases miRNA target activity to the mRNA target.

Supplementary Table S4 Physico-chemical characteristics of identified wheat TIFY TF proteins.

Name of TF	No. of amino acids	Molecular weight (Da)	Theoretical pI	Extinction coefficient	Instability Index	Aliphatic Index	GRAVY	No. of glycosylation sites	
								N-	O-
TaTIFY1	231	24279.2	8.57	0.411	34.12	67.88	-0.473	4	15
TaTIFY2	113	12034.6	10.84	0.715	84.35	67.52	-0.386	2	8
TaTIFY3	189	19631.5	9.48	0.788	56.59	76.24	-0.017	-	4
TaTIFY4	208	22029.0	8.52	1.140	64.00	60.19	-0.470	-	5
TaTIFY5	163	16969.8	9.39	0.770	77.26	69.69	-0.181	4	14
TaTIFY6	231	24279.2	8.57	0.411	34.12	67.88	-0.473	1	15
TaTIFY7	105	11293.9	8.97	0.264	48.40	82.86	-0.310	3	3
TaTIFY8	235	24792.1	10.01	0.125	73.77	64.47	-0.366	3	56
TaTIFY9	186	19727.2	6.30	0.430	48.30	78.71	-0.470	5	14
TaTIFY10	151	15452.7	9.64	0.297	56.10	76.49	-0.094	3	17
TaTIFY11	163	16969.8	9.39	0.770	77.26	69.69	-0.181	-	14
TaTIFY12	189	19416.2	9.10	0.513	57.42	80.42	0.083	-	13
TaTIFY13	206	21643.4	9.48	0.466	53.18	69.85	-0.392	4	19
TaTIFY14	216	22770.7	8.91	0.438	38.20	72.08	-0.487	4	18
TaTIFY15	147	15275.6	9.45	0.203	44.23	83.27	-0.014	1	7
TaTIFY16	113	12034.6	10.84	0.715	84.35	67.52	-0.386	2	8
TaTIFY17	186	19727.2	6.30	0.430	48.30	78.71	-0.470	5	14
TaTIFY18	160	17574.8	9.37	0.092	41.23	72.69	-0.394	3	10
TaTIFY19	57	6066.2	8.86	0.799	57.01	53.16	0.095	2	2

TaTIFY20	160	17574.8	9.37	1.376	41.23	72.69	-0.394	3	10
TaTIFY21	162	18574.8	10.37	0.092	41.23	89.89	-0.394	3	12
TaTIFY22	235	24792.1	10.01	0.125	73.77	64.47	-0.366	3	50
TaTIFY23	140	14726.0	9.29	0.211	57.54	83.21	-0.029	-	4

Supplementary Table S5 Different catalytic motifs present in identified novel TaTIFY proteins.

NAME OF TF	CATALYTIC DOMAIN																
	ASN_GLYCOSYLATION	CK2_PHOSPHO_SITE	MYRISTYL	PKC_PHOSPHO_PHO_SITE	TIFY	AMIDATION	CAMP_PHOSPHO_SITE	CCT	TYR_PHOSPHO_SITE	CCT2	OCTAPEPTIDE	NLS_BP	FARP	DUF2149	ALA_RICH	CheC	APNUCLEASE
TaTIFY1	+	+	+	+	+	+	+	–	–	+	–	–	–	–	–	–	–
TaTIFY2	–	–	+	+	+	+	+	–	–	+	–	–	–	–	–	–	–
TaTIFY3	–	–	+	–	+	–	+	–	–	+	–	–	–	–	+	–	–
TaTIFY4	–	+	+	+	+	–	+	–	–	+	+	–	–	–	–	–	–
TaTIFY5	–	+	–	+	+	–	+	+	+	+	–	+	–	–	–	–	–
TaTIFY6	+	+	+	+	+	+	+	–	–	+	–	–	–	–	–	–	–
TaTIFY7	+	+	+	+	+	–	+	+	–	+	–	+	+	–	–	–	–
TaTIFY8	–	+	+	+	+	–	+	+	–	+	–	–	–	–	–	+	+
TaTIFY9	+	+	+	–	+	–	+	–	–	+	–	+	–	–	+	–	–
TaTIFY10	–	+	–	+	+	–	+	+	+	+	–	+	–	–	–	–	–
TaTIFY11	–	–	+	+	+	–	+	+	–	+	–	+	–	–	+	–	–
TaTIFY12	+	+	+	+	+	–	+	–	–	+	–	–	–	+	+	–	–
TaTIFY13	+	+	+	+	+	+	+	+	–	+	–	+	–	–	–	–	–
TaTIFY14	–	+	+	+	+	–	+	–	–	+	–	+	–	–	–	–	–
TaTIFY15	–	–	+	+	+	+	+	–	–	+	–	–	–	–	–	–	–
TaTIFY16	–	+	+	+	+	–	+	+	–	+	–	+	–	–	–	+	+
TaTIFY17	+	+	–	+	+	–	+	+	–	+	+	+	–	–	–	–	–
TaTIFY18	–	–	+	+	+	–	–	–	–	–	–	–	–	–	–	–	–
TaTIFY19	–	–	+	+	+	–	–	–	–	–	–	–	–	–	–	–	–
TaTIFY20	+	+	–	+	+	–	+	+	+	+	+	+	–	–	–	–	–
TaTIFY21	+	+	+	+	+	+	+	+	+	+	+	+	–	–	–	–	–
TaTIFY22	+	+	+	+	+	–	+	+	–	+	–	+	+	–	–	–	–
TaTIFY23	–	+	+	+	+	–	+	+	–	+	–	+	–	–	–	–	–

Supplementary Table S6 Prediction of Nuclear Localization Signals in identified novel TaTIFY TFs.

Name	Nuclear Localisation Signal	
	**Bipartite**	**Monopartite**
TaTIFY1	RFLEKRKDRLHAKAPYQAPPSDATPAKKEFE	–
TaTIFY2	KRSLQQFLQKRKTRVAAMGSPYAGGR	–
TaTIFY3	TWKSKSRRFALACGVLSQYVKAEQKMSSV	–
TaTIFY4	RPGKPVAGGRPARPWLRGQPPRSQLKLKGI	–
TaTIFY5	–	LPKKKGKTEASSW
TaTIFY6	RFLEKRKDRLHAKAPYQAPPSDATPAKKEFE	–
TaTIFY7	RFMEKRKGRLAERAVPYSRPDGNAASCNRLTLT	–
TaTIFY8	KPKTAQLTIFYAGSVNVFNNVSAEKAQEL	–
TaTIFY9	RFLEKRKGRIVAKAPYQINSASAAPSKQANGD	–
TaTIFY10	–	–
TaTIFY11	–	LPKKKGKTEASSW
TaTIFY12	RFFEKRKDRLGARAPYARPAPAAAAIKDSEEK	–
TaTIFY13	RRDRAVSKAPYGAGKPSEGLAASSGMEAVAAGK	–
TaTIFY14	RFLEKRKDRLHAKAPYQASPSDATPVKKEF	–
TaTIFY15	RFMKKRKGRLAARAVPYSRPDGDAFSSNRLTL	–
TaTIFY16	KRSLQQFLQKRKTRVAAMGSPYAGGRR	–
TaTIFY17	RFLEKRKGRIVAKAPYQINSASAAPSKQANG	–
TaTIFY18	DKEKPKQLTIFYGGKVLVFDDFPADKAKDLM	–
TaTIFY19	KKRLKAIHAYSCGCSSCGATKAFCH	–
TaTIFY20	DKEKPKQLTIFYGGKVLVFDDFPADKAKDLM	–
TaTIFY21	DKEKPKQLTIFYGGKVLVFDDFPADKAKDLM	–
TaTIFY22	KPKTAQLTIFYAGSVNVFNNVSAEKAQE	
TaTIFY23	RFMEKRRGRVAARAVPYSRPDGDAFSCNRL	

Supplementary Table S7 Different conserved motifs present in the novel 23 TaTIFY TF protein sequences.

Motif No.	Sites	E-value	Amino acid sequence composition of motif	Width (amino acids)
1	21	1.5e-1189	KQLTIFYGGKVLVFDDFPADKAKDLMQLAS	30
2	22	2.7e-570	NASDLPIARKASLHRFLEKRKDRLHAKAPY	30
3	10	1.9e-516	QTWKSKSRRFALACGVLSQYVKAEQ	25
4	15	1.9e-155	KGSPVVQNVALPQPS	15
5	20	4.9e-126	QASPSDATPVKKEFENQPWLGLGPNAALK	29
6	11	7.2e-124	SNCSFRCRTXQEELRTSMARIXTECRGFS	29
7	20	1.6e-102	MELFPQSVGFSIKDAAAPPREEQGDKEKP	20
8	13	6.6e-099	MTIFYNGRVCAVDVTELQAREIITMASQQI	30
9	17	9.2e-067	NHEESLRLGRPRNISFSGESPSTKLHI	27
10	10	2.2e-066	ELGLGINKGE	10

Supplementary Figure S3. Signature motifs identified in the 23 TaTIFY protein sequences.

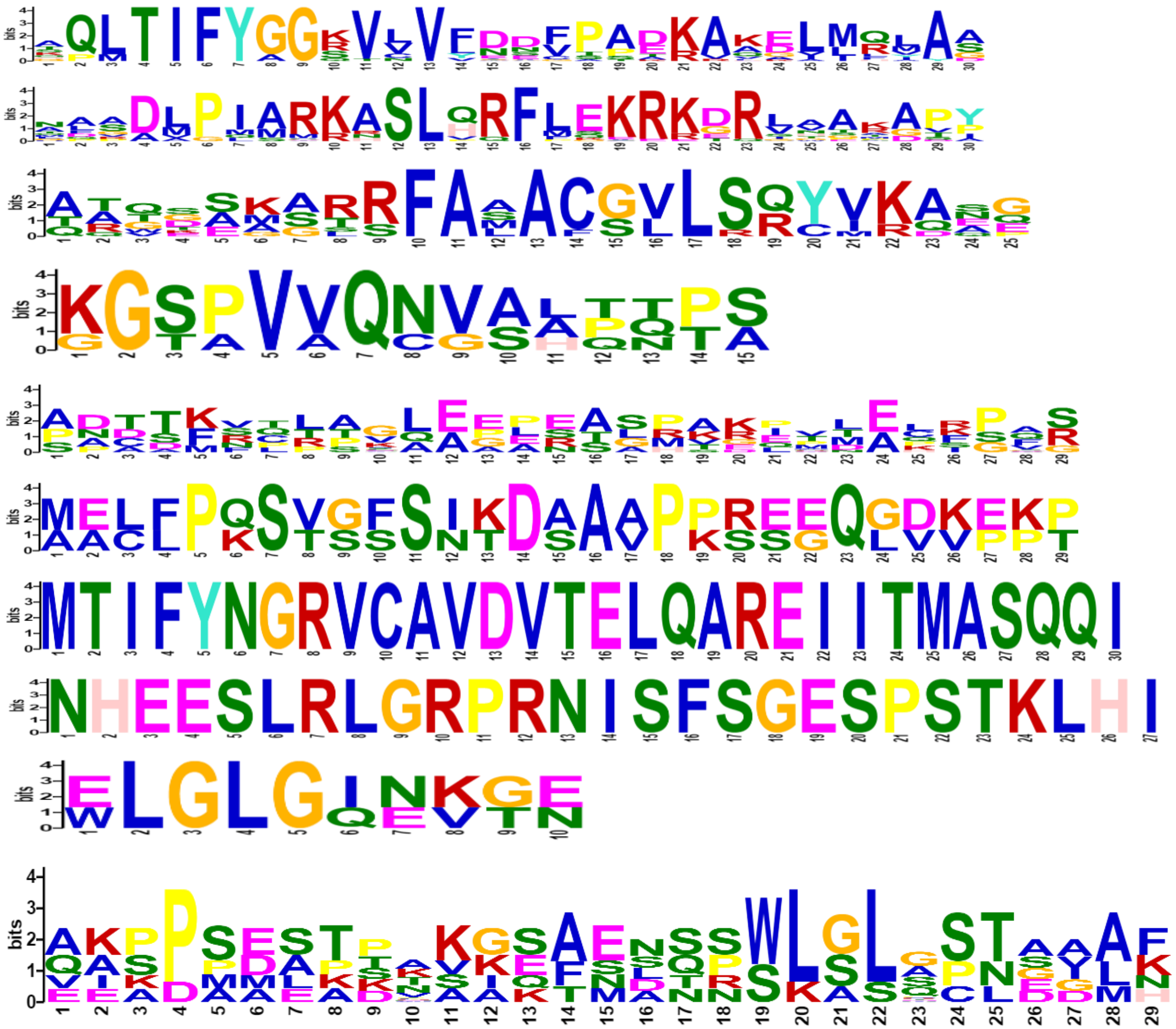

Supplementary Figure S4. Secondary structures of 23 novel TaTIFY TF proteins predicted using PSIPRED.

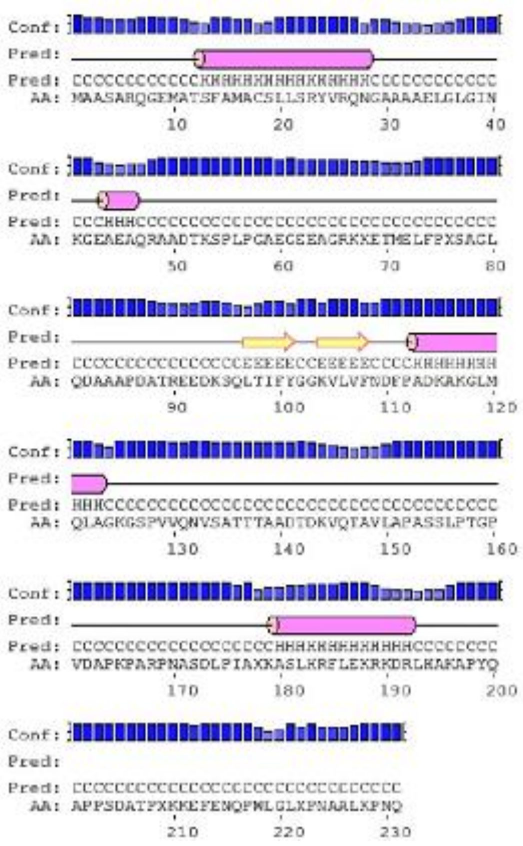

TaTIFY1

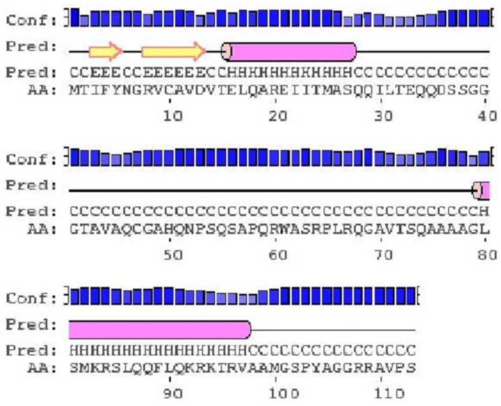

TaTIFY2

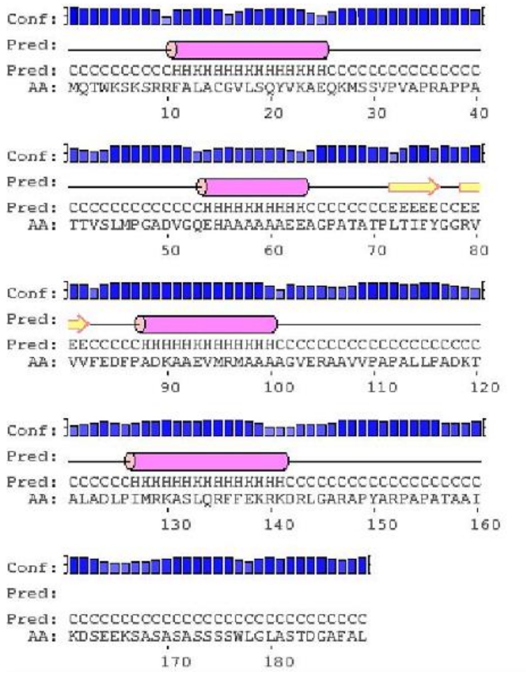

TaTIFY3

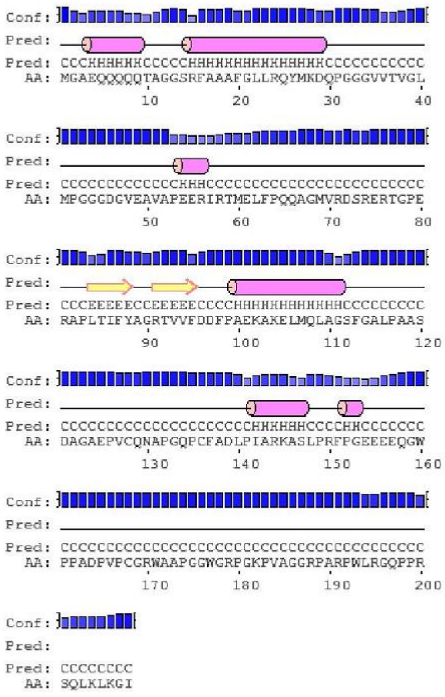

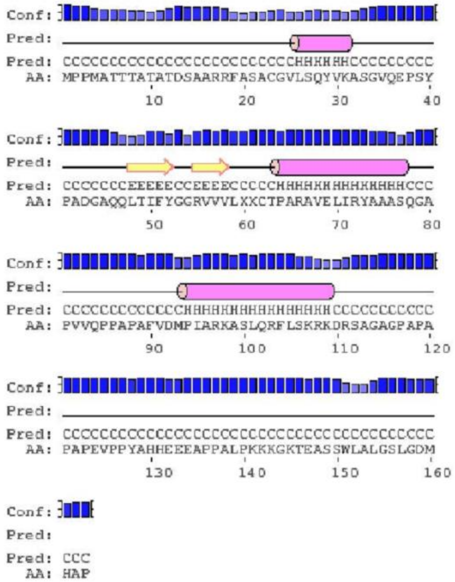

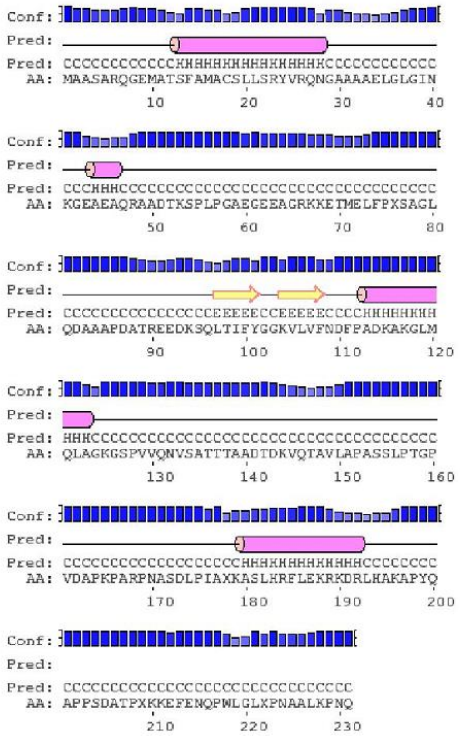

TaTIFY4 TaTIFY5 TaTIFY6

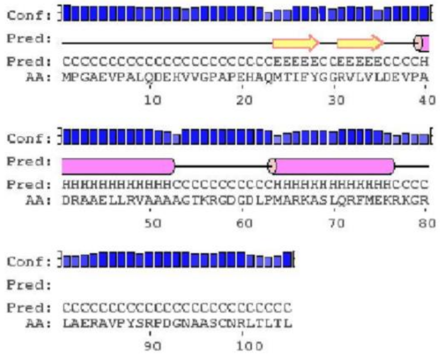

TaTIFY7

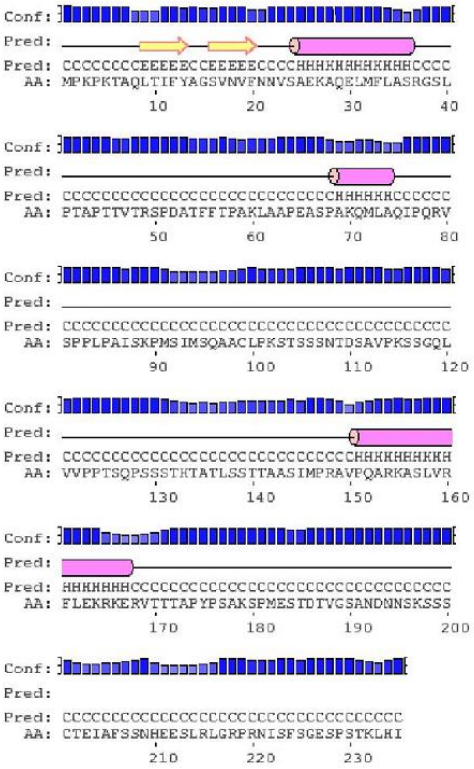

TaTIFY8

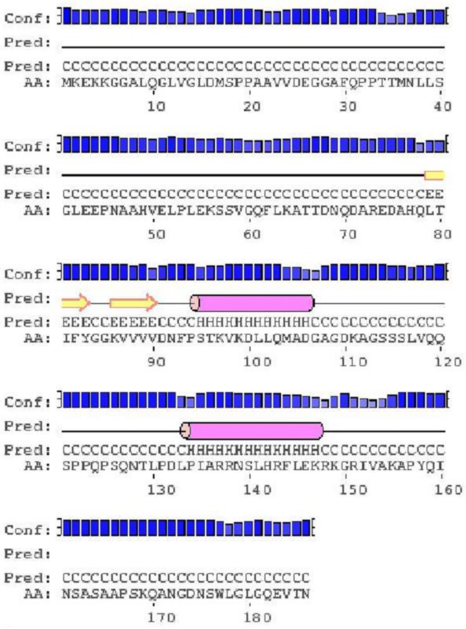

TaTIFY9

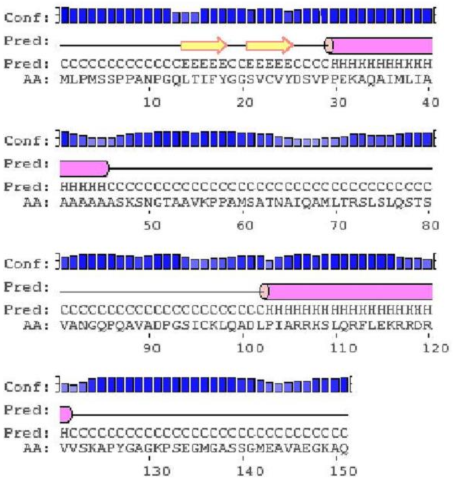

TaTIFY10

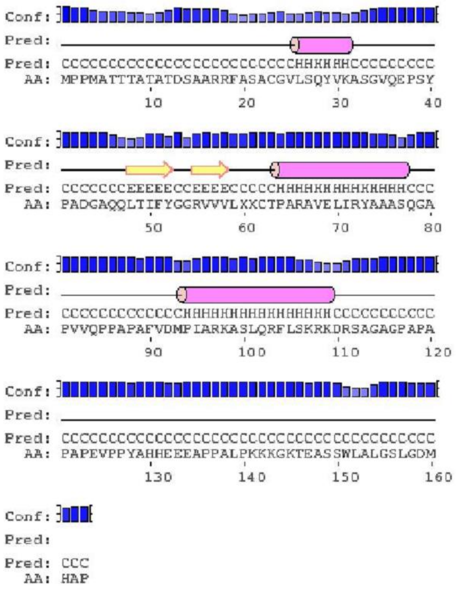

TaTIFY11

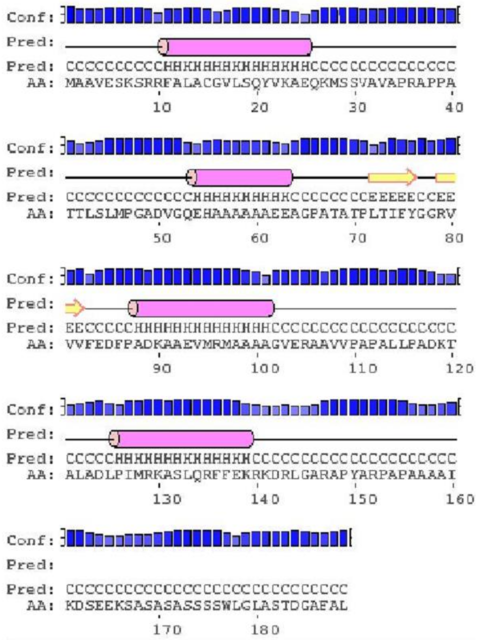

TaTIFY12

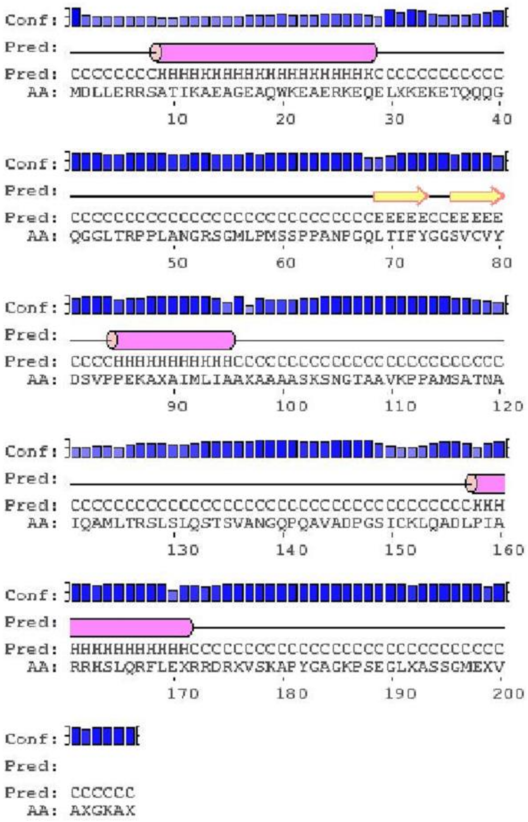

TaTIFY13

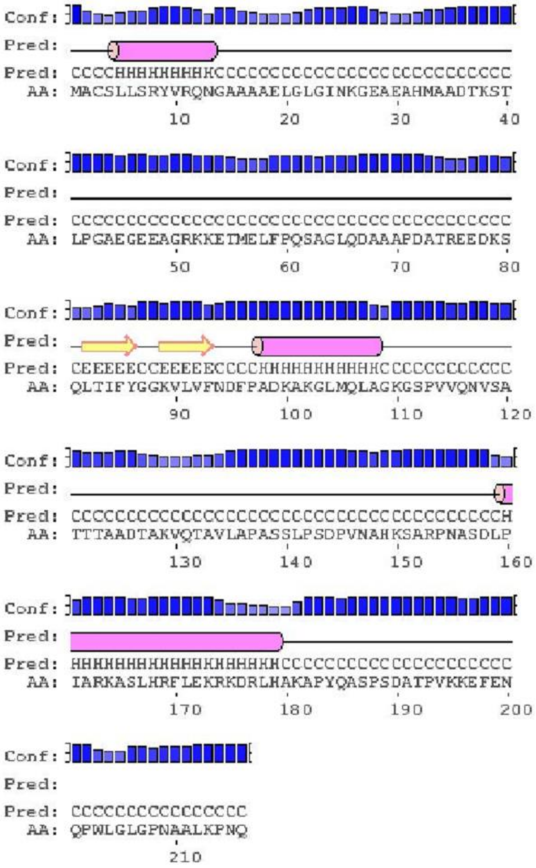

TaTIFY14

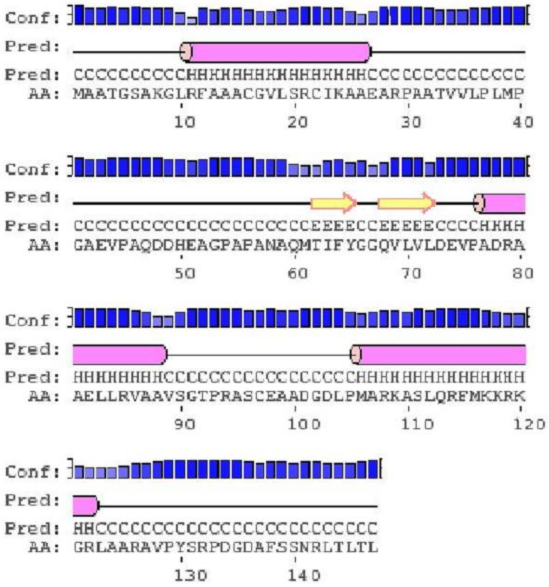

TaTIFY15

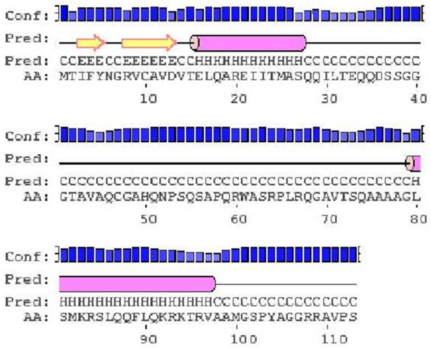

TaTIFY16

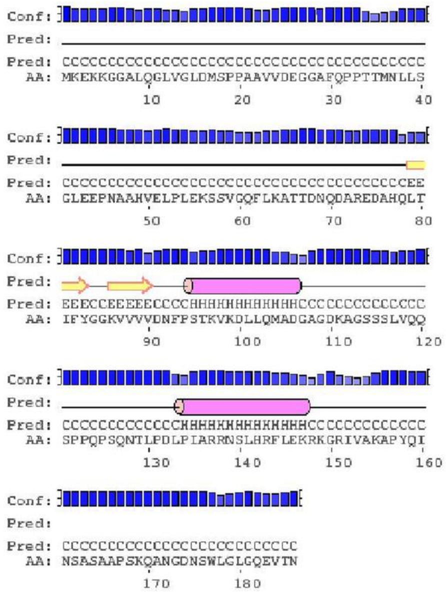

TaTIFY17

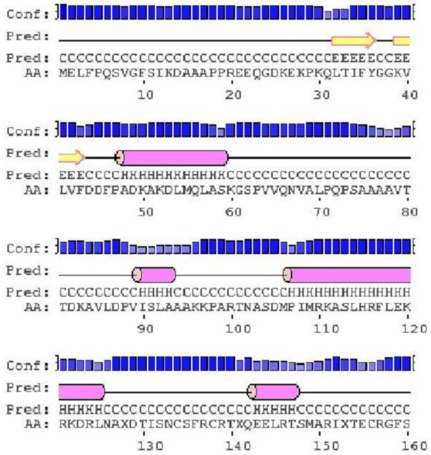

TaTIFY18

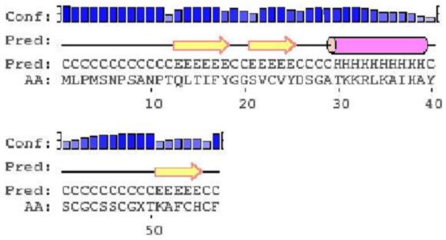

TaTIFY19

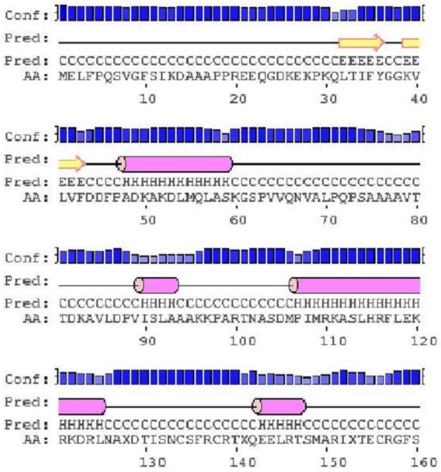

TaTIFY20

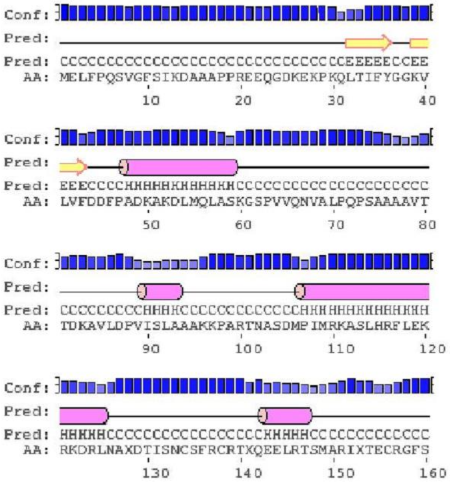

TaTIFY21

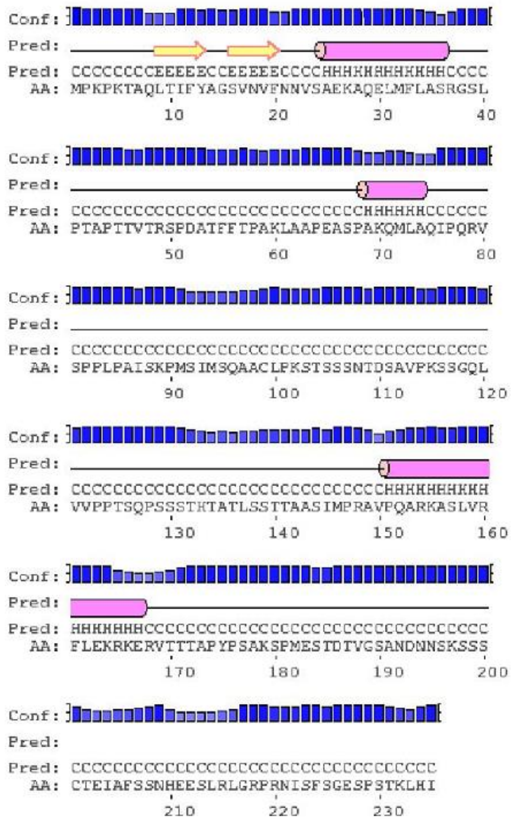

TaTIFY22

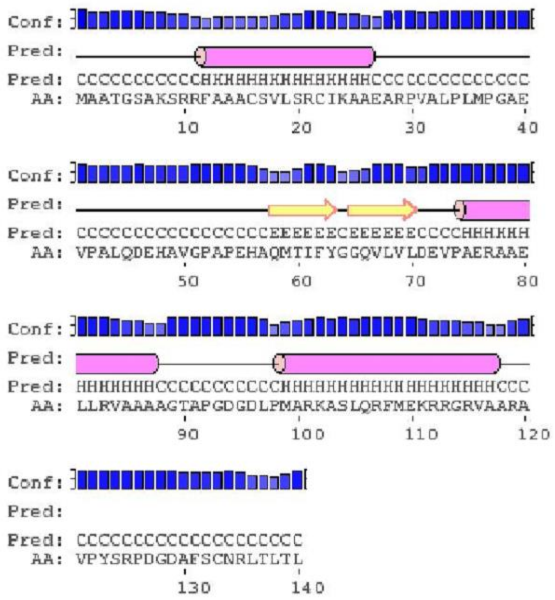

TaTIFY23

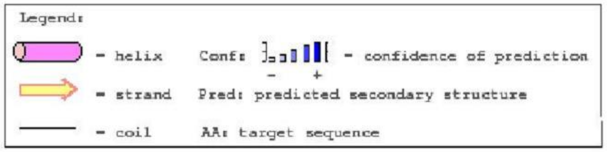

Supplementary Figure S5. Positions of pore lining helices present in the identified TaTIFY proteins.

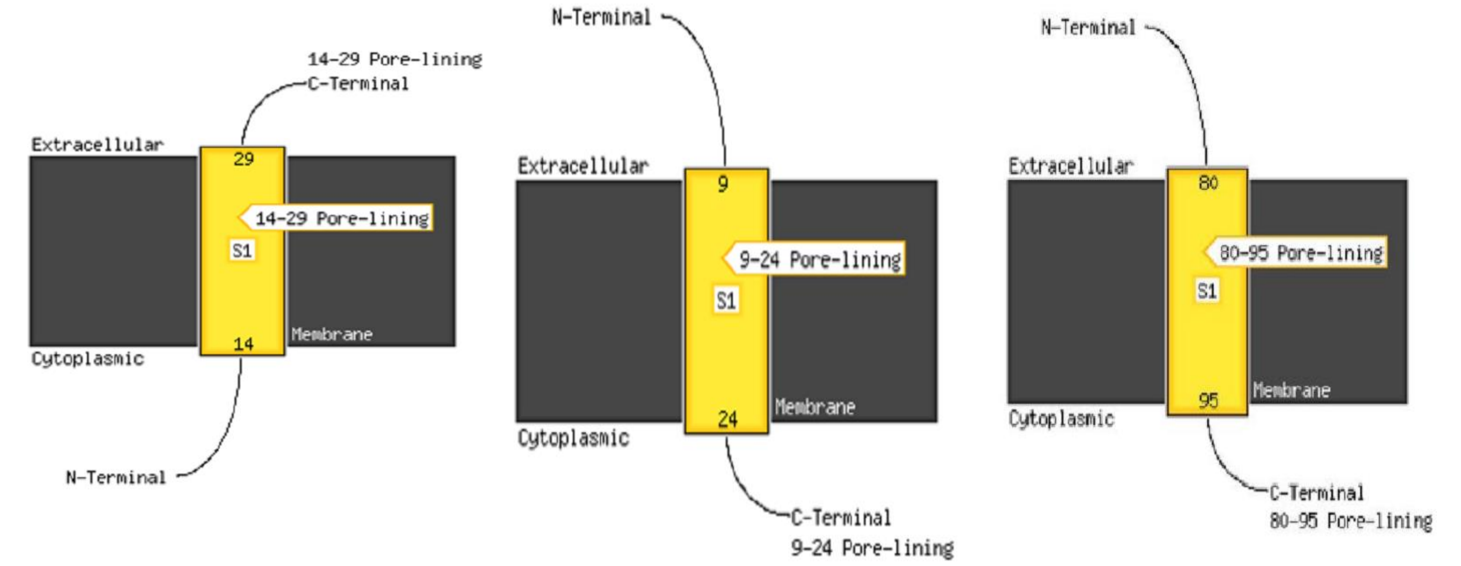

TaTIFY4 TaTIFY8 TaTIFY9

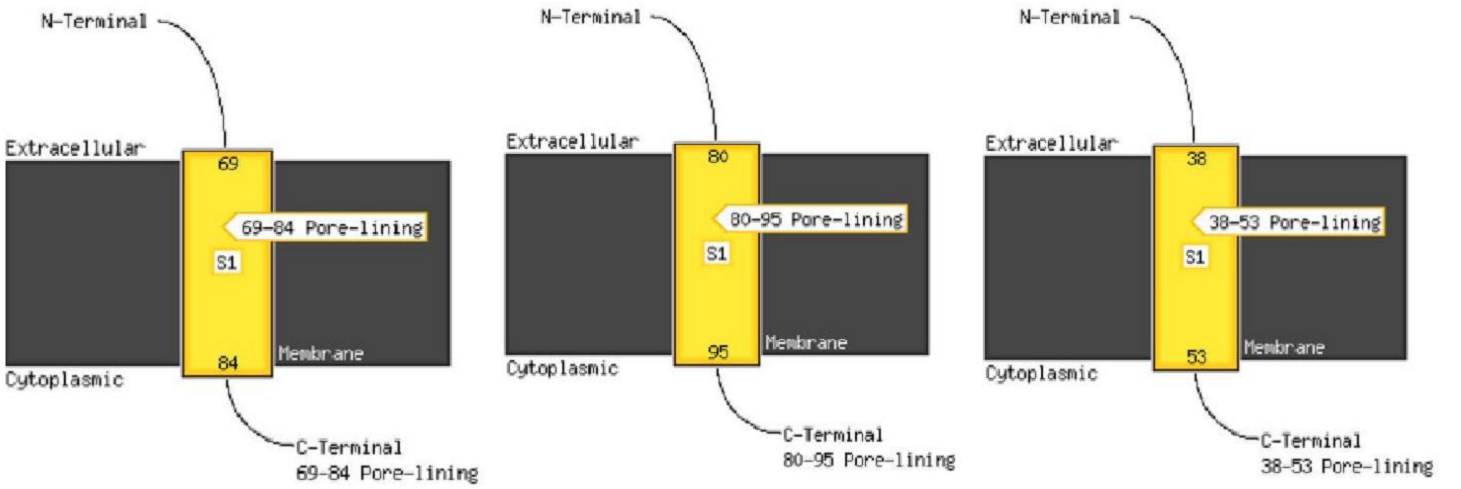

TaTIFY10 TaTIFY13 TaTIFY17

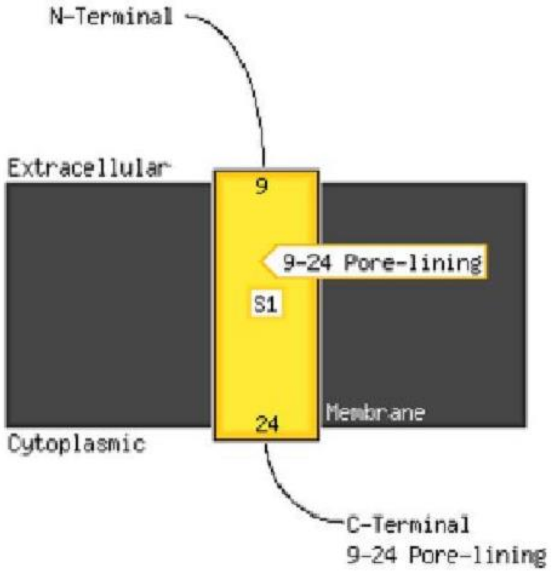

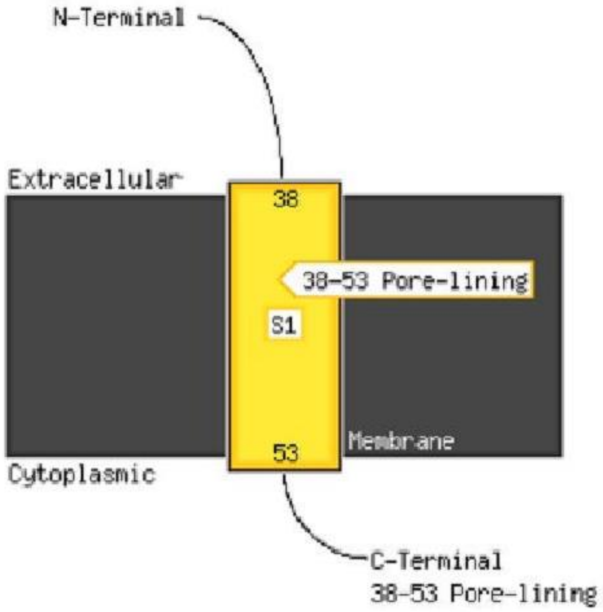

TaTIFY19 TaTIFY22

Supplementary Figure S6. Two dimensional plots showing the disorder state of TaTIFY proteins involved in binding.

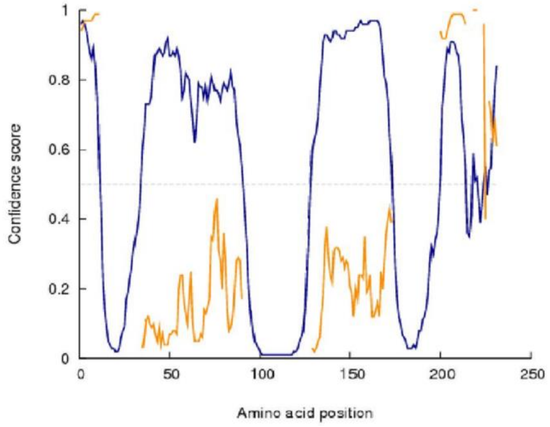

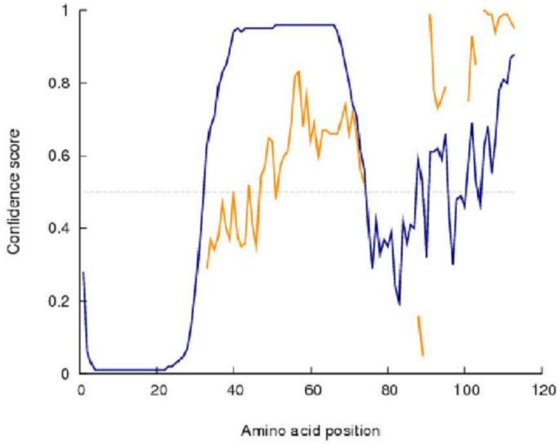

TaTIFY1 TaTIFY2

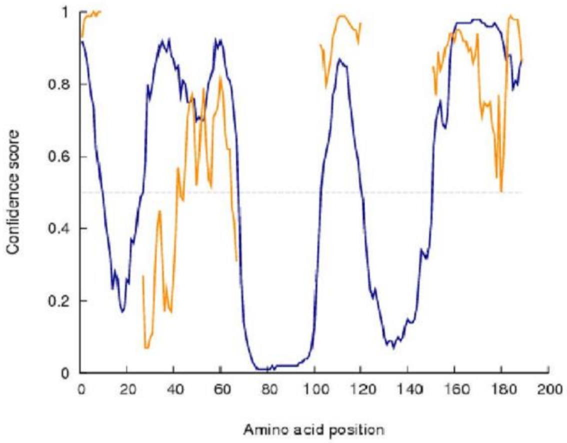

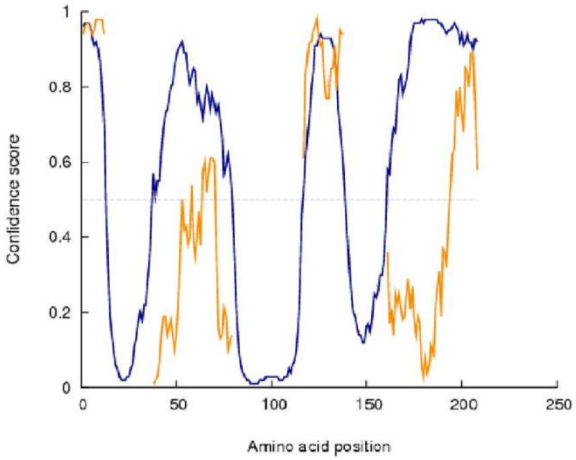

TaTIFY3 TaTIFY4

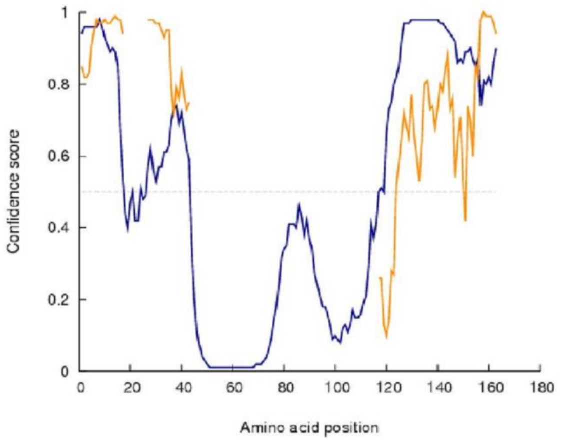

TaTIFY5

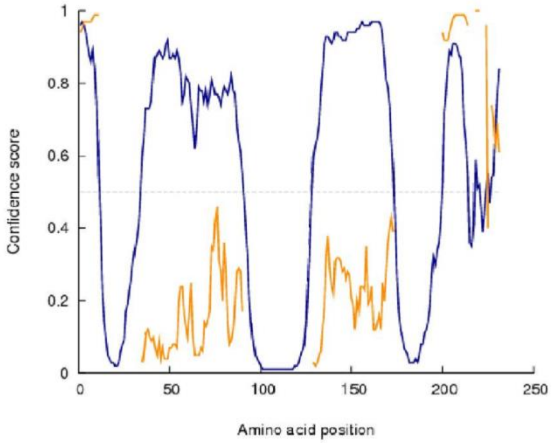

TaTIFY6

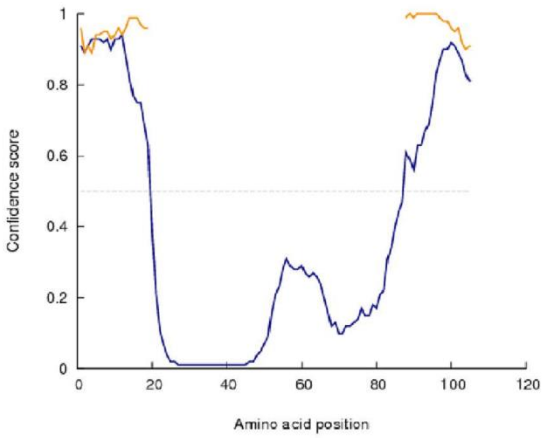

TaTIFY7

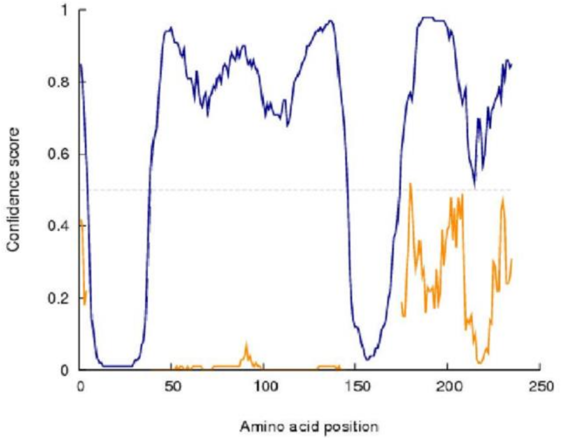

TaTIFY8

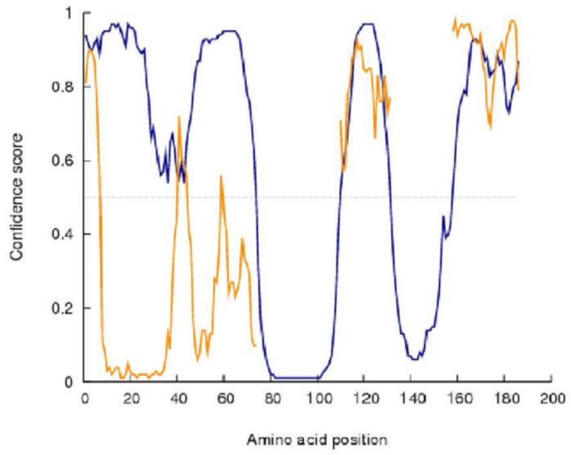

TaTIFY9

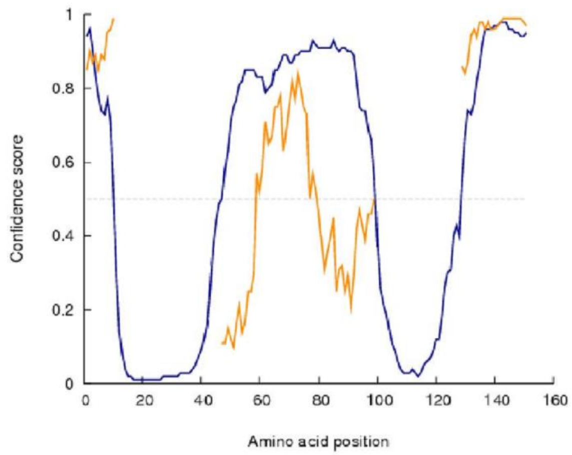

TaTIFY10

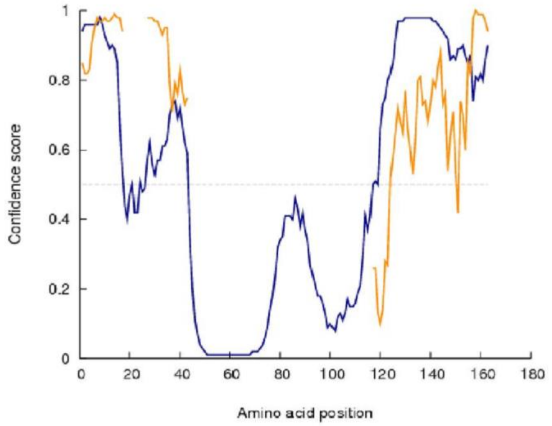

TaTIFY11

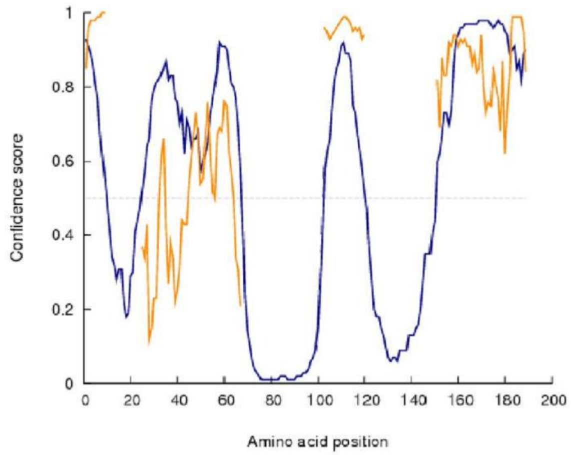

TaTIFY12

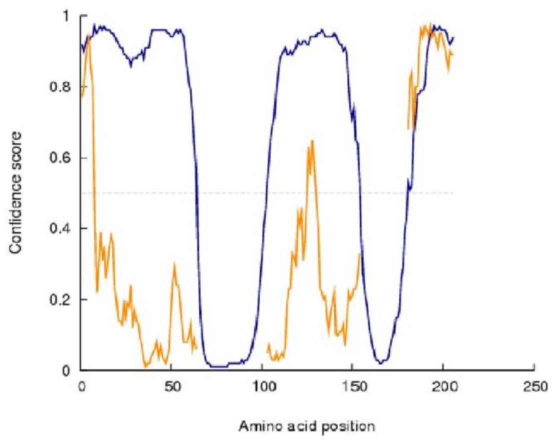

TaTIFY13

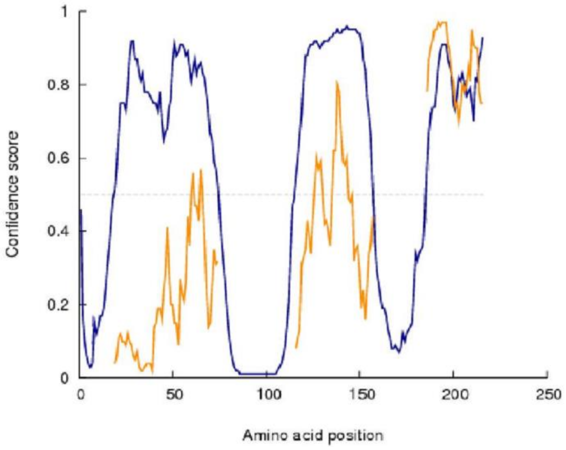

TaTIFY14

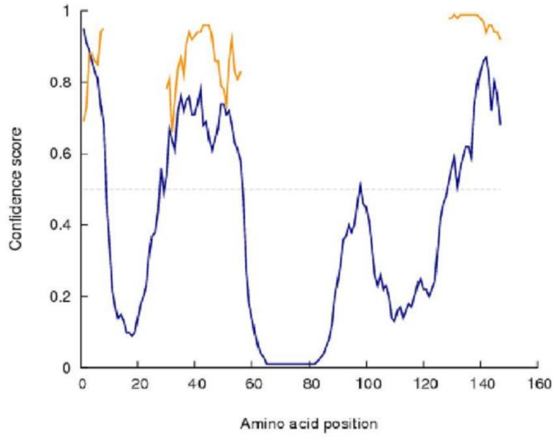

TaTIFY15

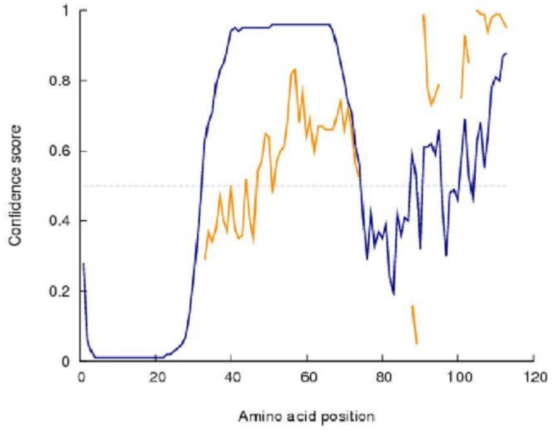

TaTIFY16

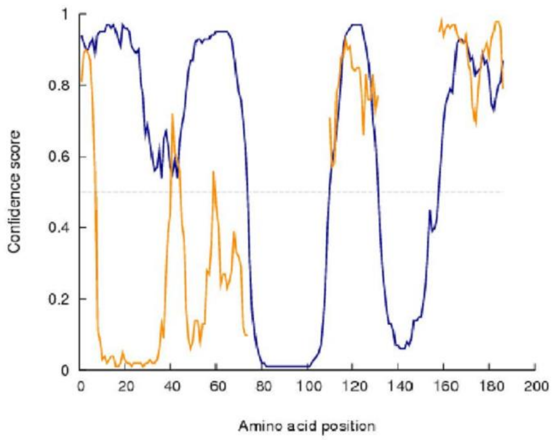

TaTIFY17

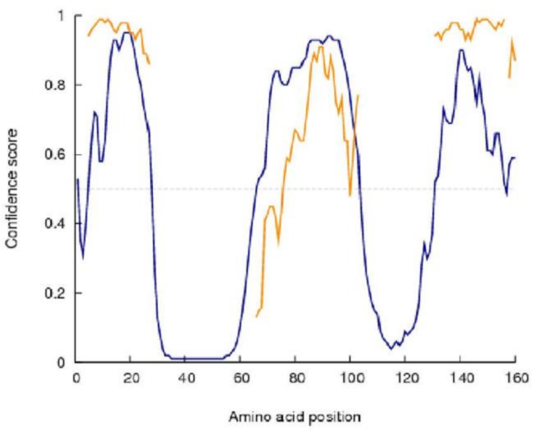

TaTIFY18

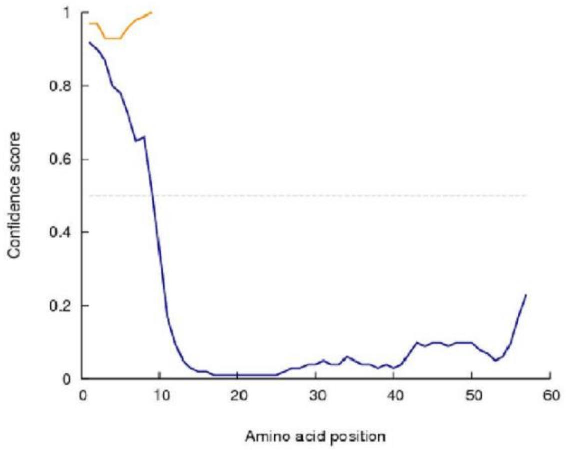

TaTIFY19

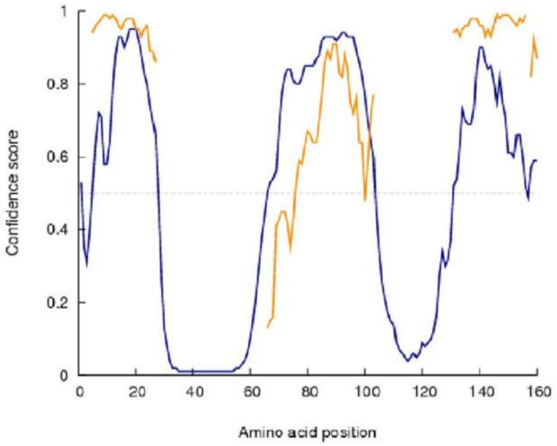

TaTIFY20

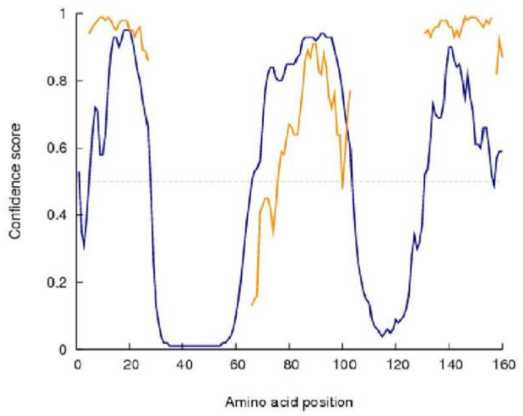

TaTIFY21

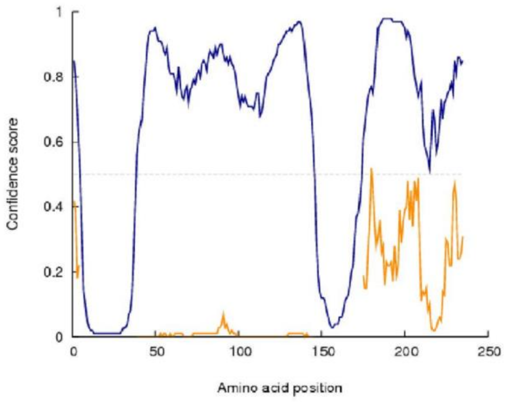

TaTIFY22

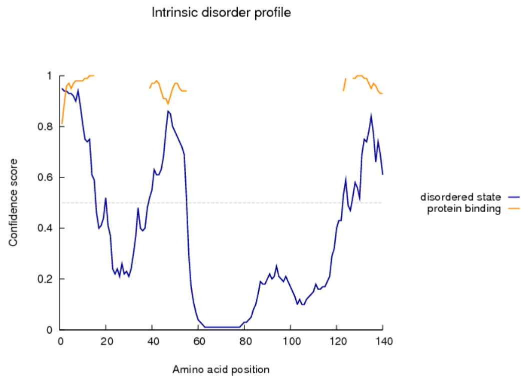

TaTIFY23

Supplementary Table S8 Prediction of sub-cellular localization of 23 newly identified TaTIFY TF sequences.

Name of TF	Subcellular localization
TaTIFY1	Chlo: 10, cyto: 1, mito: 2
TaTIFY2	Nucl: 4, chlo: 6
TaTIFY3	Chlo: 13
TaTIFY4	Chlo: 5, mito: 4, cyto:3, nucl: 2
TaTIFY5	Chlo: 11, nucl: 2
TaTIFY6	Chlo: 10, mito: 2, extr: 2
TaTIFY7	Cyto: 5, chlo: 2, mito: 5, nucl: 1
TaTIFY8	Chlo: 7, nucl: 1, extr: 4
TaTIFY9	Cyto: 4, chlo: 4, extr: 2, pero:2, mito: 1
TaTIFY10	Chlo: 5, extr: 4, mito: 3, nucl: 1
TaTIFY11	Nucl: 2, chlo: 11
TaTIFY12	Chlo: 14
TaTIFY13	Chlo: 3, nucl: 3.5, mito: 6
TaTIFY14	Mito: 6, nucl: 1, chlo: 5, cyto: 1
TaTIFY15	Chlo: 14
TaTIFY16	Chlo: 6, mito: 3, nucl: 4
TaTIFY17	Chlo: 4, cyto: 4, extr: 2, pero: 2, mito: 1
TaTIFY18	Chlo: 4, mito: 9
TaTIFY19	Chlo: 3, extr: 11
TaTIFY20	Chlo: 4, mito: 9
TaTIFY21	Chlo: 3, mito: 8
TaTIFY22	Chlo: 7, extr: 4, plas: 4, nucl: 1.5
TaTIFY23	Chlo: 13

Supplementary Figure S7. Tertiary structures of identified TaTIFY TF proteins showing actives sites

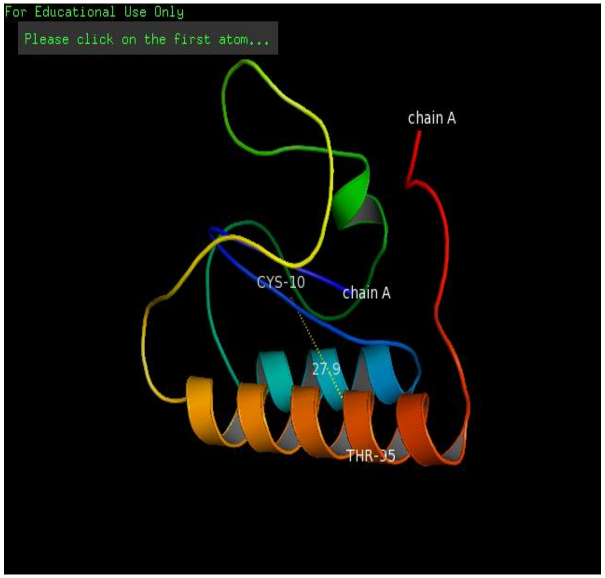

TaTIFY2 (active site: CYS 10-THR 35)

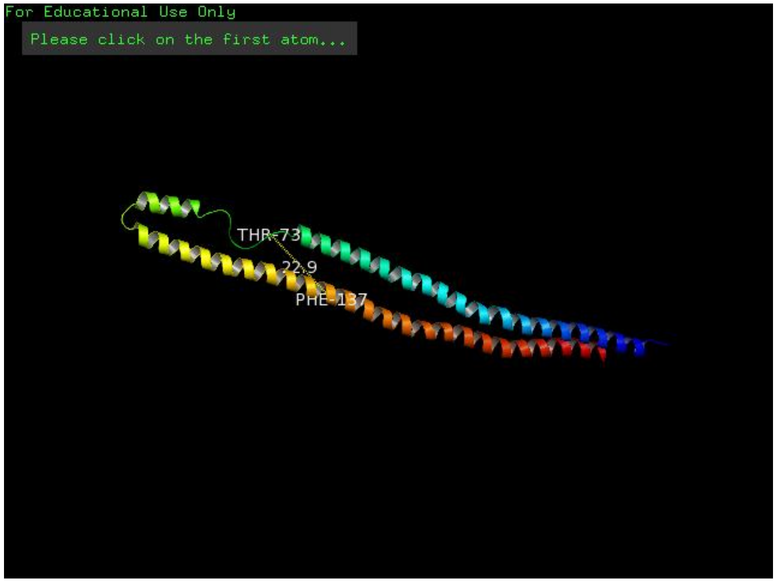

TaTIFY3 (active site:THR 37 - PHE 137)

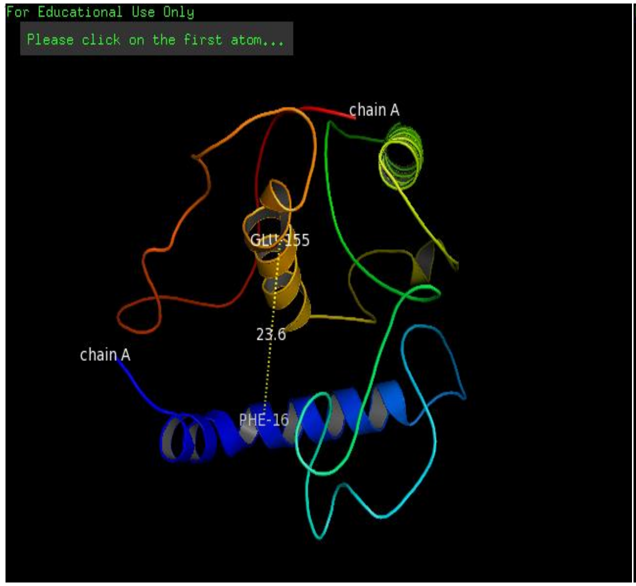

TaTIFY4 (active site: PHE 16 - GLU 155)

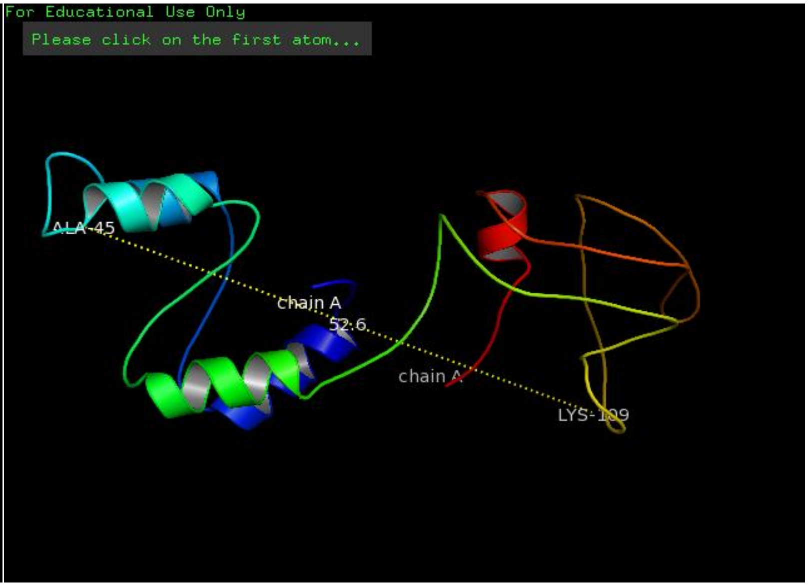

TaTIFY5 (active site: ALA 45 - LYS 109)

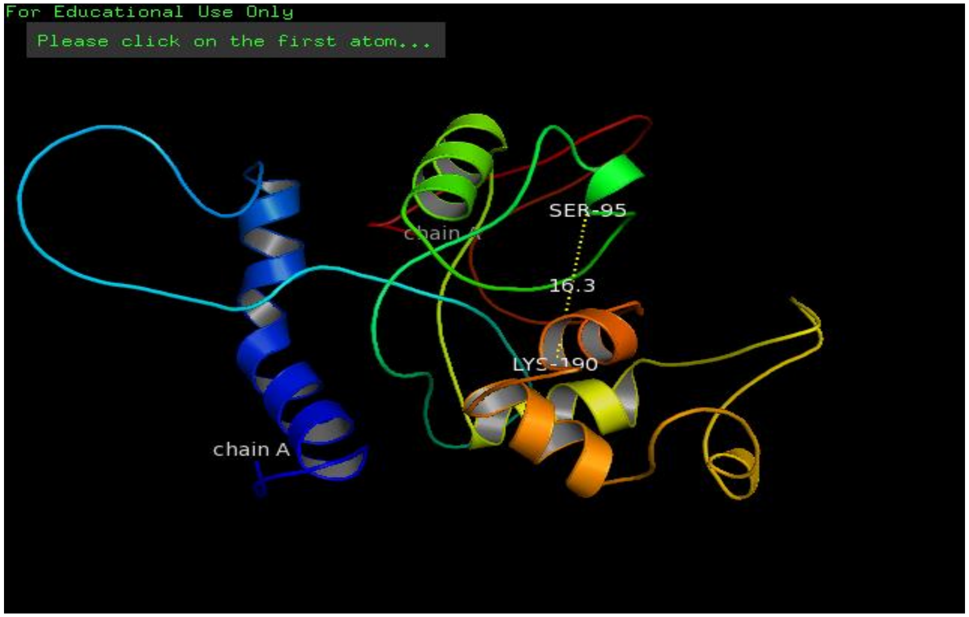

TaTIFY6 (active site: SER 95 - LYS 190)

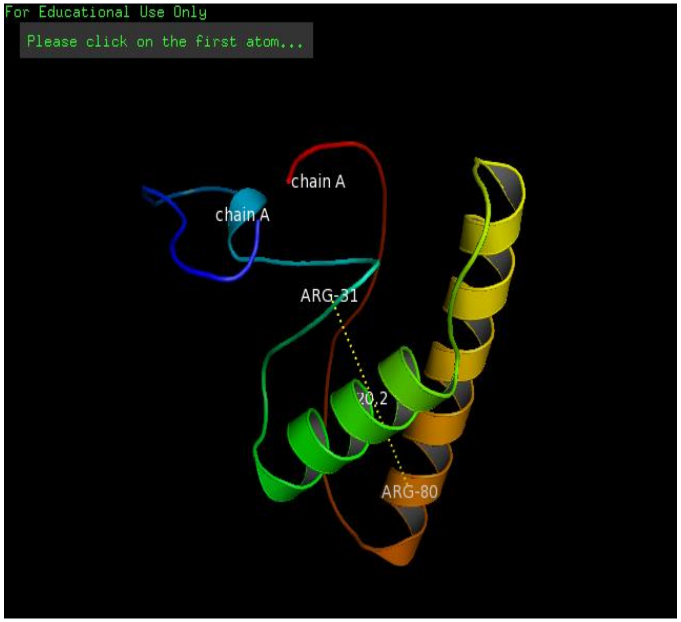

TaTIFY7 (active site: ARG 31 - ARG 80)

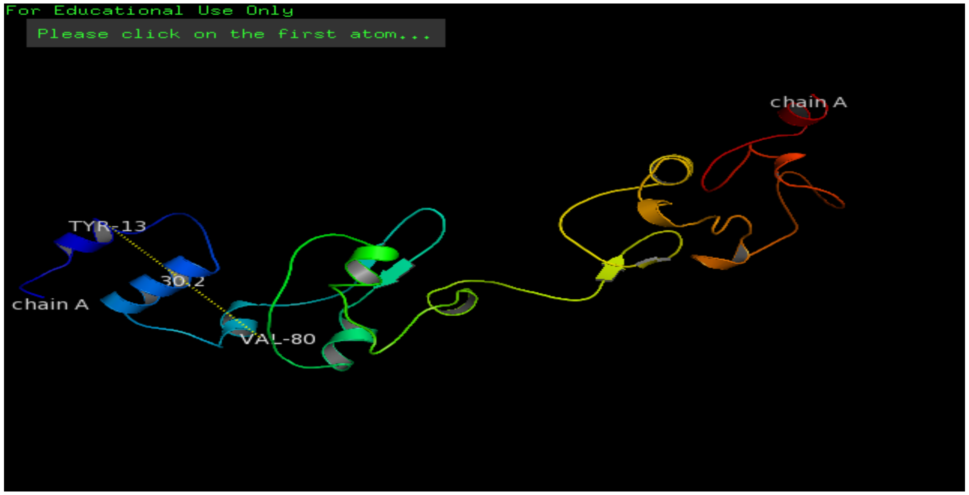

TaTIFY8 (active site: TYR 13 - VAL 80)

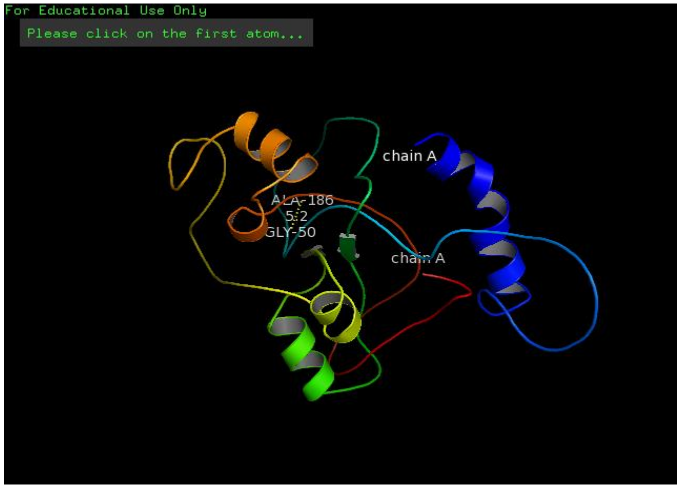

TaTIFY9 (active site: GLY 50 - ALA 186)

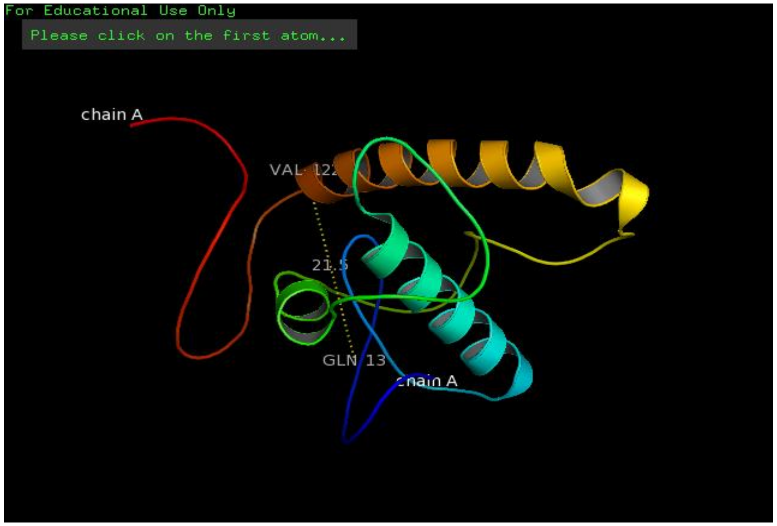

TaTIFY10 (active site: GLN 13 - VAL 122)

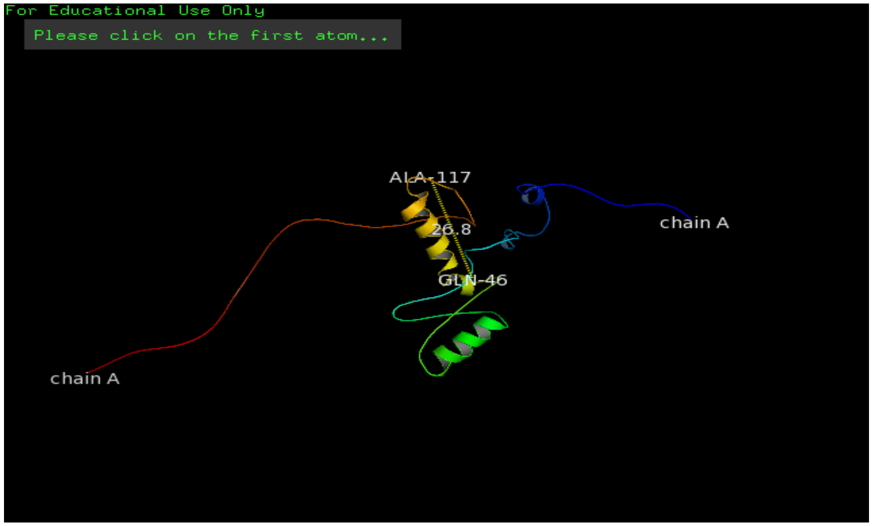

TaTIFY11 (active site: GLN 46 - ALA 117)

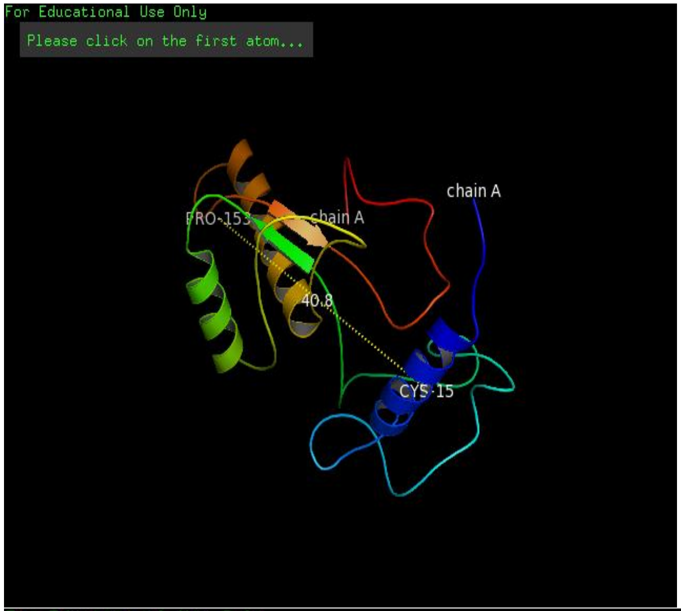

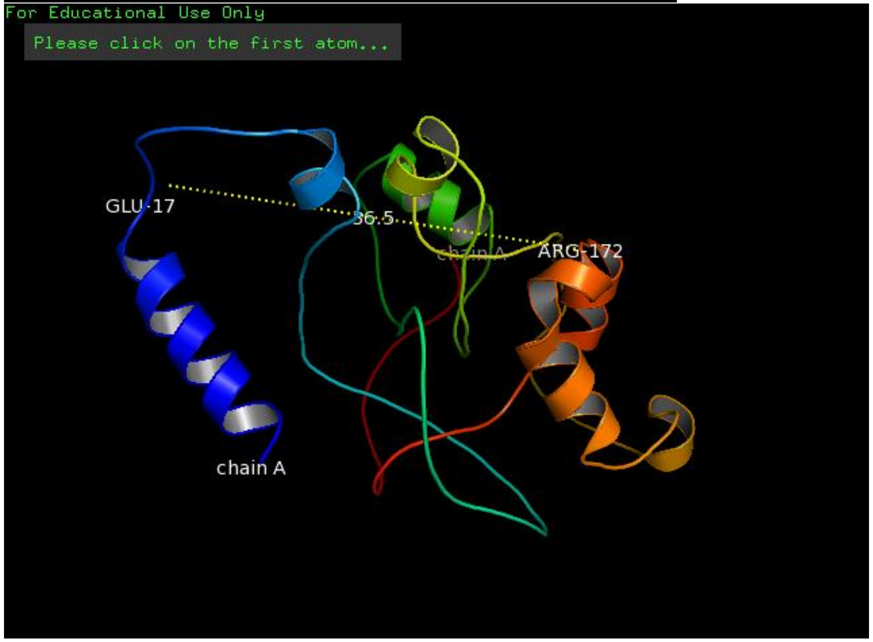

TaTIFY12 (active site: CYS 15 - PRO 153)

TaTIFY13 (active site: GLU 17 - ARG 172)

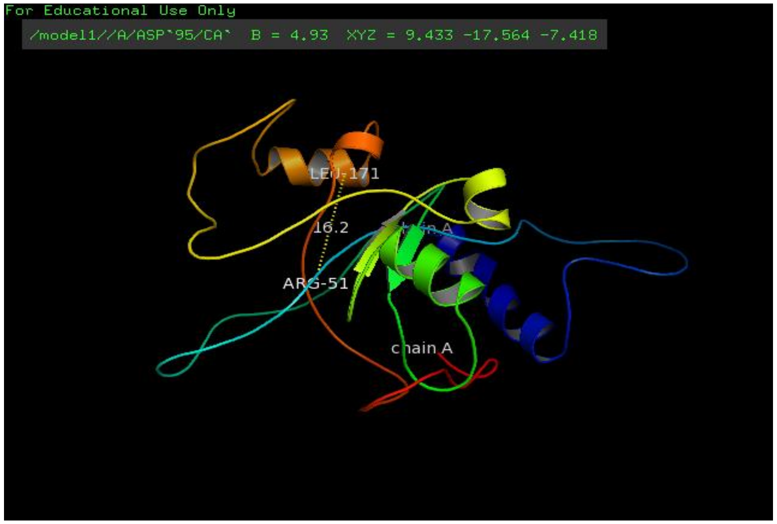

TaTIFY14 (active site: ARG 51 - LEU 171)

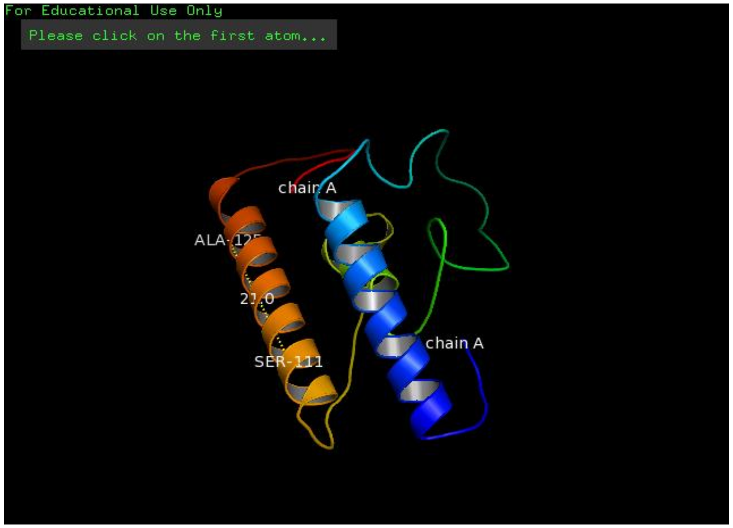

TaTIFY15 (active site: SER 111 - ALA 125)

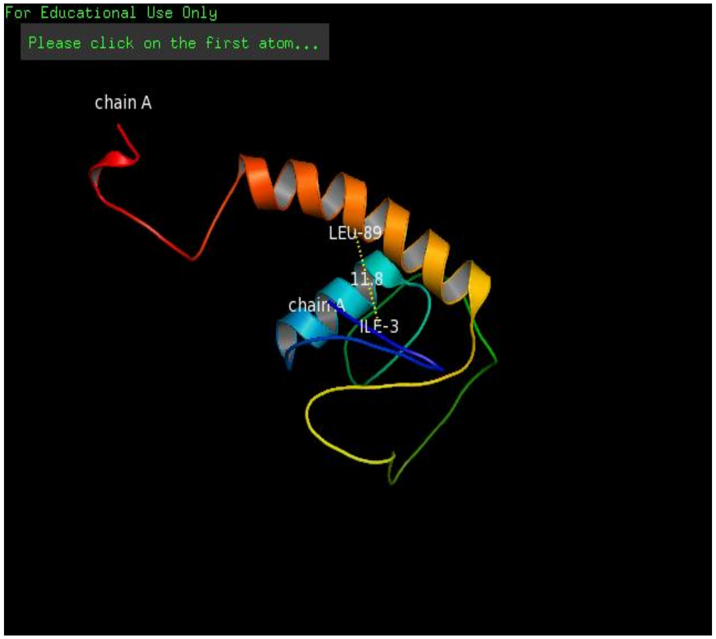

TaTIFY16 (active site: ILE 3 - LEU 89)

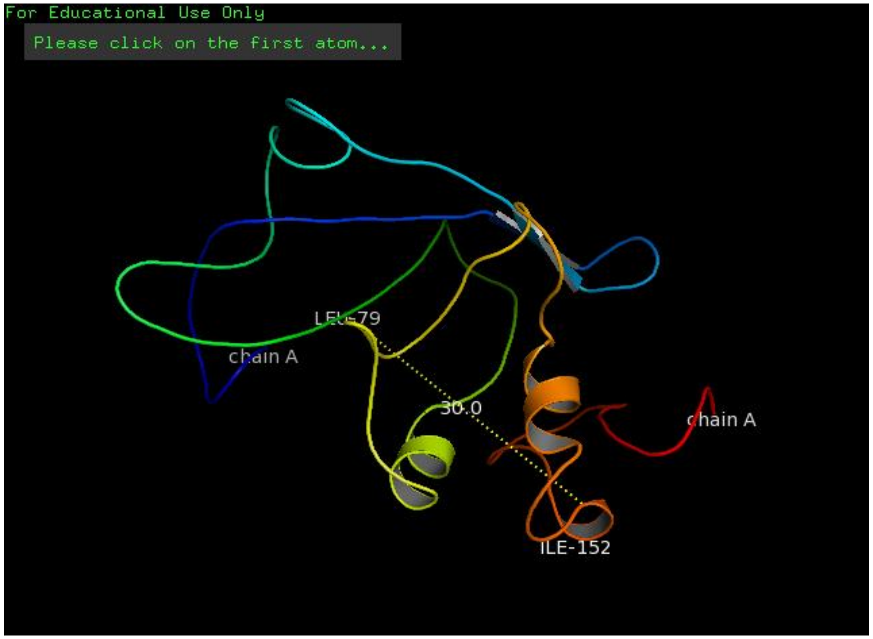

TaTIFY17 (active site: LEU 79 - ILE 152)

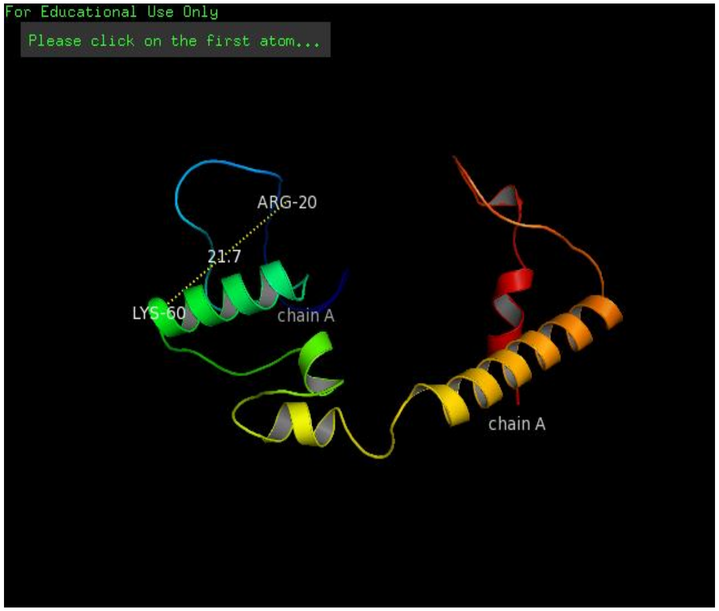

TaTIFY18 (active site: ARG 20 - LYS 60)

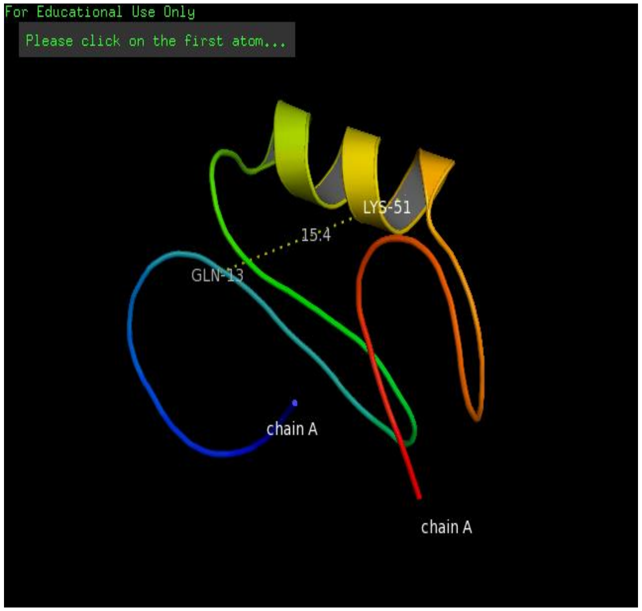

TaTIFY19 (active site: GLN 13 - LYS 51)

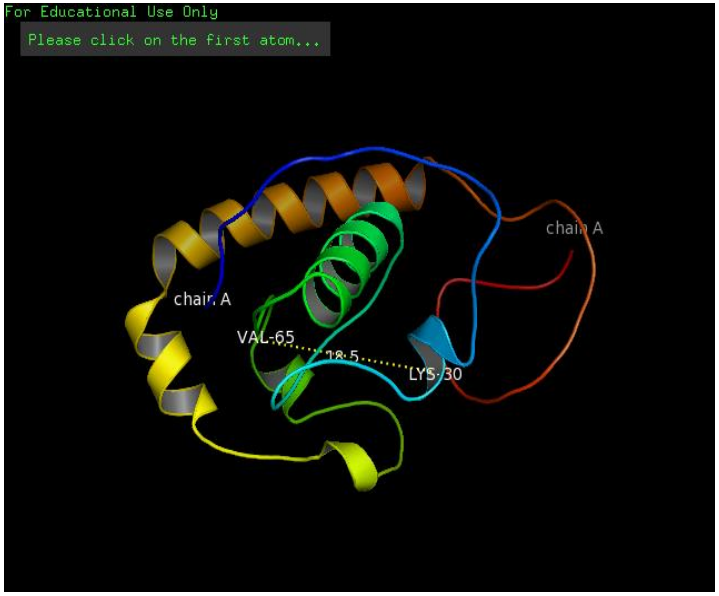

TaTIFY20 (active site: LYS 30 - VAL 65)

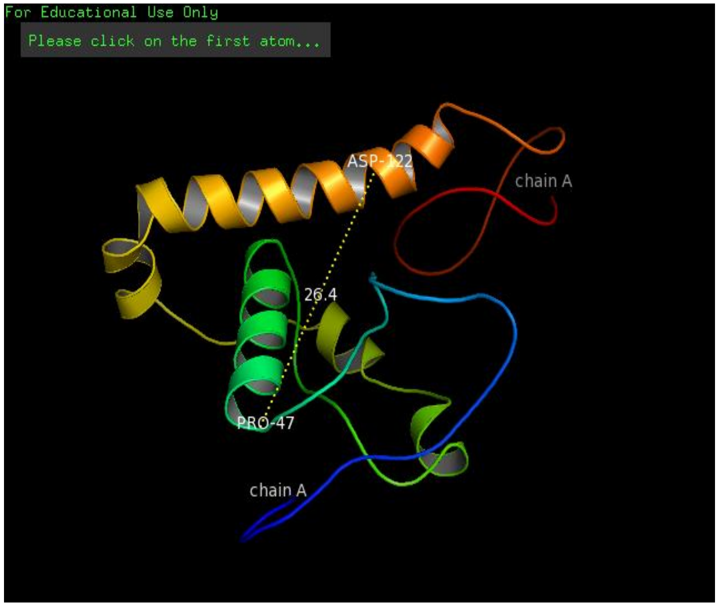

TaTIFY21 (active site: PRO 47- ASP 122)

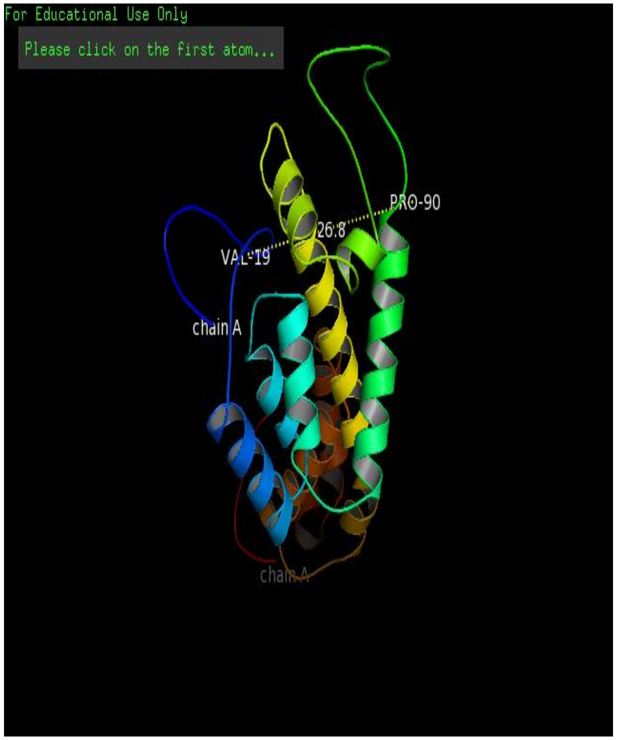

TaTIFY22 (active site: VAL 19 - PRO 90)

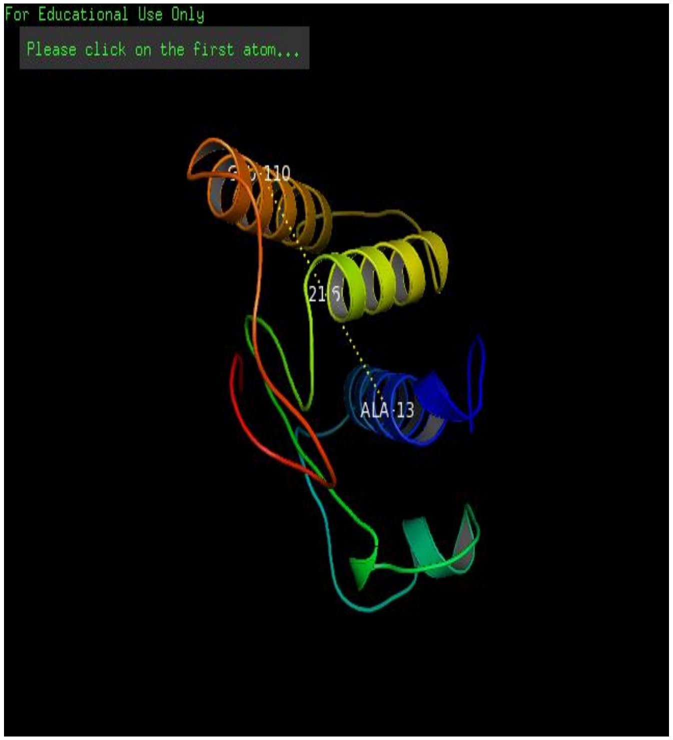

TaTIFY23 (active site: ALA 13 - GLN 110)

Supplementary Figure S8. Validation of stereo-chemical properties of the tertiary structures through Ramachandran plot

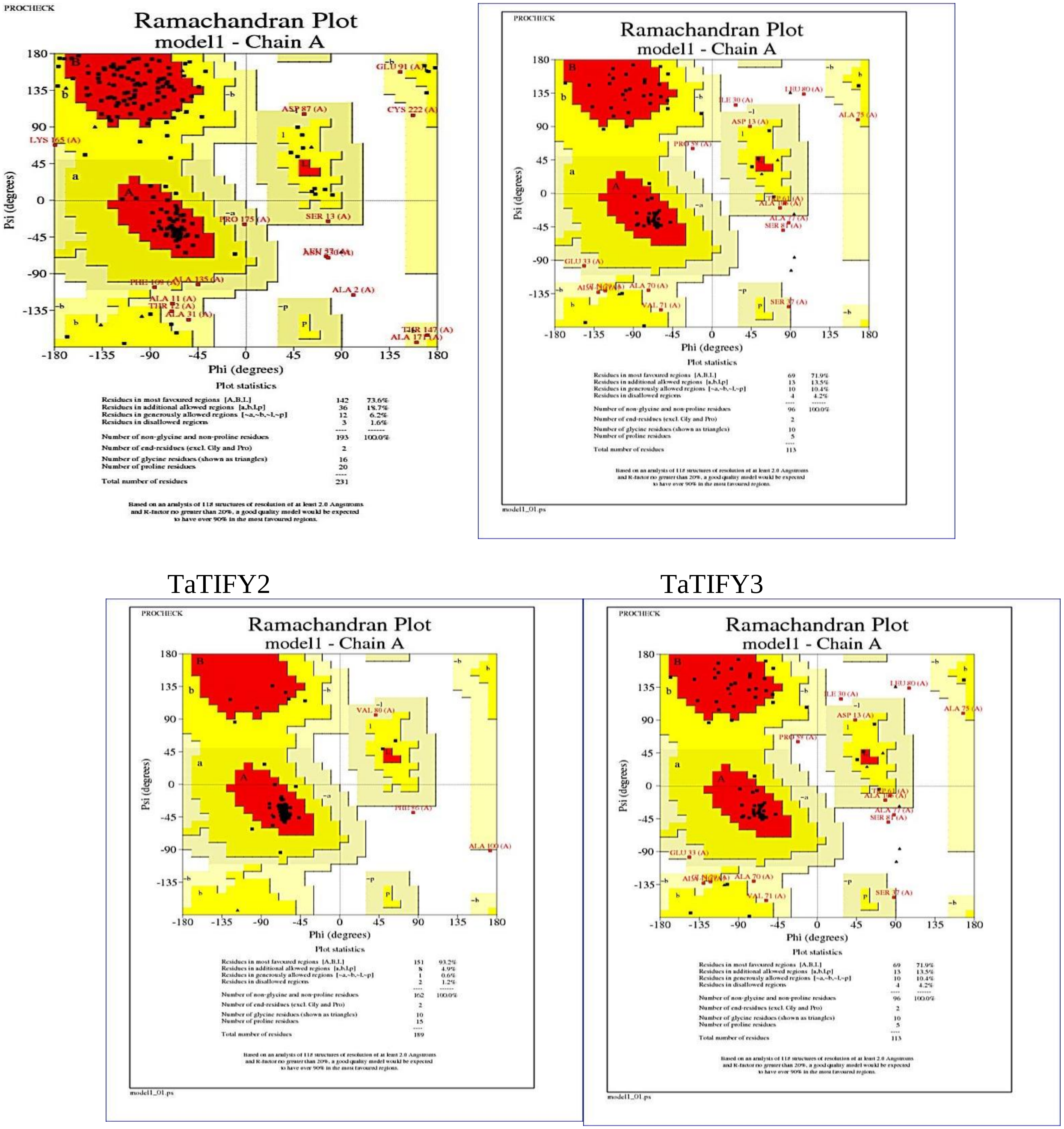

TaTIFY4

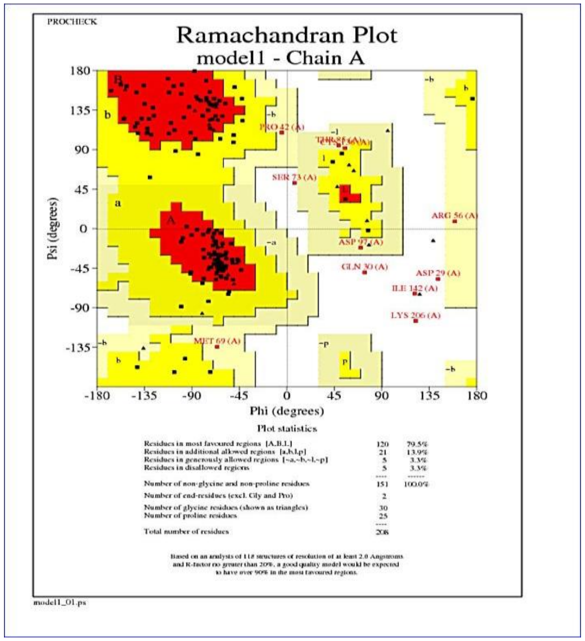

TaTIFY5

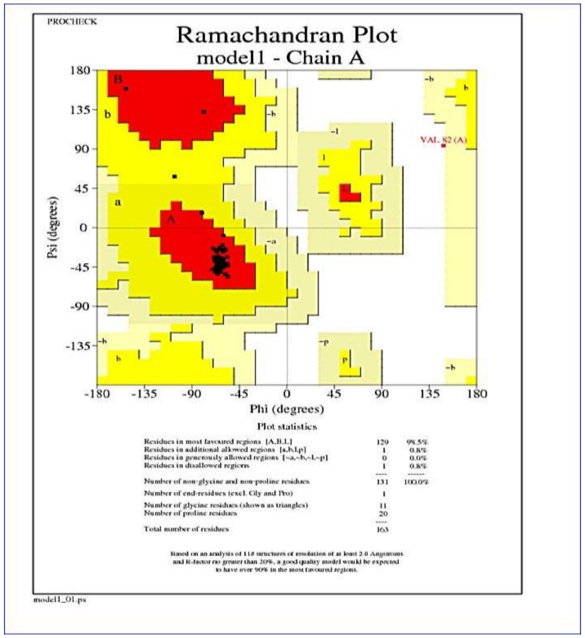

TaTIFY6

TaTIFY7

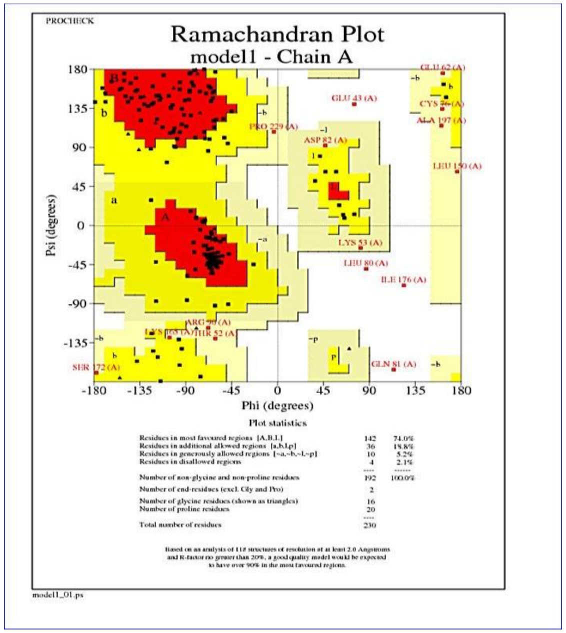

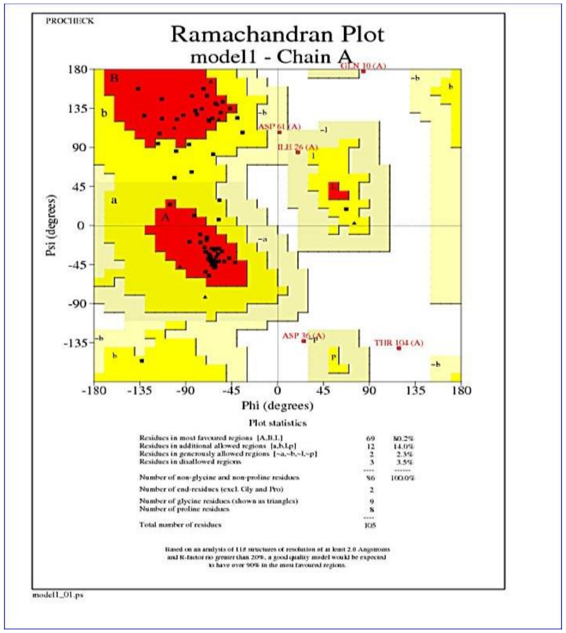

TaTIFY8 TaTIFY9

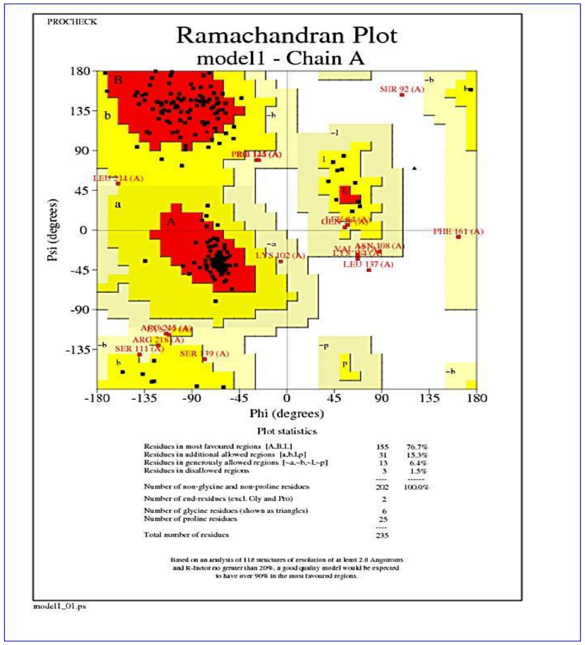

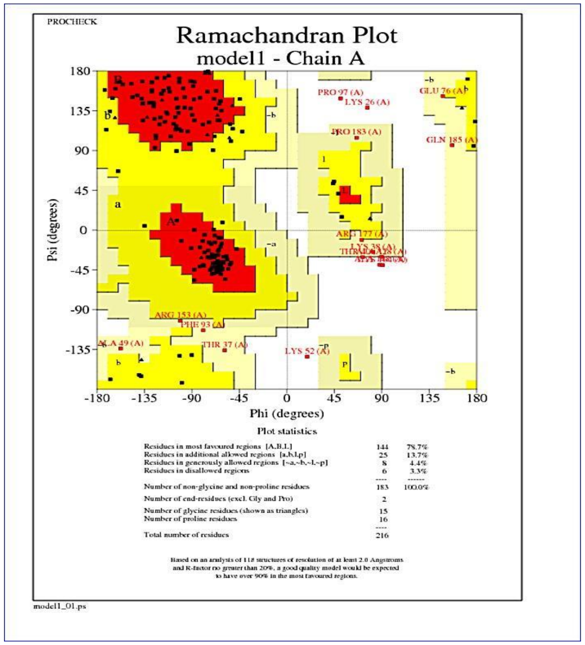

TaTIFY10

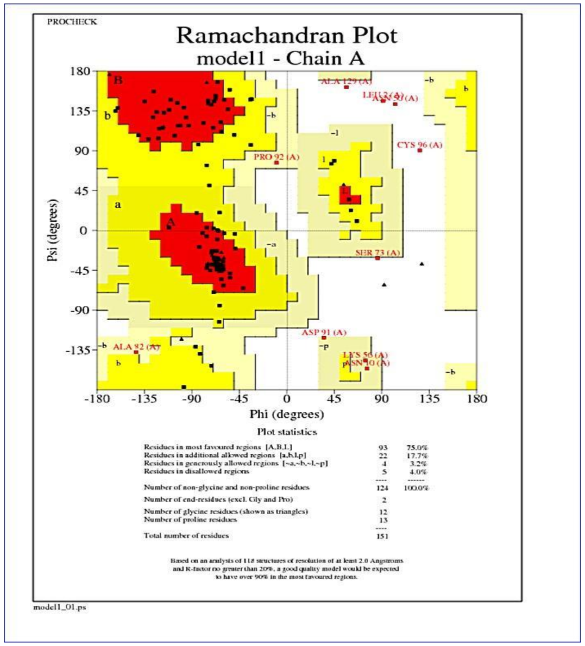

TaTIFY11

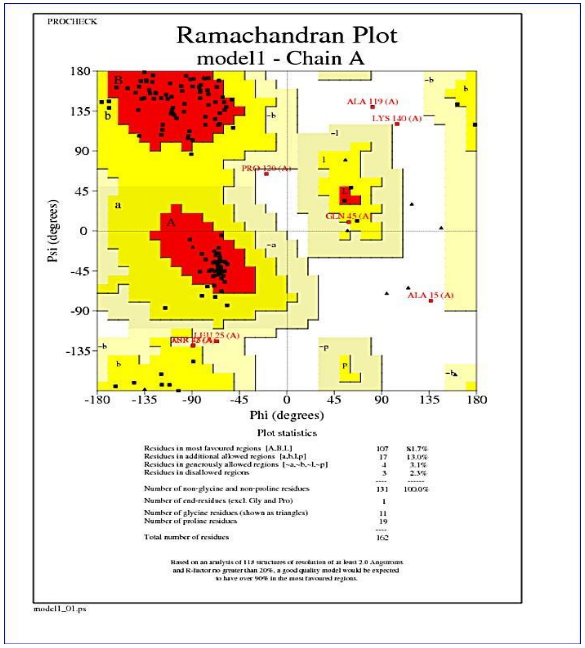

TaTIFY12

TaTIFY13

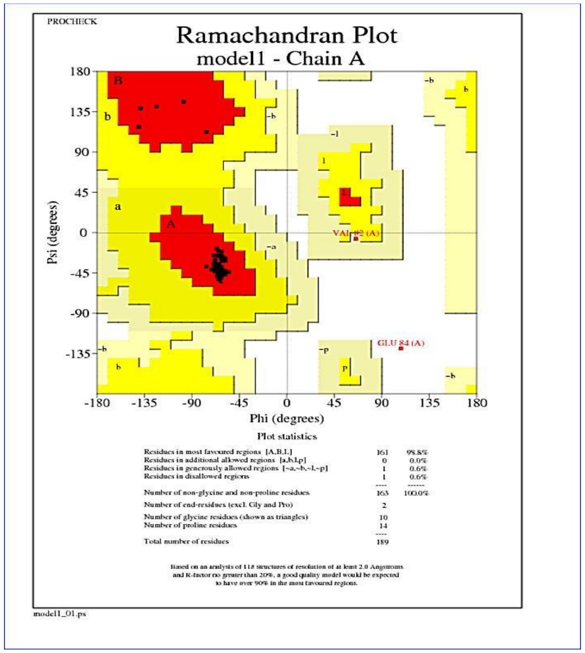

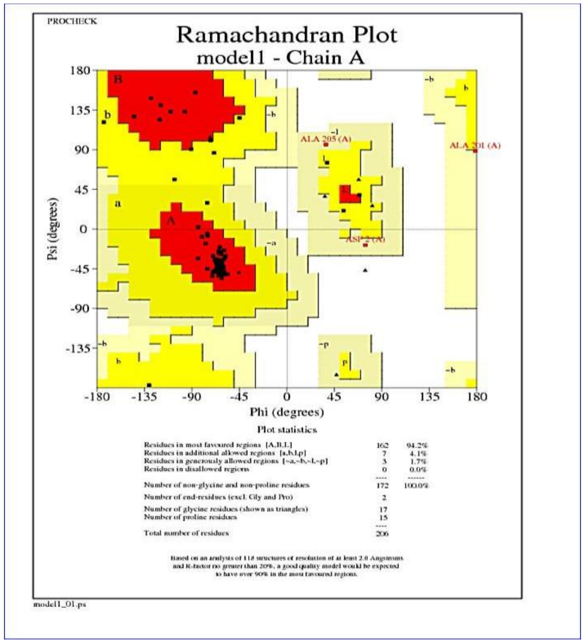

TaTIFY14

TaTIFY15

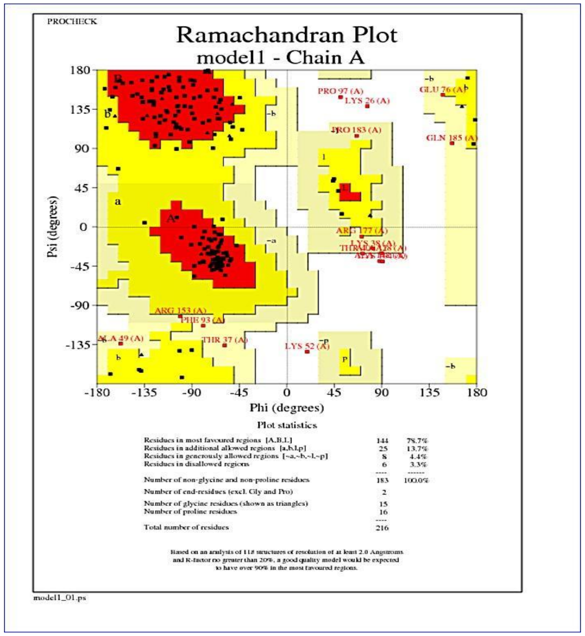

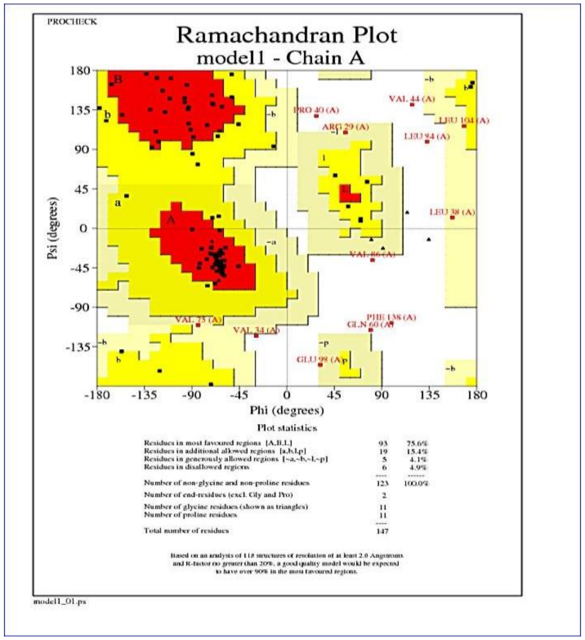

TaTIFY16 TaTIFY17

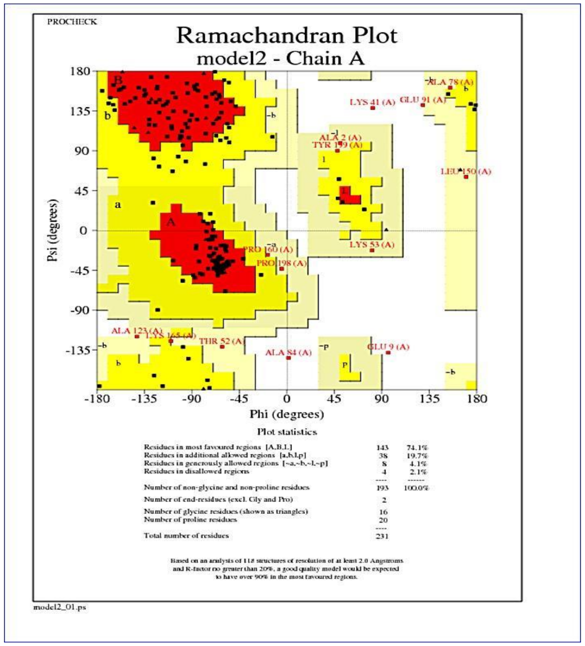

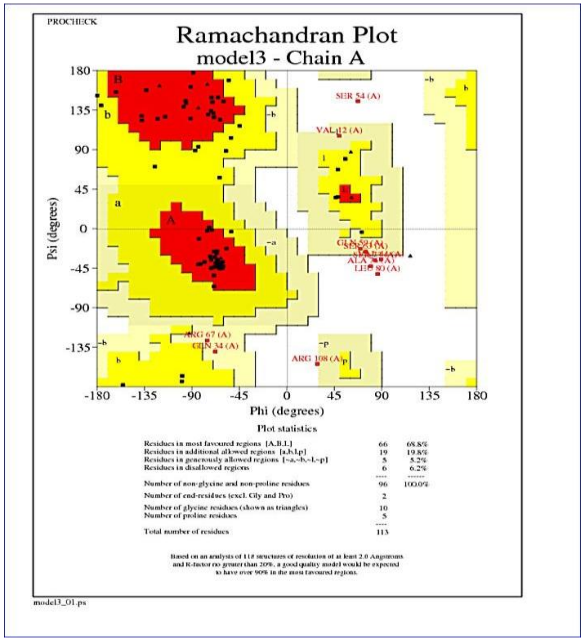

TaTIFY18 TaTIFY19

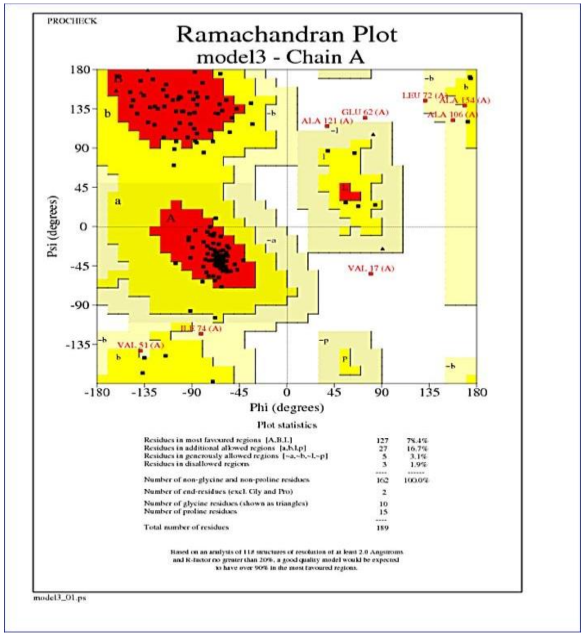

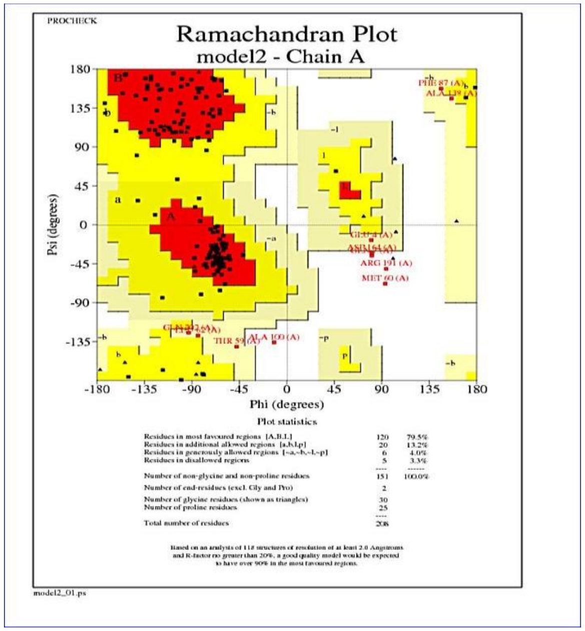

TaTIFY20 TaTIFY21

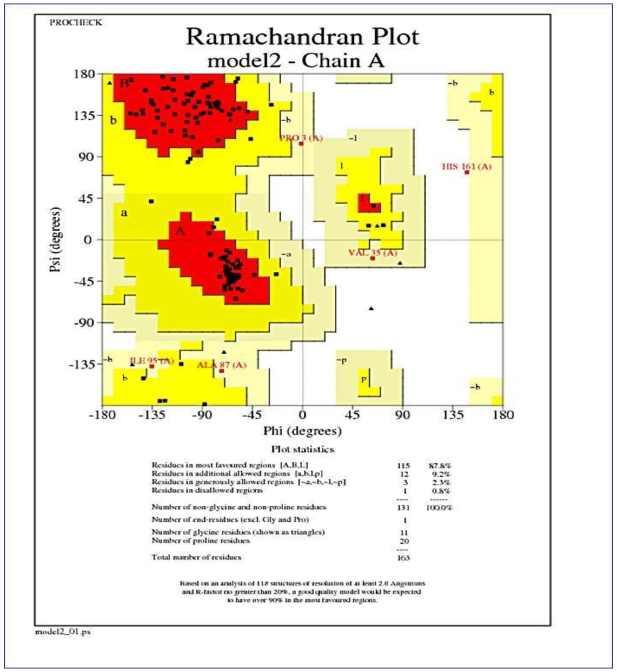

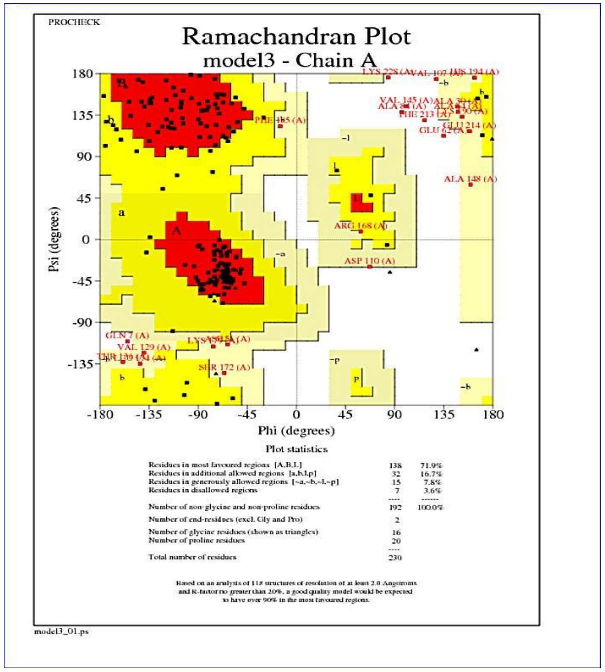

TaTIFY22

TaTIFY23

Supplementary Figure S9. Detailed structure of TaTIFY proteins showing major DNA binding sites. The detailed statistics are provided in Supplementary Table S8

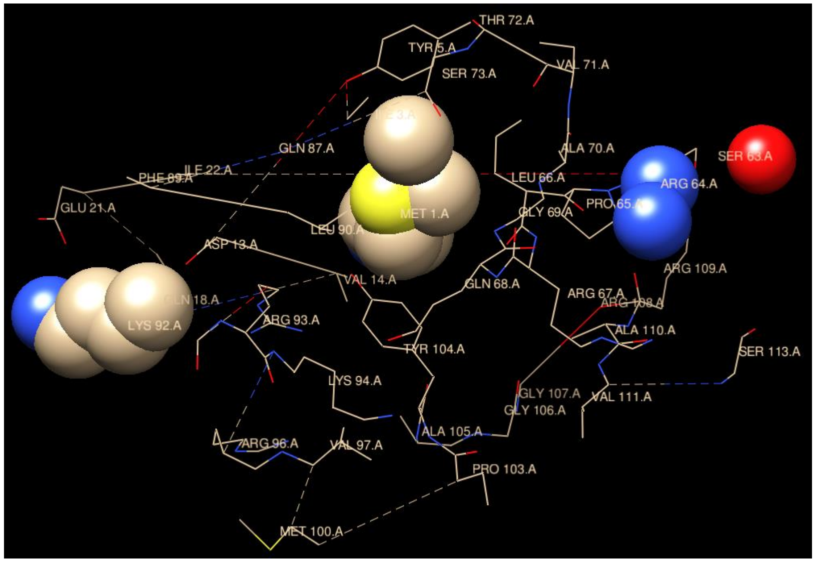

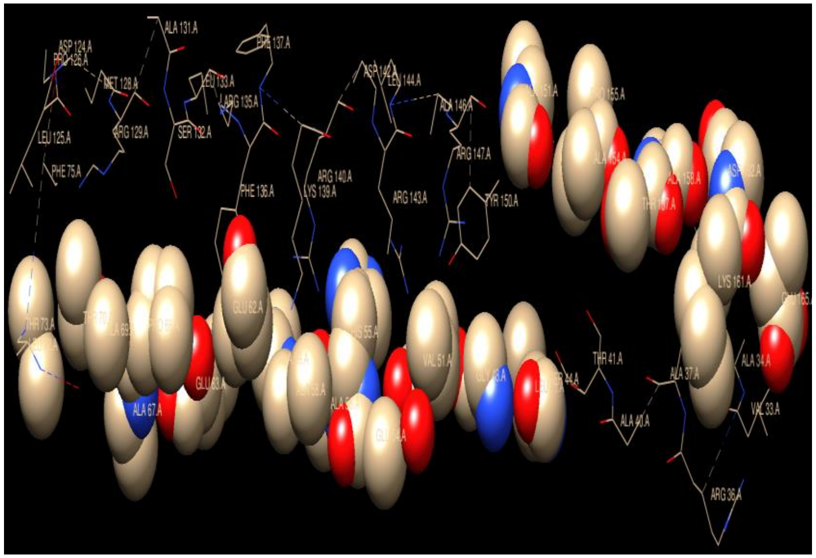

TaTIFY2

TaTIFY3

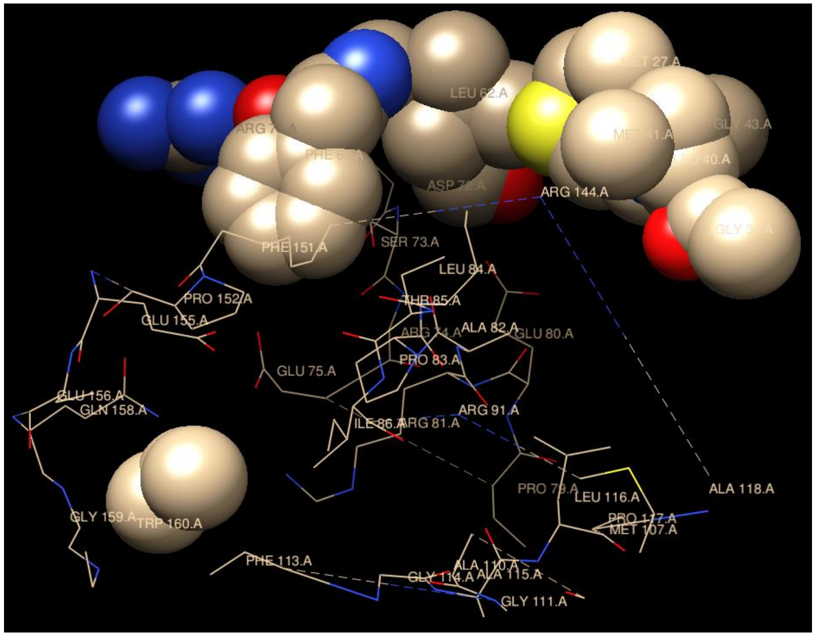

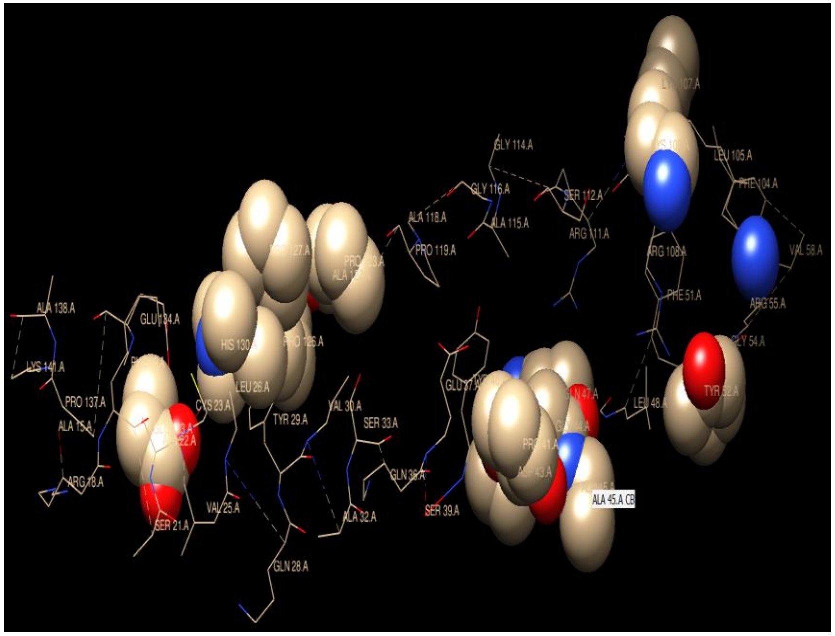

TaTIFY4 TaTIFY5

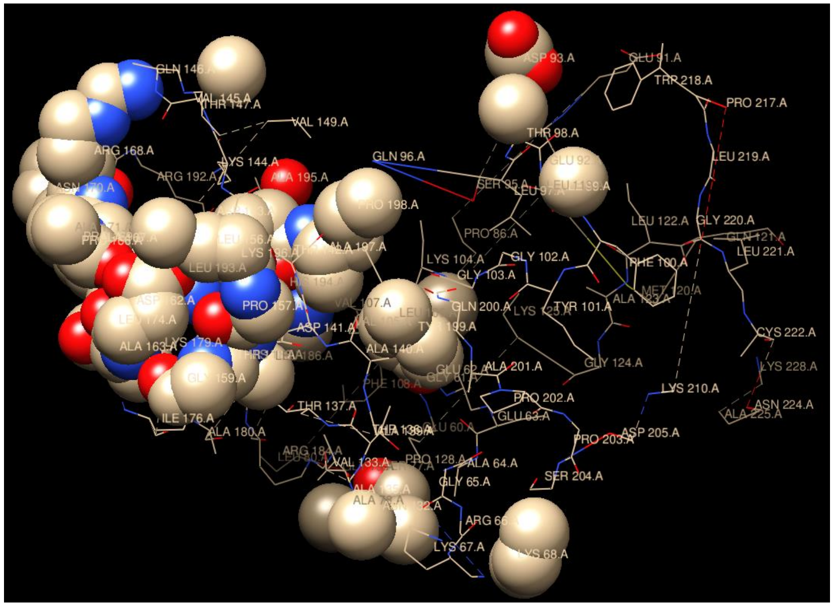

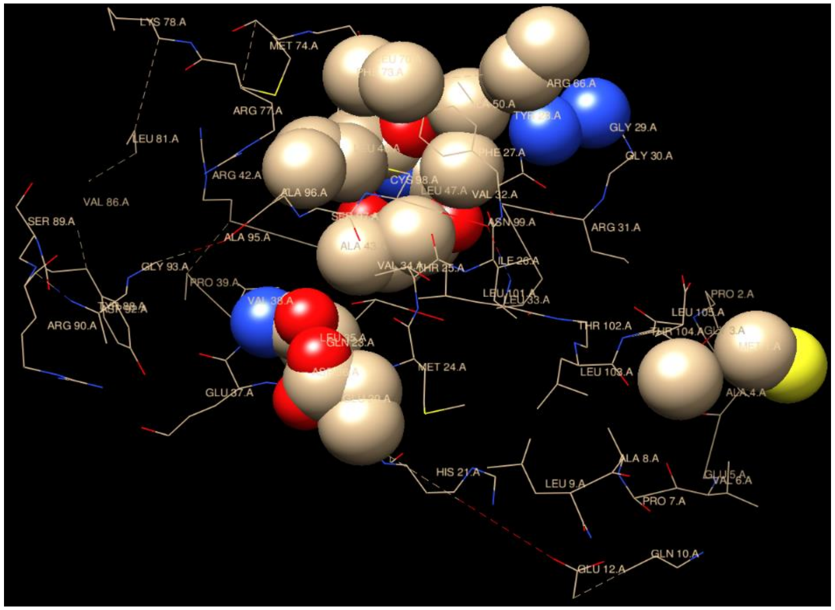

TaTIFY6 TaTIFY7

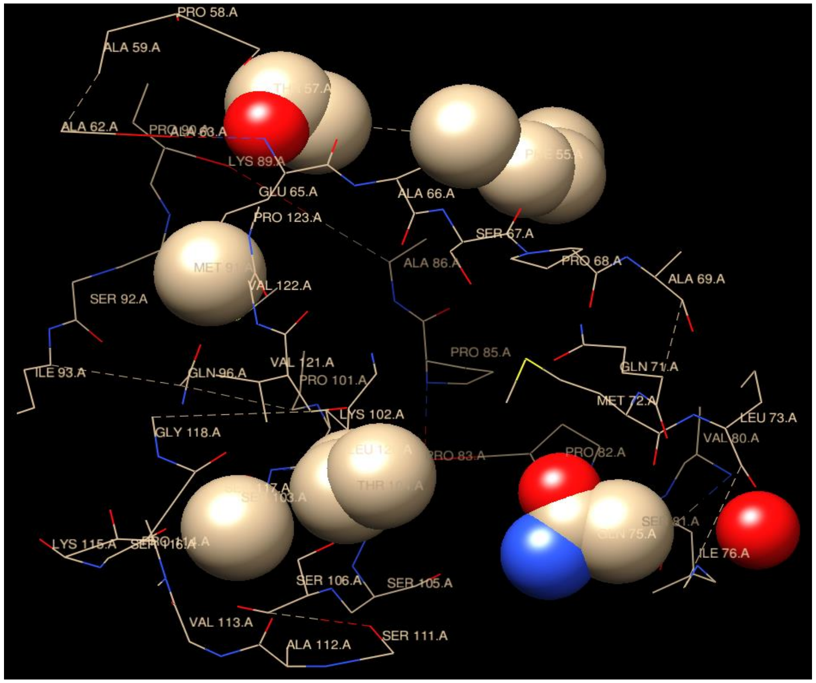

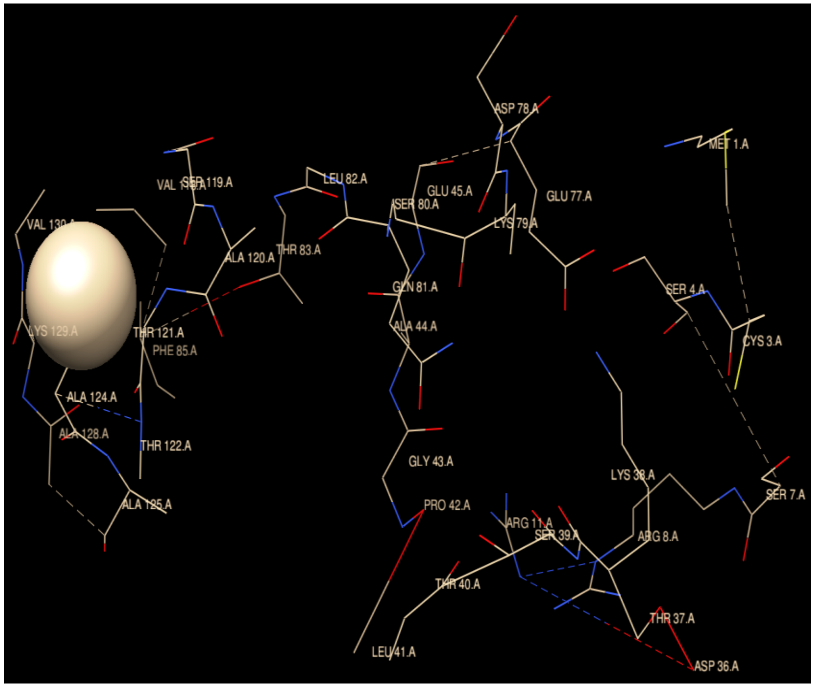

TaTIFY8

TaTIFY9

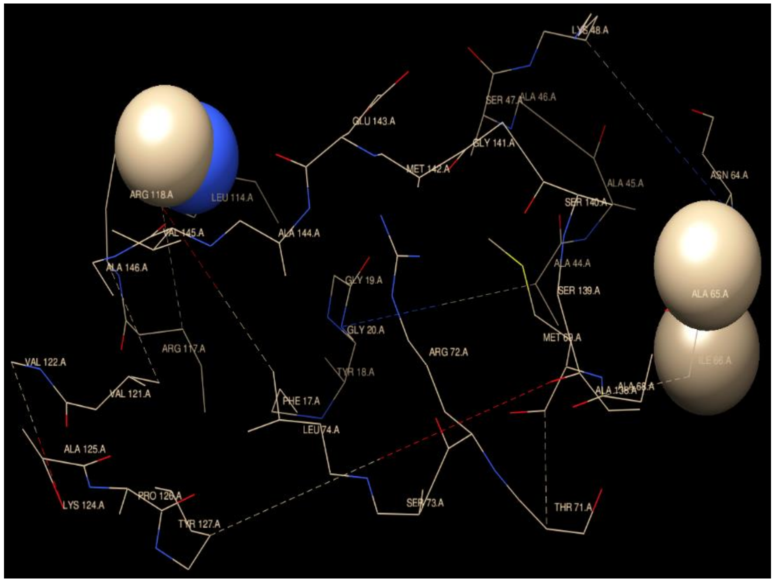

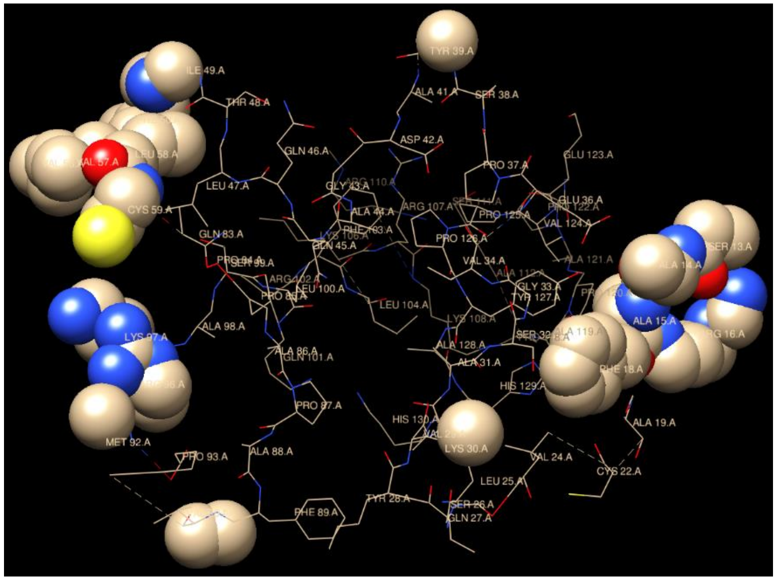

TaTIFY10

TaTIFY11

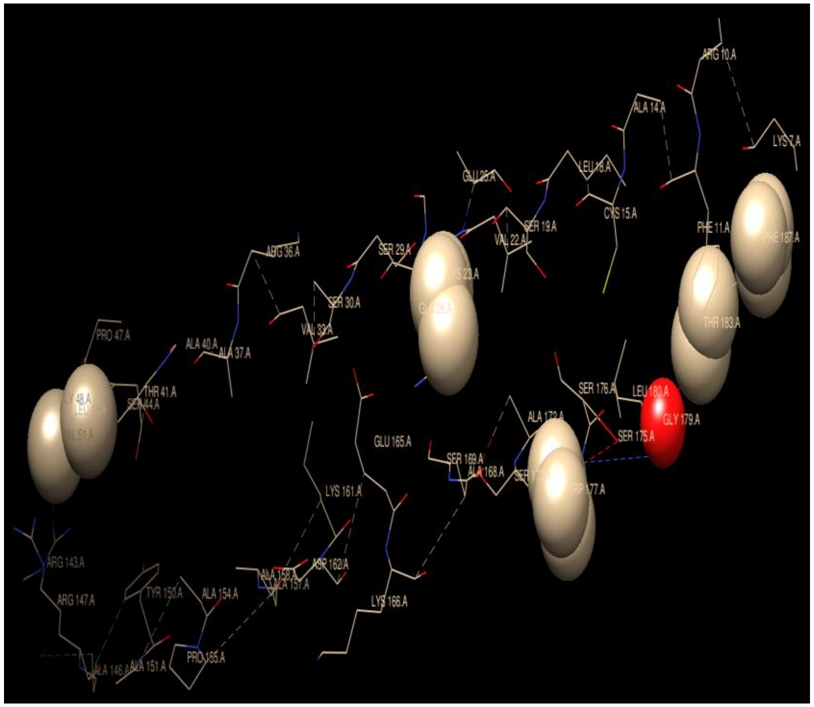

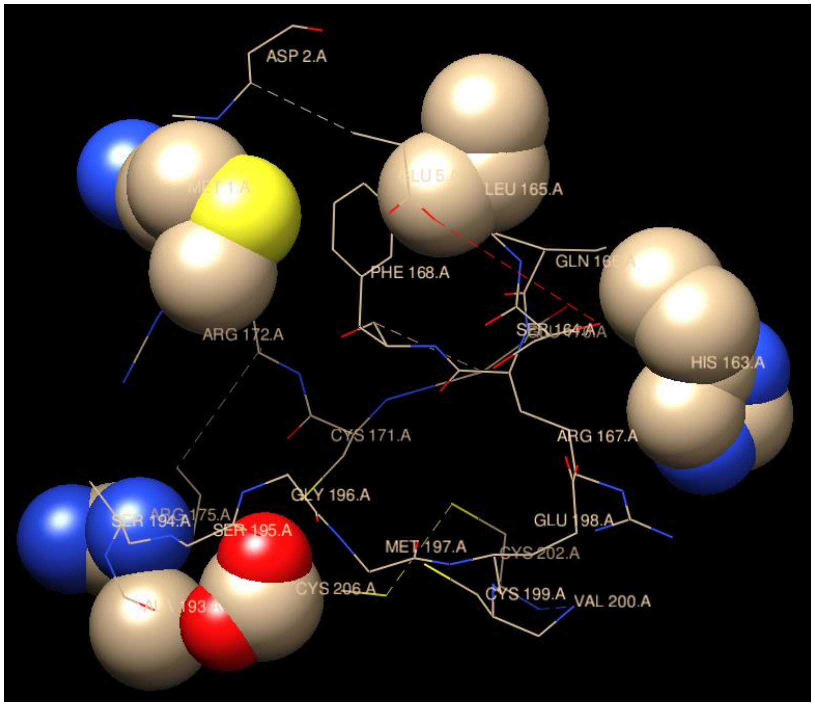

TaTIFY12

TaTIFY13

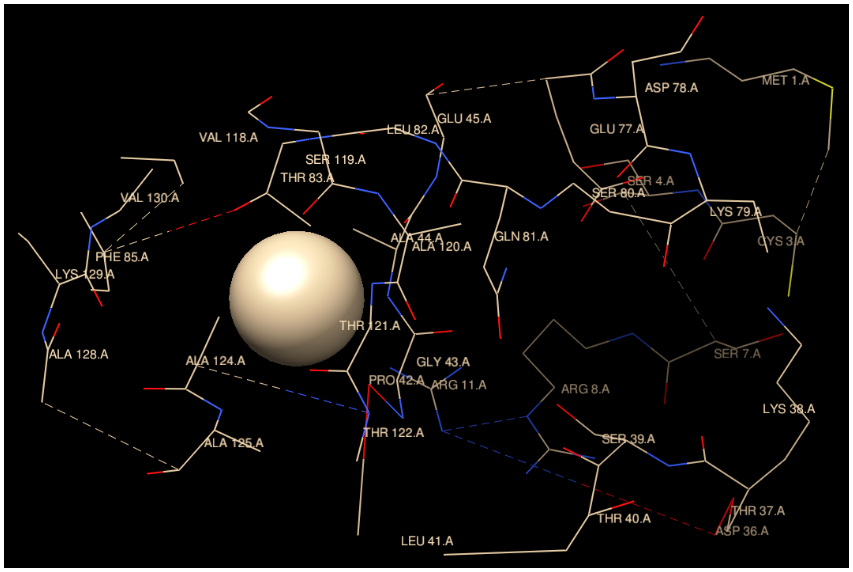

TaTIFY14

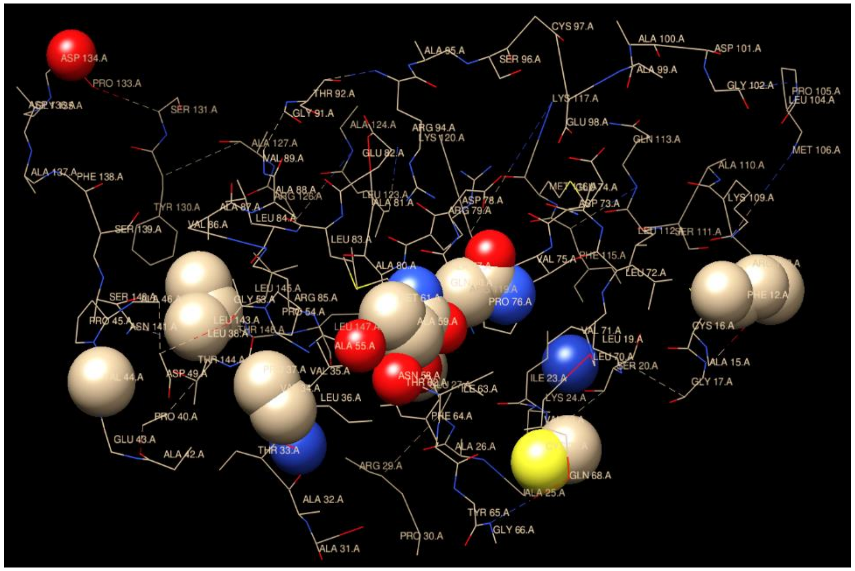

TaTIFY15

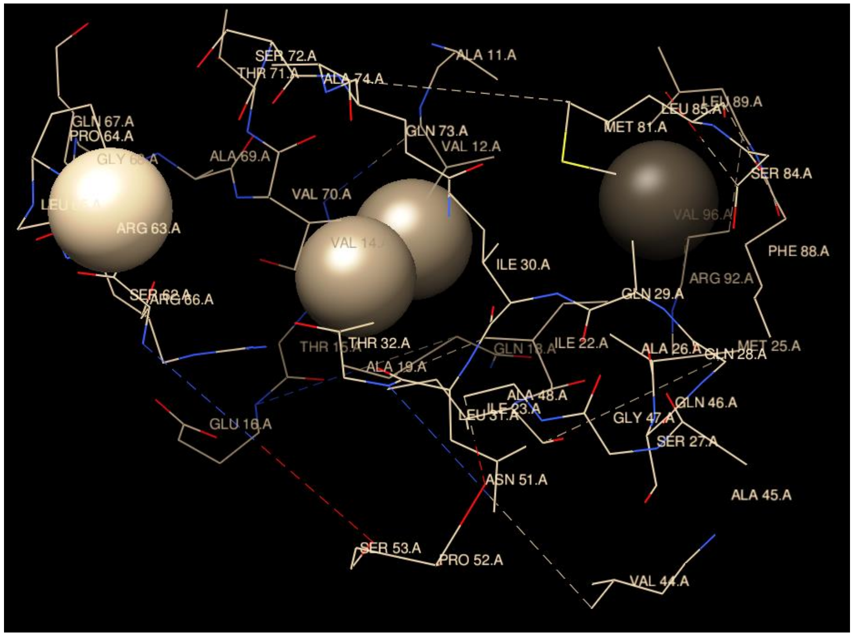

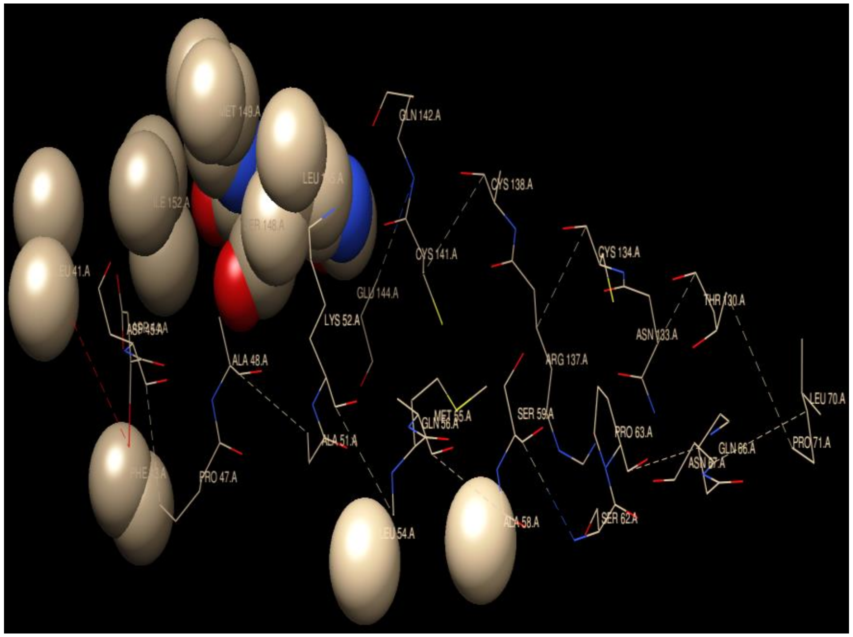

TaTIFY16

TaTIFY17

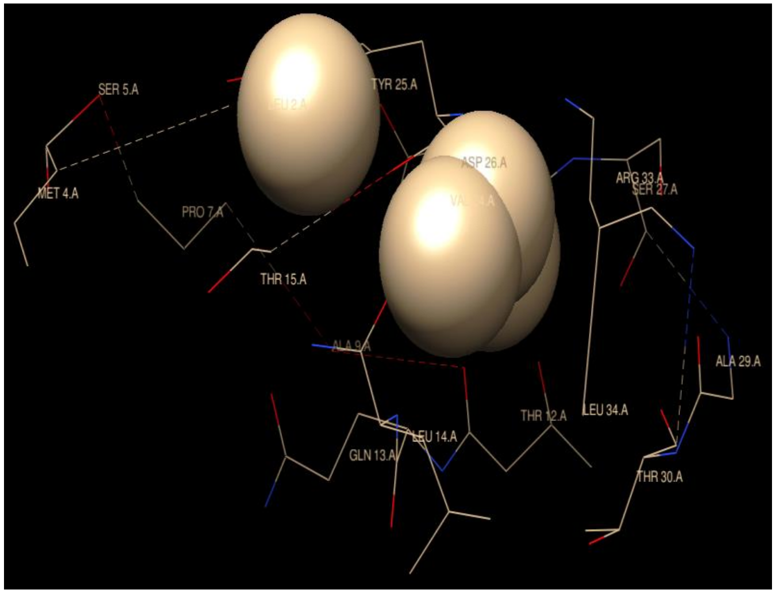

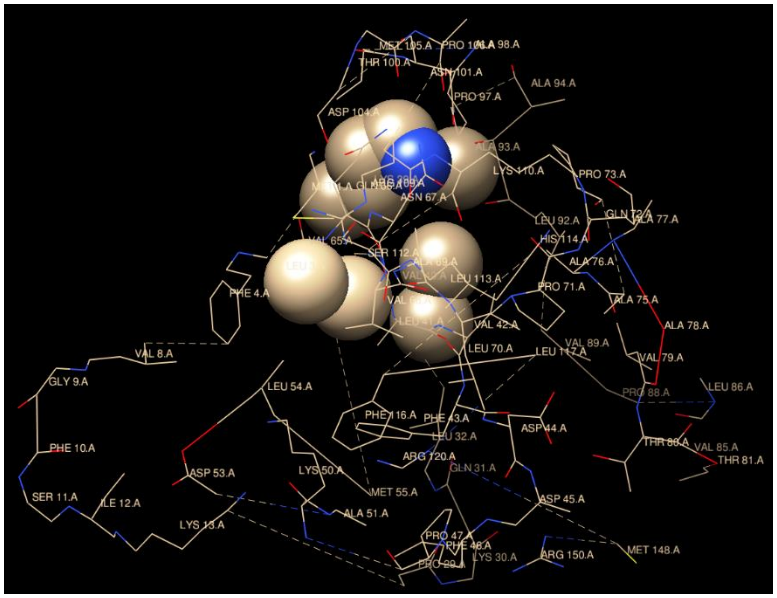

TaTIFY18

TaTIFY19

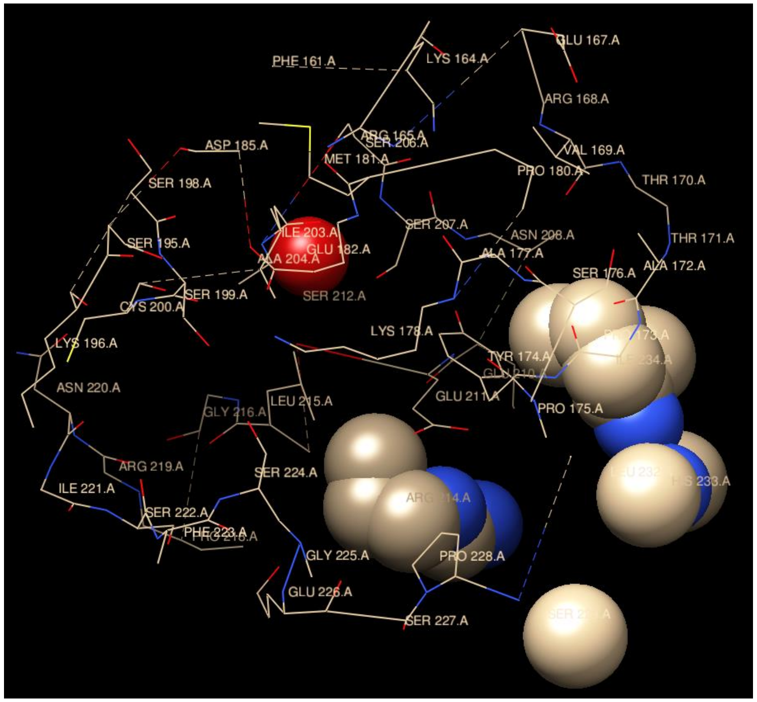

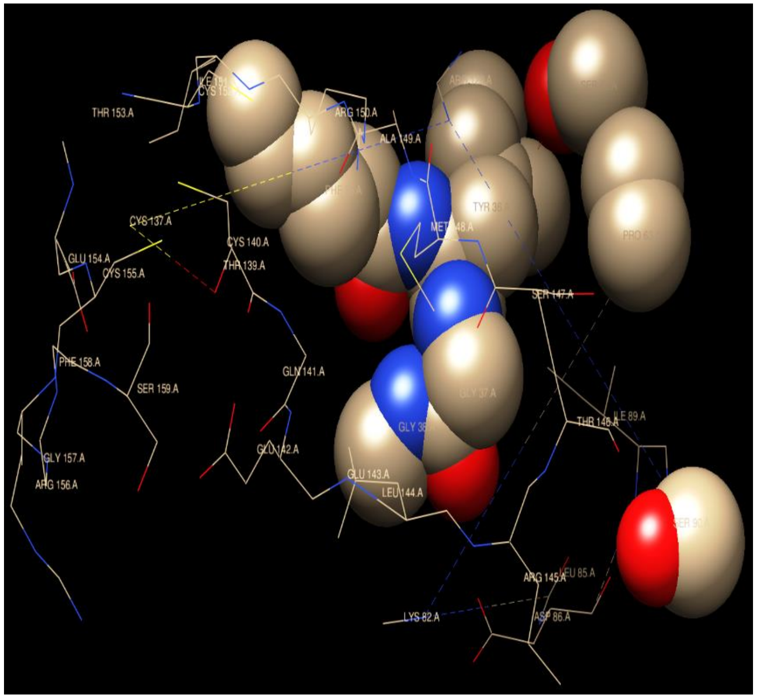

TaTIFY20 TaTIFY21

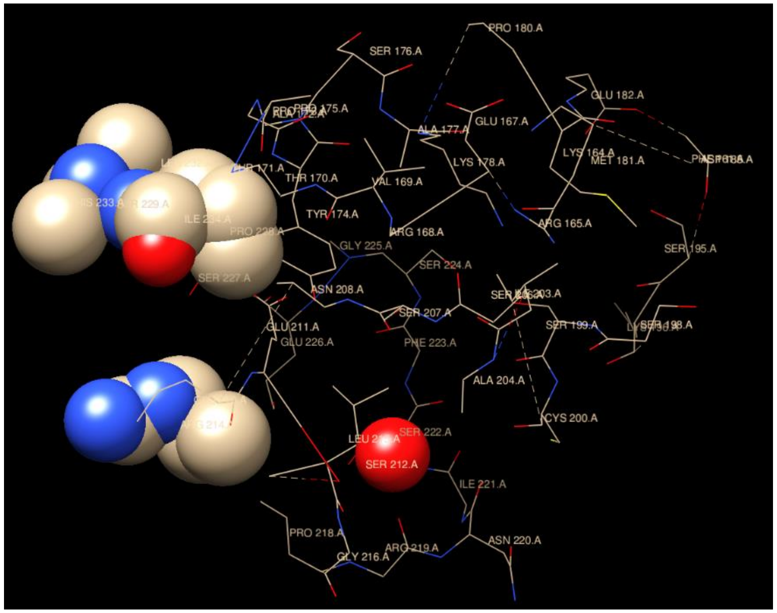

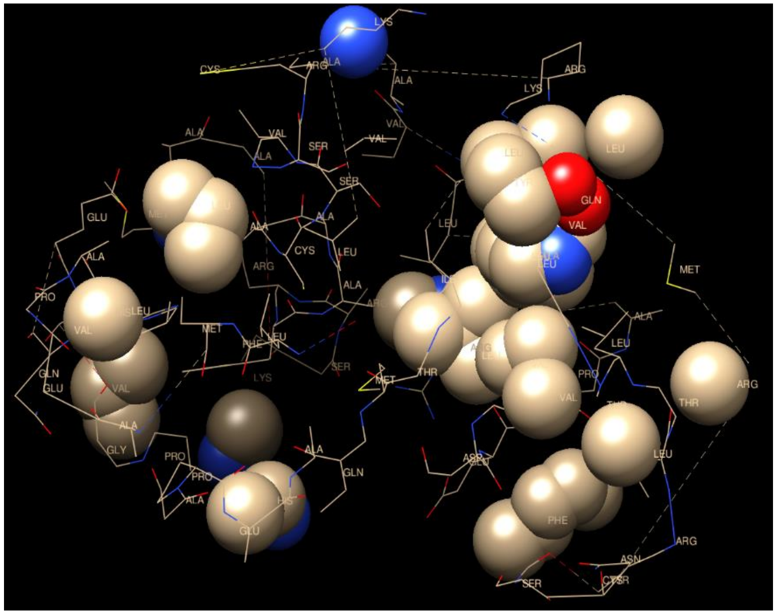

TaTIFY22 TaTIFY23

Supplementary Table S9 Statistical details of the binding sites obtained in TaTIFY TFs.

Name	Volume [Å³]	Surface [Å²]	Lipo surface [Å²]	Depth [Å]	Simple Score
TaTIFY1	2497.02	3516.88	2343.34	26.66	0.68
TaTIFY2	670.27	1232.28	792.35	19.91	0.46
TaTIFY3	1674.20	2872.35	2193.03	41.42	0.72
TaTIFY4	1238.85	1930.52	1352.14	23.59	0.71
TaTIFY5	1390.10	2736.26	2146.47	50.75	0.73
TaTIFY6	2409.34	3599.11	2521.37	32.63	0.67
TaTIFY7	1975.87	2861.27	2028.82	22.29	0.69
TaTIFY8	1677.50	2101.19	1485.52	15.46	0.69
TaTIFY9	745.09	1298.67	839.47	19.85	0.50
TaTIFY10	818.37	1505.29	1067.09	21.84	0.58
TaTIFY11	3742.58	4430.32	3211.67	25.86	0.72
TaTIFY12	1566.23	2780.81	2116.34	63.52	0.71
TaTIFY13	583.31	1102.99	717.24	14.13	0.40
TaTIFY14	745.09	1298.67	839.47	19.85	0.50
TaTIFY15	3276.74	4730.10	3320.47	23.04	0.67
TaTIFY16	1031.23	1765.85	1205.70	20.34	0.64
TaTIFY17	3239.55	4460.29	2916.04	34.64	0.66
TaTIFY18	913.70	1611.72	1222.80	26.11	0.63
TaTIFY19	165.38	517.12	352.01	15.05	0.00
TaTIFY20	1628.29	2281.49	1691.37	21.85	0.68
TaTIFY21	834.50	1509.42	969.63	15.32	0.56
TaTIFY22	1840.51	2632.67	2035.72	28.43	0.68
TaTIFY23	1891.26	2777.46	1967.02	29.82	0.64

Supplementary Figure S10. Gene Ontology categorization and distribution of *TaTIFY* genes in wheat. The results are classified under three main categories: cellular component, molecular function and biological process (x-axis). The y-axis on the left indicates the percent of genes in a category, while the y-axis on the right indicates number of genes in a specific category.

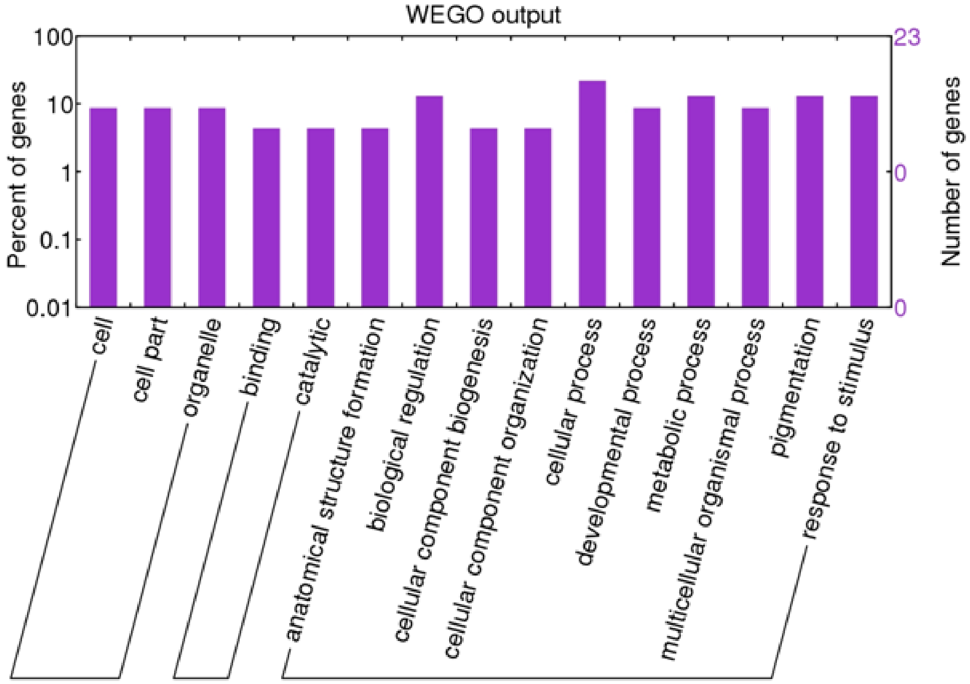

Cellular component   Molecular function   Biological process

Supplementary Table S10 Localization of novel wheat *TIFY* genes on wheat chromosome.

**Gene Name**	**Chromosome number**
TaTIFY1	2DS
TaTIFY2	2BL
TaTIFY3	4DL
TaTIFY4	4DL
TaTIFY5	4DL
TaTIFY6	2DS
TaTIFY7	5BL
TaTIFY8	5BL
TaTIFY9	5BL
TaTIFY10	2BL
TaTIFY11	4AS
TaTIFY12	4DL
TaTIFY13	2BL
TaTIFY14	2AS
TaTIFY15	7DS
TaTIFY16	2BL
TaTIFY17	5BL
TaTIFY18	2DS
TaTIFY19	2BL
TaTIFY20	2DS
TaTIFY21	2DS
TaTIFY22	5DL
TaTIFY23	2AS

Supplementary Table S11 Localization of *TaTIFY* genes on chromosomes of other monocot plants to reveal syntenic relationships.

GENE	Monocot plants used in the study					
	**Wheat**	**Sorghum**	**Rice**	**Brachypodium**	**Maize**	**Hordeum**
TaTIFY1	2,100.0%	1,94.5%	7,80.93.6%	1,91.0%	1,92.2%	2,93.6%
TaTIFY2	2,100.0%	2,94.1%	7,89.7%	1,92.2%	7,92.6%	2,89.1%
TaTIFY3	4,86.0%	1,89.7%	3,86.4%	5,90.7%	9,91.4%	4,99.1%
TaTIFY4	4,86.0%	1,93.5%	3,86.9%	1,91.7%	1,92.5%	4,94.68%
TaTIFY5	4,99.0%	1,95.3%	3,96.4%	1,90.7%	9,95.3%	4,92.0%
TaTIFY6	2,100.0%	1,94.5%	6,89.4%	1,91.0%	1,93.4%	5,88.8%
TaTIFY7	5,100.0%	2,85.0%	9,87.0%	4,85.3%	7,89.4%	2,93.6%
TaTIFY8	5,100.0%	2,98.0%	10,87.0%	4,83.75%	7,91.11%	5,88.5%
TaTIFY9	5,99.0%	2,89.2%	9,91.3%	4,98.3%	7,91.11%	5,94.0%
TaTIFY10	2,100.0%	6,87.7%	4,87.1%	5,94.06%	10,88.7%	2,94.7%
TaTIFY11	4,99.0%	1,89.7%	3,96.4%	1,94.2%	9,84.5%	4,96.1%
TaTIFY12	4,88.0%	1,87.4%	3,93.5%	1,97.2%	9,91.2%	4,99.1%
TaTIFY13	2,98.0%	6,87.7%	4,90.6%	5,92.5%	10,88.7%	2,93.2%
TaTIFY14	2,100.0%	1,94.6%	7,96.9%	1,89.8%	1,93.4%	2,94.4%
TaTIFY15	7,95.0%	1,95.8%	10,90.4%	3,93.4%	1,97.7%	7,92.3%
TaTIFY16	2,99.0%	2,94.1%	7,89.7%	1,92.3%	7,92.6%	2,89.1%
TaTIFY17	5,100.0%	2,95.8%	9,92.8%	4,98.3%	7,84.8%	5,94.0%
TaTIFY18	2,87.0%	2,100.0%	7,89.7%	1,83.9%	2,97.1%	2,86.7%
TaTIFY19	2,86.0%	6,94.4%	4,88.7%	5,90.7%	10,100.0%	2,86.5%
TaTIFY20	2,100.0%	2,93.2%	7,90.0%	1,97.1%	7,100.0%	2,84.1%
TaTIFY21	2,100.0%	2,93.2%	7,86.4%	1,88.0%	7,100.0%	2,85.0%
TaTIFY22	5,100%	7,89.8%	9,93.8%	4,87.7%	7,97.2%	5,89.9%
TaTIFY23	7,100%	1,92.8%	6,90.4%	3,89.1%	5,90.7%	7,91.9%

% implies percentage of identity in that particular plant

Supplementary Figure S11. Images showing different steps of cloning. A. PCR amplification and B. Transformats checking through PCR using gene specific and M13 forward and reverse primers. Lane 7 and 20 molecular weight markers (Top to bottom mw are: 10.000, 8000, 6000, 5000, 4000, 3000, 2500, 2000, 1500, 1000, 800, 600, 400, 200 bp), lane 1: TaTIFY3 gDNA, Lane 2: TaTIFY3 cDNA; lane 3: TaTIFY5 gDNA, Lane 4: TaTIFY5 cDNA; lane 5: TaTIFY9 gDNA, Lane 6: TaTIFY9 cDNA; lane 8: TaTIFY 19 gDNA, Lane 9: TaTIFY 19 cDNA, lane 10: TaTIFY20 gDNA, Lane 11: TaTIFY20 cDNA; lane 12: TaTIFY 23 gDNA, Lane 13: TaTIFY 23 cDNA respectively. lane 14, 15, TaTIFY3; lane 16, 17, TaTIFY5; lane 18, 19, TaTIFY19; 21, 22, TaTIFY 20; lane 23, 24, TaTIFY20; lane 25, 26, TaTIFY 23 amplified with gene specific and M13 primers respectively.

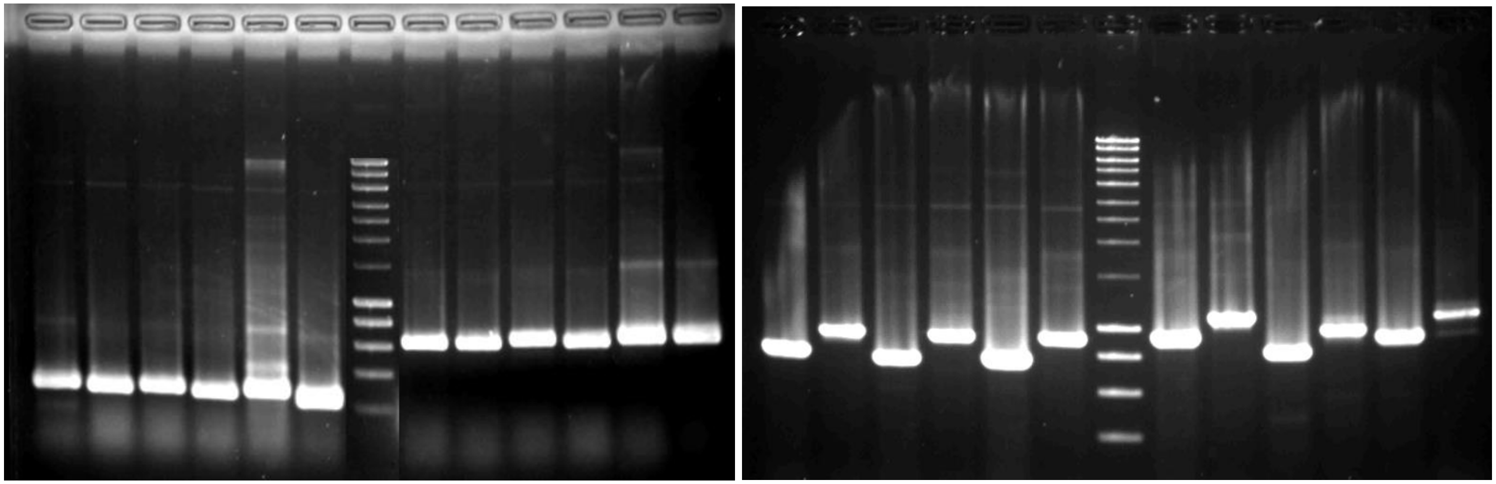

A B

Supplementary Table S12 List of primers and optimized annealing temperatures used for PCR and cloning TIFY sequences.

Family Name	Primers Sequences (5' - 3')	Annealing Temperatures cDNA	Annealing Temperatures gDNA
TaTIFY3 F	TTACCACCGGGAACAGGGTAT	50 ºC	57 ºC
TaTIFY3R	TTTTCACTATGATCTCAATCTCCCC		
TaTIFY5 F	TGCTTAGCGAAGGCCATTGA	56 ºC	52 ºC
TaTIFY5 R	TGACCAATGGTAACCCCGTG		
TaTIFY9 F	ATAAAATAATCAAGGGAACGCGC	53 ºC	53 ºC
TaTIFY9 R	GTGTTGGCCAGTTTCTCAAGG		
TaTIFY19 F	ACAGGAATACCCAGGAGCCG	53 ºC	53 ºC
TaTIFY19 R	AGTTCAGATTGGGTCCCAACTTT		
TaTIFY20 F	CCATCTCCGGTGCCAGAA	50 ºC	55 ºC
TaTIFY20 R	TCCACATCATGTCCAAAGTTCC		
TaTIFY23 F	GTCCGAACACAACACCCGA	57 ºC	57 ºC
TaTIFY23 R	AGCAGCCACACATTTGCTTG		

Supplementary Figure S12. Pairwise alignment of cloned genomic DNAs (top) and cDNAs (bottom) (A. TIFY19, B. TIFY20, C. TIFY9, D. TIFY5, E. TIFY3, F. TIFY23)

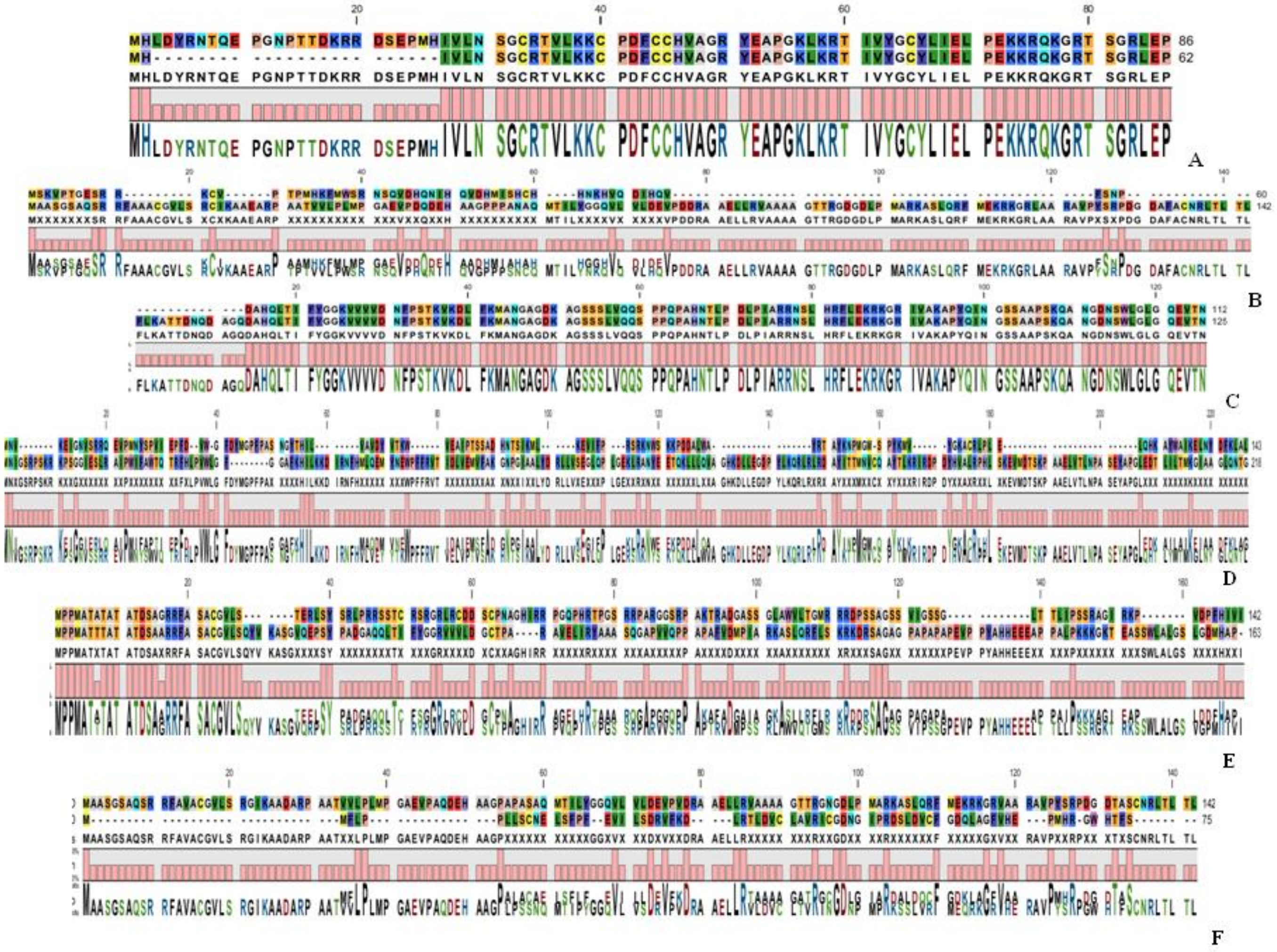

Supplementary Table S13 Physico-chemical characterization of *in silico* translated proteins from cloned *TIFY* genes.

Name of TF	No. Of amino acids	Molecular weight (Da)	Theoretical pI	Extinction coefficient	Instability Index	Aliphatic Index	GRAVY	No. of glycosylation sites	
								N-	O-
TaTIFY3	65	7470.6	11.43	1.672	77.08	70.62	-0.557	4	3
TaTIFY5	163	16859.2	9.39	0.449	76.69	69.69	-0.225	-	1
TaTIFY9	125	13414.0	9.70	0.632	46.15	72.64	-0.585	-	2
TaTIFY19	62	7049.3	9.62	0.670	50.33	78.55	-0.447	4	5
TaTIFY20	142	14856	8.98	0.209	49.09	82.75	-0.104	2	3
TaTIFY23	75	8526.9	5.03	0.674	40.35	93.47	0.237	1	5

Supplementary Table S14 Different catalytic domains present in the *in silico* translated proteins from cloned *TIFY* genes.

TRANSCRIPTION FACTORS ↓	CATALYTIC DOMAIN →															
	**ASN_GLYCOSYLATION**	**CK2_PHOSPHO_SITE**	**MYRISTYL**	**PKC_PHOSPHO_SITE**	**TIFY**	**AMIDATION**	**CAMP_PHOSPHO_SITE**	**CCT**	**TYR_PHOSPHO_SITE**	**CCT2**	**OCTAPEPTIDE**	**NLS_BP**	**FARP**	**DUF2149**	**ALA_RICH**	**CheC**	**AP NUCLEASE**
TaTIFY3	–	+	–	+	+	–	+	+	+	+	–	+	–	–	–	–	–
TaTIFY5	–	+	+	+	+	–	+	–	–	+	–	+	–	–	–	–	–
TaTIFY9	+	+	+	–	+	–	+	+	–	+	–	–	–	–	–	–	–
TaTIFY19	–	–	–	+	+	–	–	–	–	+	–	–	–	–	–	–	–
TaTIFY20	+	+	+	+	+	+	–	+	–	+	–	+	–	–	–	–	–
TaTIFY23	+	+	+	+	+	+	+	+	–	+	–	–	–	–	–	–	–

Supplementary Table S15 Prediction of Nuclear Localization Signals in translated proteins from cloned *TIFY* genes.

Name	Nuclear localization signal	
	Bipartite	Monopartite
TaTIFY3	RPSKRKPSGGIESLRAIPWIFAWTQTRFHLP	RPSKRKPSGG
TaTIFY5	RFLSKRKDRSAGAGPAPAPAPEVPPYAHHEEE	LPKKKGKTEASSW
TaTIFY9	RFLEKRKGRIVAKAPYQINGSSAAPSKQANGD	–
TaTIFY19	RYEAPGKLKRTIVYGCYLIELPEKKRQKGR	PEKKRQKGR
TaTIFY20	RVATYSFLRASLASVYSSNQQRLMEADRARRYSER	–
TaTIFY23	RRWKKERKKPKGPALGPWQCNGHPAVTHNPAGLRRP	KERKKPKGP

Supplementary Figure S13. Secondary structure of *in silico* translated proteins from cloned TaTIFY TF predicted using PSIPRED.

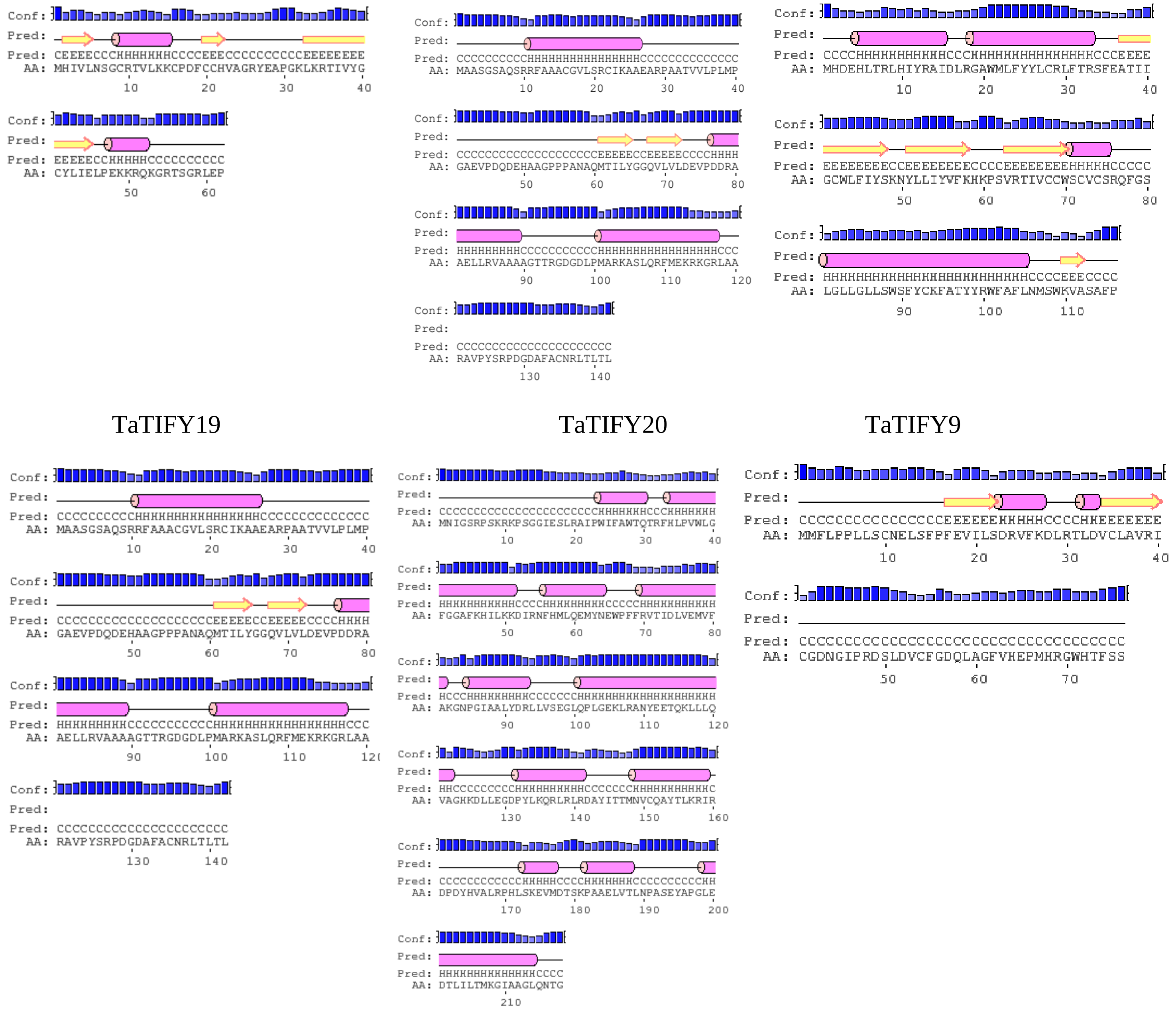

TaTIFY5 TaTIFY3 TaTIFY23

Supplementary Table S16 Secondary structure features of *in silico* translated proteins from cloned *TIFY* genes.

Name of TF	No. of α-Helices	No. of β-Strands	Extra-Cellular	Cytoplamic	Transmembrane Domain Location	Pore Lining Helix Location
TaTIFY3	3	5	N-Terminus	C-Terminus	22-37	-
TaTIFY5	6	2	C-Terminus	N-Terminus	10-25	-
TaTIFY9	4	4	C-Terminus	N-Terminus	35-57	22-37
TaTIFY19	3	2	C-terminus	N-Terminus	24-39	-
TaTIFY20	2	3	C-terminus	N-Terminus	26-45	-
TaTIFY23	4	2	N-Terminus	C-Terminus	26-41	26-41

Supplementary Figure S14. Two dimensional plots showing the disorder state of TaTIFY proteins involved in binding

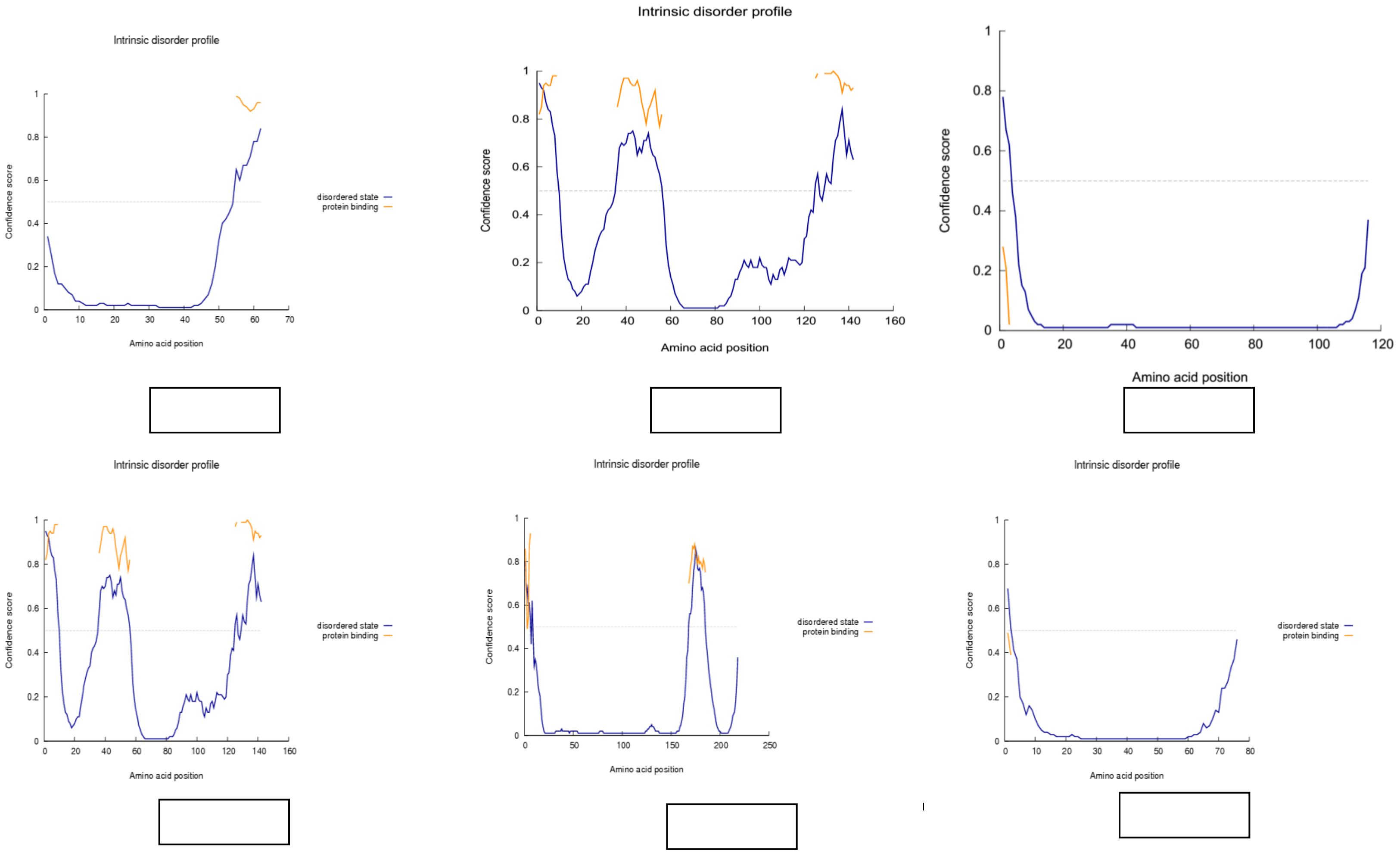

Supplementary Figure S15. Positions of pore lining helices present in two TaTIFY proteins

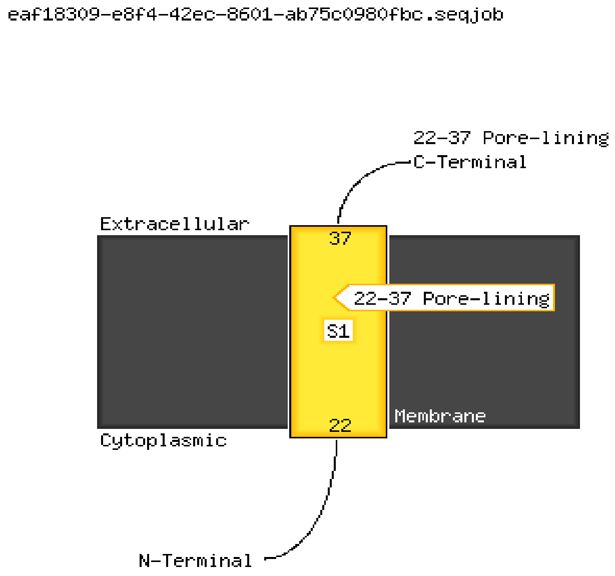

TaTIFY3

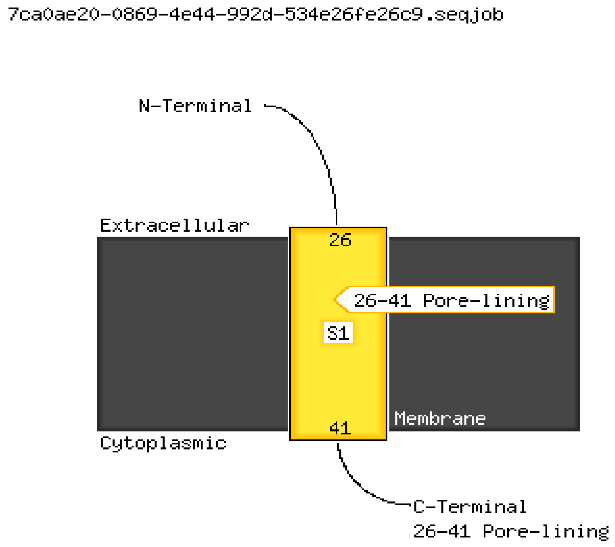

TaTIFY23

Supplementary Figure S16. Gene Ontology term enrichment analysis of cloned TaTIFY genes under biological process, molecular function and cellular component category.

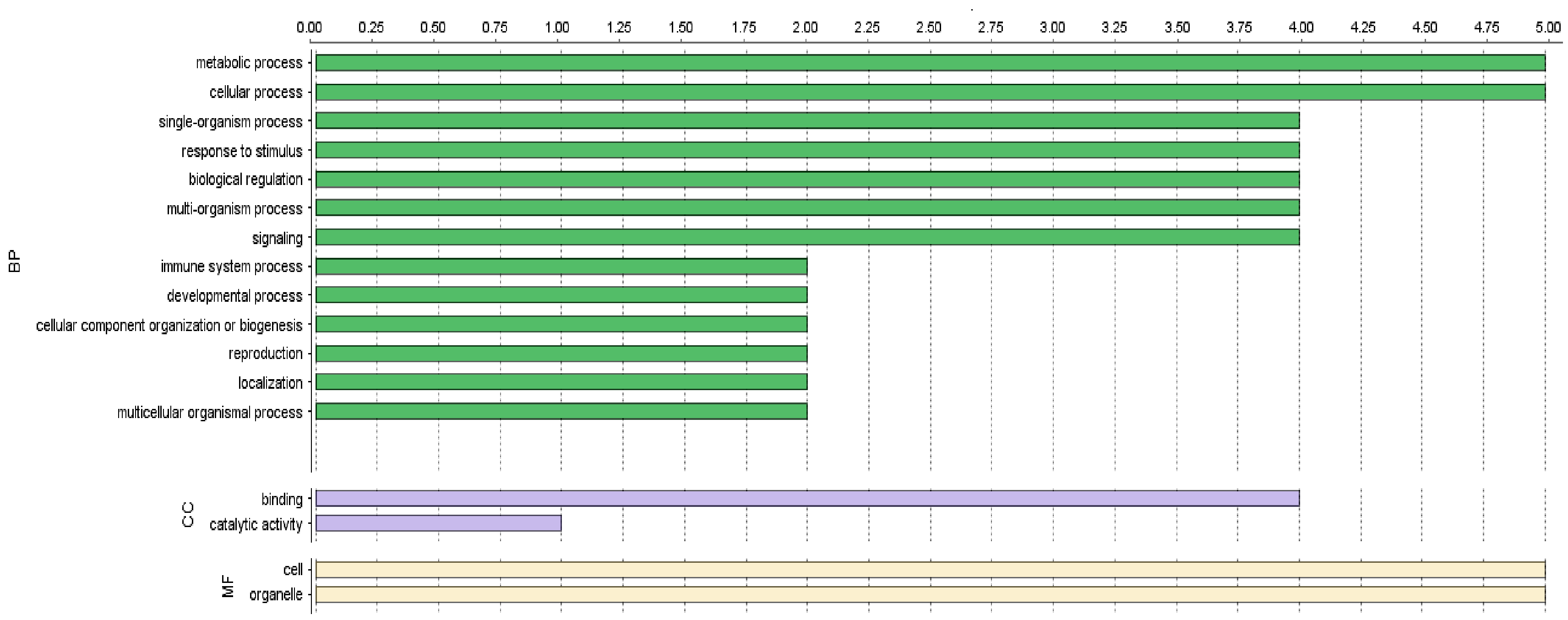

Supplementary Figure S17. Comparative synteny and expansion analysis of cloned TaZIM TF genes with sorghum, rice, maize, *Hordem* and *Brachypodium* based on orthologous and paralogous pair positions that demonstrates highly conserved synteny

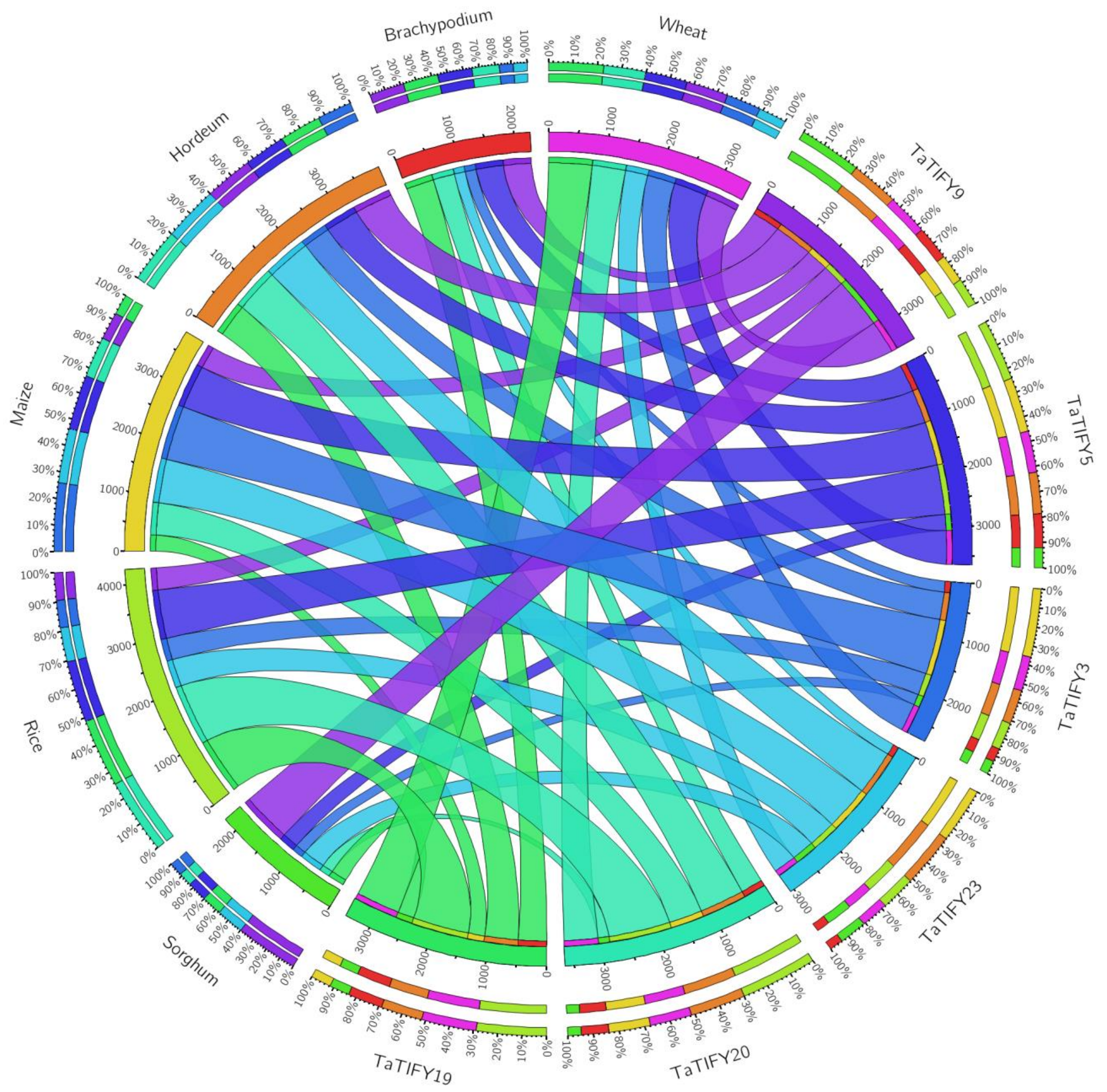

Supplementary Figure S18. Protein interaction networks of TaTIFY proteins translated from cloned genes in different monocots showing the different interactions in which they are involved

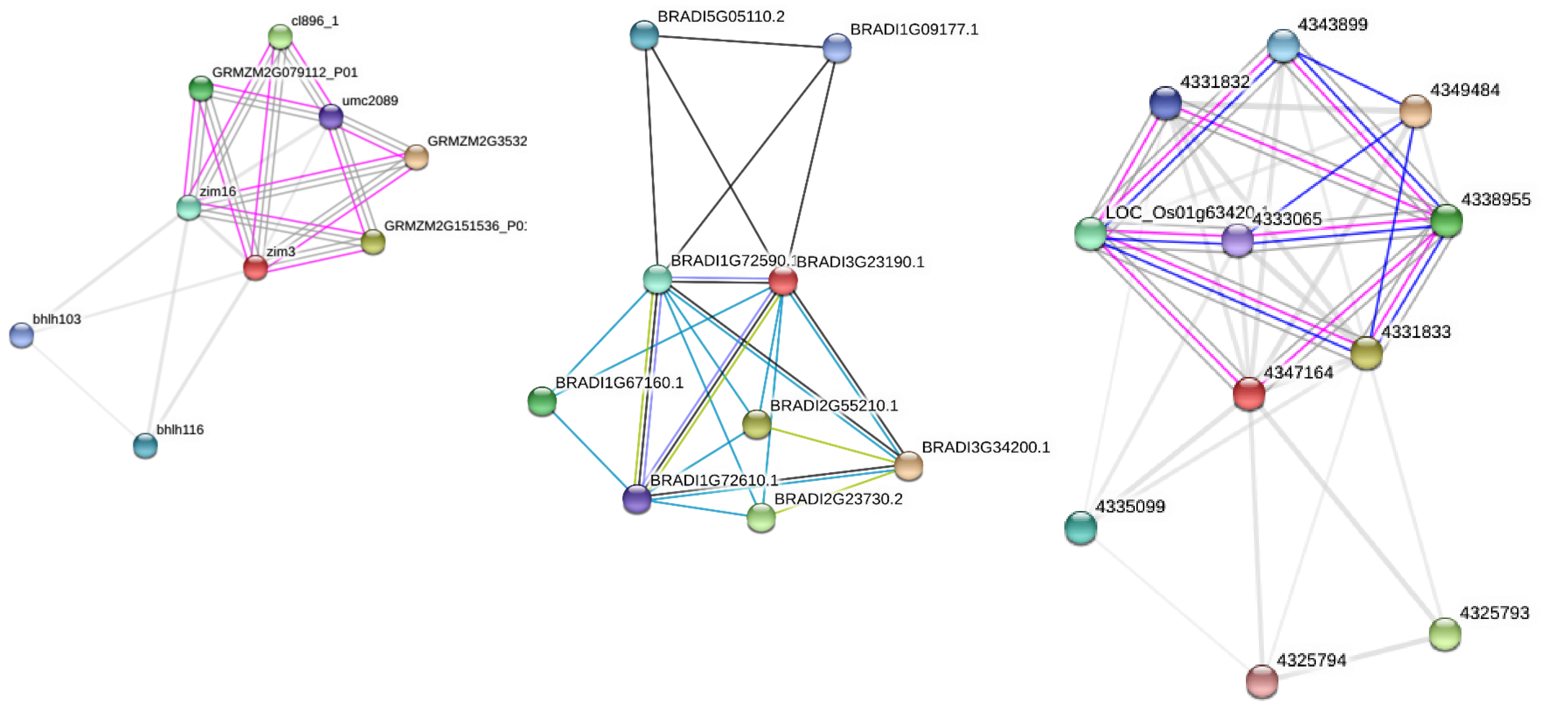

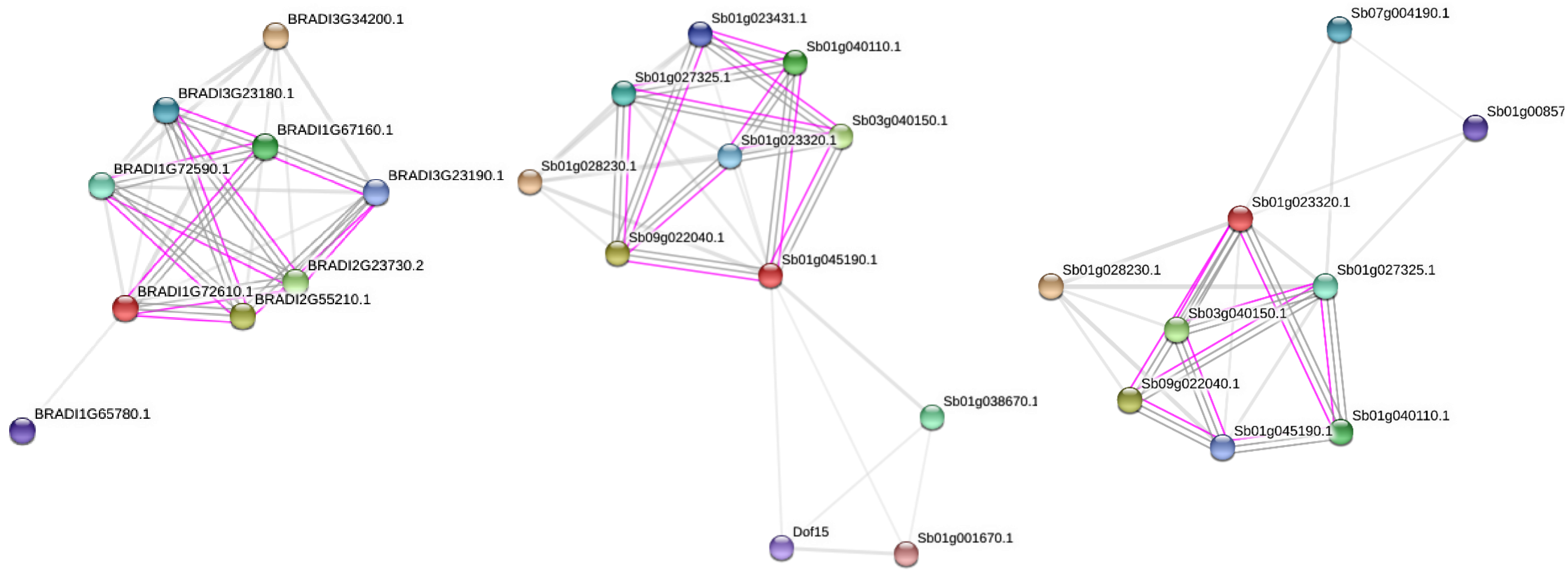
BRADI3G34200.1
BRADI3G23180.1
BRADI1G67160.1
BRADI1G72590.1
BRADI3G23190.1
BRADI2G23730.2
BRADI1G72610.1
BRADI2G55210.1
BRADI1G65780.1
Sb01g023431.1
Sb01g040110.1
Sb01g027325.1
Sb03g040150.1
Sb01g023320.1
Sb01g028230.1
Sb09g022040.1
Sb01g045190.1
Sb01g038670.1
Dof15
Sb01g001670.1
Sb07g004190.1
Sb01g00857
Sb01g023320.1
Sb01g028230.1
Sb01g027325.1
Sb03g040150.1
Sb09g022040.1
Sb01g045190.1
Sb01g040110.1

Supplementary Figure S19. Gene occurrence patterns *in silico* translated proteins from cloned genes in different plants indicating conservation of *TaTIFY* genes in Streptophyta which includes green plants comprising charophyceae (streptophyta green algae) and embryophyta (land plants).

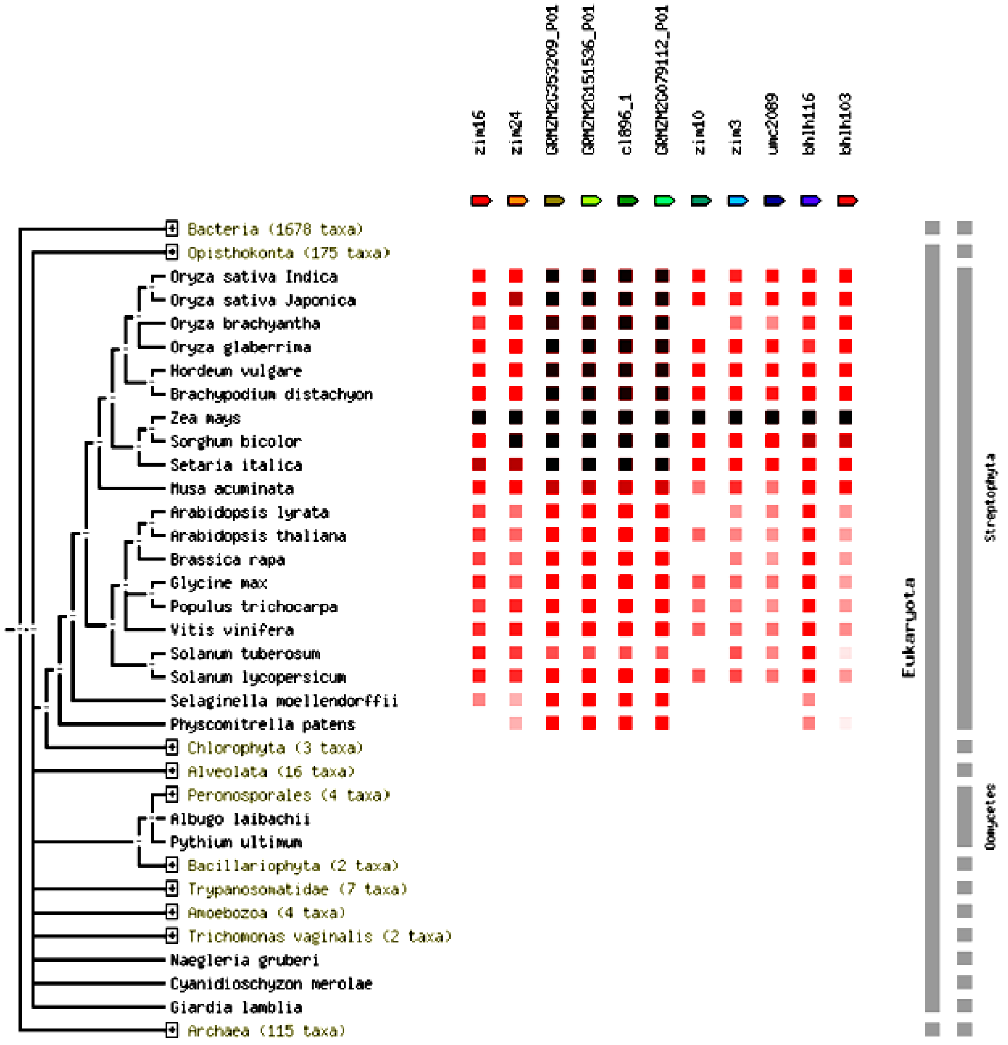

Supplementary Figure S20. Line plot showing significant difference of Wilcoxin Paired test between SM vs SPI as well as RM vs RPI of *TIFY* genes.

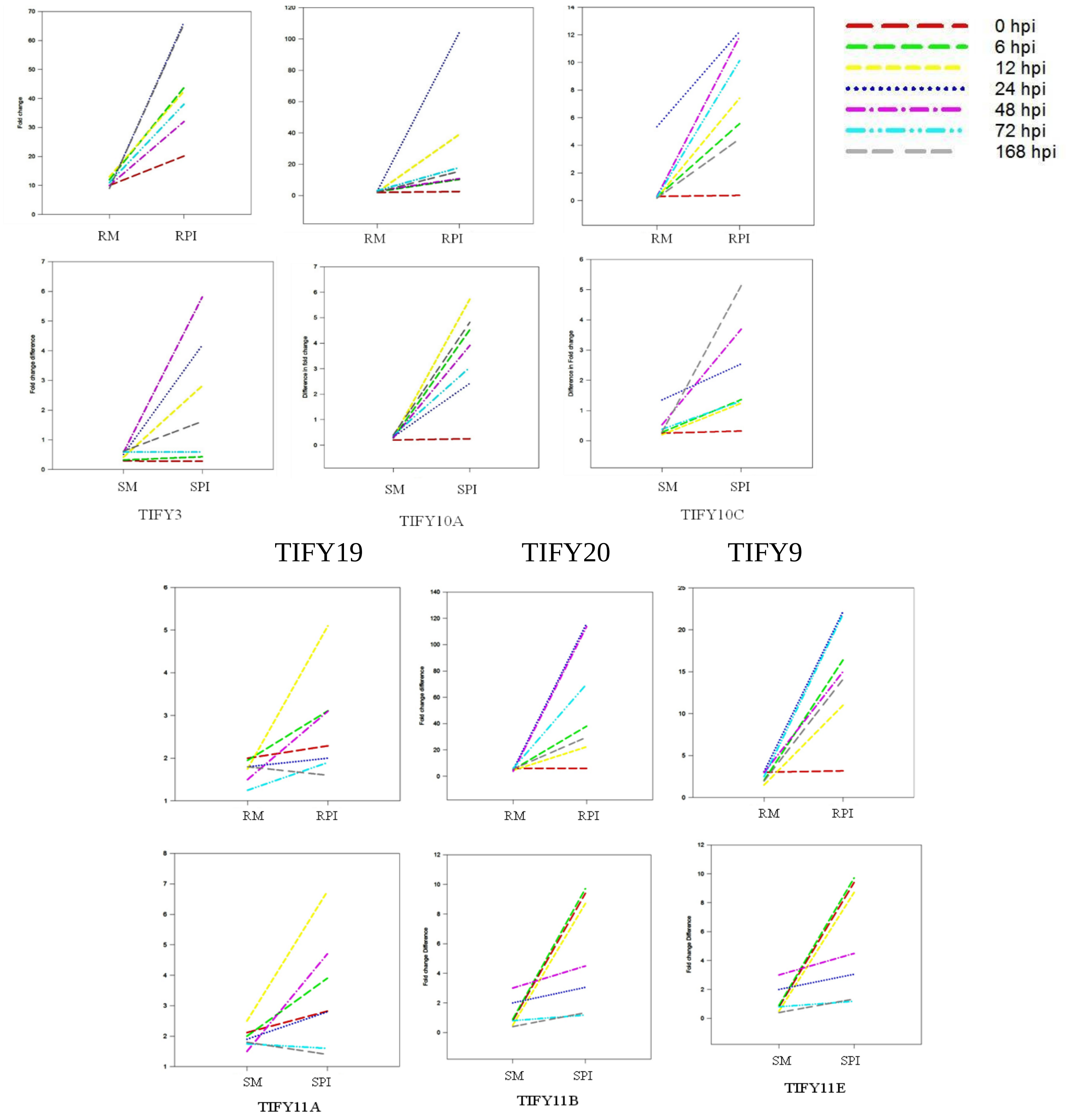

TIFY5 TIFY3 TIFY23

Supplementary Table S17 Summary of statistical analysis involving Wilcoxon signed-rank test for mock and pathogen inoculated susceptible and resistant plants with respect to *TIFY* genes.

Gene	Paired Groups	P-value	Significance
TIFY19	SM vs SPI	0.002	Yes
	RM vs RPI	0.063	Yes
TIFY20	SM vs SPI	0.030	Yes
	RM vs RPI	0.016	Yes
TIFY9	SM vs SPI	0.003	Yes
	RM vs RPI	0.001	Yes
TIFY5	SM vs SPI	0.067	Yes
	RM vs RPI	0.033	Yes
TIFY3	SM vs SPI	0.002	Yes
	RM vs RPI	0.001	Yes
TIFY23	SM vs SPI	0.002	Yes
	RM vs RPI	0.001	Yes

Supplementary Table S18A Summary of Ct values. Target (TIFY9) and GAPDH in mock and pathogen inoculated wheat NILs at selected time points.

**Time point** (HPI)	**HD2329**				**HD2329+*Lr28***			
	**Mock inoculated**		**Pathogen inoculated**		**Mock inoculated**		**Pathogen inoculated**	
	**TIFY9**	**GAPDH**	**TIFY9**	**GAPDH**	**TIFY9**	**GAPDH**	**TIFY9**	**GAPDH**
0	36.741±0.328	30.253±0.189	31.347±0.228	28.54 ±0.168	31.003±0.287	32.14±0.375	23.039±0.228	32.3±0.270
6	38.612±0.494	28.42 2±0.323	43.301±0.345	29.017 ±0.179	30.734±0.360	27.823±0.002	37.900±0.128	31.81±0.013
12	36.840±0.558	29.42± 0.066	29.352±0.583	29.307±0.137	32.082±0.363	33.14±0.242	32.706±0.907	28.726± 0.471
24	38.836±0.471	27.23±0.080	28.568±0.569	34.353 ±0.140	39.342±0.026	34.41±0.309	33.908±0.256	28.41±0.140
48	37.075±0.069	26.92±0.129	26.281±0.917	29.062±0.057	31.016±0.535	35.21±0.251	28.137±0.052	34.21±0.184
72	35.662±0.484	26.48±0.013	28.738±0.738	27.634±0.175	34.649±0.705	34.374±0.211	26.793±0.361	28.759±0.270
168	31.179±0. 230	25.25±0.114	27.051±0.055	29.360±0.371	30.356±0.919	33.68±0.295	28.532±0.077	29.162±0.297

Supplementary Table S18B Summary of Ct values. Target (TIFY19) and GAPDH genes in mock and pathogen inoculated wheat NILs at selected time points.

**Time point** (HPI)	**HD2329**				**HD2329+*Lr28***			
	**Mock inoculated**		**Pathogen inoculated**		**Mock inoculated**		**Pathogen inoculated**	
	**TIFY19**	**GAPDH**	**TIFY19**	**GAPDH**	**TIFY19**	**GAPDH**	**TIFY19**	**GAPDH**
0	35.612±0.328	30.253±0.189	33.110±0.228	28.54 ±0.168	31.003±0.287	32.14±0.375	33.485±0.228	32.3±0.270
6	32.612±0.494	28.42 2±0.323	34.393±0.345	29.017 ±0.179	30.734±0.360	27.823±0.002	29.431±0.128	31.81±0.013
12	32.840±0.558	29.42± 0.066	30.931±0.583	29.307±0.137	32.082±0.363	33.14±0.242	33.541±0.907	28.726± 0.471
24	34.836±0.471	27.23±0.080	30.257±0.569	34.353 ±0.140	39.342±0.026	34.41±0.309	32.892±0.256	28.41±0.140
48	33.075±0.069	26.92±0.129	35.138±0.917	29.062±0.057	31.016±0.535	35.21±0.251	33.952±0.052	34.21±0.184
72	35.662±0.484	26.48±0.013	32.862±0.738	27.634±0.175	34.649±0.705	34.374±0.211	43.627±0.361	28.759±0.270
168	31.179±0. 230	25.25±0.114	33.186±0.055	29.360±0.371	30.356±0.919	33.68±0.295	32.554±0.077	29.162±0.297

Supplementary Table S18C Summary of Ct values. Target (TIFY20) and GAPDH genes in mock and pathogen inoculated wheat NILs at selected time points.

**Time point** (HPI)	**HD2329**				**HD2329+*Lr28***			
	**Mock inoculated**		**Pathogen inoculated**		**Mock inoculated**		**Pathogen inoculated**	
	**TIFY20**	**GAPDH**	**TIFY20**	**GAPDH**	**TIFY20**	**GAPDH**	**TIFY20**	**GAPDH**
0	26.741±0.328	30.253±0.189	31.012±0.228	28.54 ±0.168	31.003±0.287	32.14±0.375	27.415±0.228	32.3±0.270
6	28.612±0.494	28.42 2±0.323	28.832±0.345	29.017 ±0.179	30.734±0.360	27.823±0.002	28.437±0.128	31.81±0.013
12	26.840±0.558	29.42± 0.066	28.430±0.583	29.307±0.137	32.082±0.363	33.14±0.242	28.980±0.907	28.726± 0.471
24	28.836±0.471	27.23±0.080	29.761±0.569	34.353 ±0.140	39.342±0.026	34.41±0.309	33.908±0.256	28.41±0.140
48	27.075±0.069	26.92±0.129	29.043±0.917	29.062±0.057	31.016±0.535	35.21±0.251	33.952±0.052	34.21±0.184
72	25.662±0.484	26.48±0.013	28.738±0.738	27.634±0.175	34.649±0.705	34.374±0.211	27.493±0.361	28.759±0.270
168	31.179±0. 230	25.25±0.114	27.051±0.055	29.360±0.371	30.356±0.919	33.68±0.295	26.538±0.077	29.162±0.297

Supplementary Table S18D Summary of Ct values. Target (TIFY5) and GAPDH genes in mock and pathogen inoculated wheat NILs at selected time points.

**Time point** (HPI)	**HD2329**				**HD2329+*Lr28***			
	**Mock inoculated**		**Pathogen inoculated**		**Mock inoculated**		**Pathogen inoculated**	
	**TIFY5**	**GAPDH**	**TIFY5**	**GAPDH**	**TIFY5**	**GAPDH**	**TIFY5**	**GAPDH**
0	36.741±0.328	30.253±0.189	33.199±0.228	28.54 ±0.168	31.003±0.287	32.14±0.375	38.954±0.228	32.36±0.270
6	38.612±0.494	28.42 2±0.323	39.168±0.345	29.017 ±0.179	30.734±0.360	27.823±0.002	31.550±0.128	31.81±0.013
12	36.840±0.558	29.42± 0.066	40.403±0.583	29.307±0.137	32.082±0.363	33.14±0.242	37.464±0.907	28.726± 0.471
24	38.836±0.471	27.23±0.080	38.322±0.569	34.353 ±0.140	39.342±0.026	34.41±0.309	35.478±0.256	28.41±0.140
48	37.075±0.069	26.92±0.129	37.951±0.917	29.062±0.057	31.016±0.535	35.21±0.251	38.160±0.052	34.21±0.184
72	35.662±0.484	26.48±0.013	39.093±0.738	27.634±0.175	34.649±0.705	34.374±0.211	38.860±0.361	28.759±0.270
168	31.179±0. 230	25.25±0.114	37.756±0.055	29.360±0.371	30.356±0.919	33.68±0.295	33.910±0.077	29.162±0.297

Supplementary Table S18E Summary of Ct values. Target (TIFY3) and GAPDH genes in mock and pathogen inoculated wheat NILs at selected time points.

**Time point**	**HD2329**				**HD2329+*Lr28***			
HPI	**Mock inoculated**		**Pathogen inoculated**		**Mock inoculated**		**Pathogen inoculated**	
	**TIFY3**	**GAPDH**	**TIFY3**	**GAPDH**	**TIFY3**	**GAPDH**	**TIFY3**	**GAPDH**
0	36.741±0.328	30.253±0.189	33.199±0.228	28.54 ±0.168	31.003±0.287	32.14±0.375	28.954±0.228	32.36±0.270
6	38.612±0.494	28.42 2±0.323	30.554±0.345	29.017 ±0.179	30.734±0.360	27.823±0.002	31.550±0.128	31.81±0.013
12	36.840±0.558	29.42± 0.066	32.723±0.583	29.307±0.137	32.082±0.363	33.14±0.242	27.464±0.907	28.726± 0.471
24	38.836±0.471	27.23±0.080	25.835±0.569	34.353 ±0.140	39.342±0.026	34.41±0.309	25.478±0.256	28.41±0.140
48	37.075±0.069	26.92±0.129	24.253±0.917	29.062±0.057	31.016±0.535	35.21±0.251	28.160±0.052	34.21±0.184
72	35.662±0.484	26.48±0.013	28.166±0.738	27.634±0.175	34.649±0.705	34.374±0.211	28.860±0.361	28.759±0.270
168	31.179±0. 230	25.25±0.114	24.737 ±0.055	29.360±0.371	30.356±0.919	33.68±0.295	33.910±0.077	29.162±0.297

Supplementary Table S18F Summary of Ct values. Target (TIFY23) and GAPDH genes in mock and pathogen inoculated wheat NILs at selected time points.

**Time point**	**HD2329**				**HD2329+*Lr28***			
HPI	**Mock inoculated**		**Pathogen inoculated**		**Mock inoculated**		**Pathogen inoculated**	
	**TIFY23**	**GAPDH**	**TIFY23**	**GAPDH**	**TIFY23**	**GAPDH**	**TIFY23**	**GAPDH**
0	16.741±0.328	30.253±0.189	15.802±0.228	28.54 ±0.168	31.003±0.287	32.14±0.375	30.349±0.228	32.36±0.270
6	28.612±0.494	28.42 2±0.323	29.131±0.345	29.017 ±0.179	30.734±0.360	27.823±0.002	17.019±0.128	31.81±0.013
12	26.840±0.558	29.42± 0.066	32.233±0.583	29.307±0.137	32.082±0.363	33.14±0.242	33.507±0.907	28.726± 0.471
24	28.836±0.471	27.23±0.080	31.808±0.569	34.353 ±0.140	29.342±0.026	34.41±0.309	25.376±0.256	28.41±0.140
48	27.075±0.069	26.92±0.129	30.243±0.917	29.062±0.057	31.016±0.535	35.21±0.251	34.432±0.052	34.21±0.184
72	25.662±0.484	26.48±0.013	32.126±0.738	27.634±0.175	34.649±0.705	34.374±0.211	29.844±0.361	28.759±0.270
168	21.179±0. 230	25.25±0.114	28.664±0.055	29.360±0.371	30.356±0.919	33.68±0.295	25.895±0.077	29.162±0.297

Supplementary Table S19 Spearman's Correlation of different *TIFY* genes with various phytohormone treatment.

GENE		Spearman Rank Order Correlation			Significance
		MJ	JA	SA	
TIFY19	ABA	0.42	0.42	-0.37	No
	MJ		1	0.08	No
	JA			.08	No
TIFY20	ABA	0.92	0.71	0.96	Yes
	MJ		0.67	0.85	Yes
	JA			0.82	Yes
TIFY9	ABA	0.82	0.96	0.89	Yes
	MJ		0.92	0.96	Yes
	JA			0.96	Yes
TIFY5	ABA	1	1.0	0.79	Yes
	MJ		1.0	0.79	Yes
	JA			0.79	Yes
TIFY3	ABA	0.64	0.64	0.67	Yes
	MJ		1	0.96	Yes
	JA			0.96	Yes
TIFY23	ABA	0.85	0.89	0.89	Yes
	MJ		0.96	0.96	Yes
	JA			1.0	Yes

Supplementary Table S20A Summary of Ct values. Target (TIFY19) and GAPDH genes during phytohormone treatment.

Time point	Abscisic Acid (ABA)		Methyl Jasmonate (MJ)		Jasmonic Acid (JA)		Salicylic Acid (SA)	
	**TIFY19**	**GAPDH**	**TIFY19**	**GAPDH**	**TIFY19**	**GAPDH**	**TIFY19**	**GAPDH**
0HR	31.612±0.328	29.767±0.189	29.003±0.228	29.840±0.168	31.003±0.287	29.767±0.375	31.003±0.228	30.753±0.270
1HR	32.679±0.494	32.836±0.323	33.143±0.345	28.611±0.179	32.494±0.360	27.823±0.002	30.667±0.128	31.366±0.013
2HR	30.959±0.558	29.434± 0.066	32.004±0.583	29.307±0.137	32.276±0.363	31.022±0.242	32.276±0.907	28.726± 0.471
4HR	32.370±0.471	32.86±0.080	32.645±0.569	31.888±0.140	33.342±0.026	26.153±0.309	31.585±0.256	28.079±0.140
8HR	32.194±0.069	25.718±0.129	31.681±0.917	30.192±0.057	31.352±0.535	25.505±0.251	30.999±0.052	27.575±0.184
12HR	30.833±0.484	31.300±0.013	32.075±0.738	30.965±0.175	32.338±0.705	31.366±0.211	32.212±0.361	32.014±0.270
24HR	31.609±0. 230	30.688±0.114	31.102±0.055	29.360±0.371	31.405±0.919	28.374±0.295	31.678±0.077	29.781±0.297

Supplementary Table S20B Summary of Ct values. Target (TIFY9) and GAPDH genes during phytohormone treatment.

Time point	Abscisic Acid (ABA)		Methyl Jasmonate (MJ)		Jasmonic Acid (JA)		Salicylic Acid (SA)	
	**TIFY9**	**GAPDH**	**TIFY9**	**GAPDH**	**TIFY9**	**GAPDH**	**TIFY9**	**GAPDH**
0HR	27.879±0.328	29.767±0.189	26.271±0.228	29.840±0.168	31.003±0.287	29.767±0.375	31.003±0.228	30.753±0.270
1HR	26.253±0.494	32.836±0.323	20.208±0.345	28.611±0.179	23.111±0.360	27.823±0.002	17.761±0.128	31.366±0.013
2HR	32.619±0.558	29.434± 0.066	37.227±0.583	29.307±0.137	28.154±0.363	31.022±0.242	32.030±0.907	28.726± 0.471
4HR	35.651±0.471	32.866±0.080	33.841±0.569	31.888±0.140	20.961±0.026	26.153±0.309	31.493±0.256	28.079±0.140
8HR	27.833±0.069	25.718±0.129	22.151±0.917	30.192±0.057	25.048±0.535	25.505±0.251	27.358±0.052	27.575±0.184
12HR	27.961±0.484	31.300±0.013	32.027±0.738	30.965±0.175	31.787±0.705	31.366±0.211	36.937±0.361	32.014±0.270
24HR	36.270±0. 230	30.688±0.114	33.442±0.055	29.360±0.371	32.725±0.919	28.374±0.295	35.610±0.077	29.781±0.297

Supplementary Table S20C Summary of Ct values. Target (TIFY20) and GAPDH genes during phytohormone treatment.

Time point	Abscisic Acid (ABA)		Methyl Jasmonate (MJ)		Jasmonic Acid (JA)		Salicylic Acid (SA)	
	TIFY20	GAPDH	TIFY20	GAPDH	TIFY20	GAPDH	TIFY20	GAPDH
0HR	37.879±0.328	29.767±0.189	30.003±0.228	29.840±0.168	31.003±0.287	29.767±0.375	31.003±0.228	30.753±0.270
1HR	34.137±0.494	32.836±0.323	33.692±0.345	28.611±0.179	32.195±0.360	27.823±0.002	31.173±0.128	31.366±0.013
2HR	31.495±0.558	29.434± 0.066	32.365±0.583	29.307±0.137	33.356±0.363	31.022±0.242	34.033±0.907	28.726± 0.471
4HR	31.616±0.471	32.866±0.080	30.867±0.569	31.888±0.140	30.415±0.026	26.153±0.309	31.356±0.256	28.079±0.140
8HR	32.825±0.069	25.718±0.129	33.388±0.917	30.192±0.057	31.856±0.535	25.505±0.251	30.042±0.052	27.575±0.184
12HR	30.519±0.484	31.300±0.013	31.471±0.738	30.965±0.175	33.460±0.705	31.366±0.211	33.424±0.361	32.014±0.270
24HR	32.311±0. 230	30.688±0.114	30.922±0.055	29.360±0.371	31.317±0.919	28.374±0.295	32.308±0.077	29.781±0.297

Supplementary Table S20D Summary of Ct values. Target (TIFY23) and GAPDH genes during phytohormone treatment.

Time point	Abscisic Acid (ABA)		Methyl Jasmonate (MJ)		Jasmonic Acid (JA)		Salicylic Acid (SA)	
	TIFY23	GAPDH	TIFY23	GAPDH	TIFY23	GAPDH	TIFY23	GAPDH
0HR	36.021±0.328	29.767±0.189	26.271±0.228	29.840±0.168	31.003±0.287	29.767±0.375	31.003±0.228	30.753±0.270
1HR	38.981±0.494	32.836±0.323	37.463±0.345	28.611±0.179	36.090±0.360	27.823±0.002	32.612±0.128	31.366±0.013
2HR	39.523±0.558	29.434± 0.066	39.190±0.583	29.307±0.137	40.967±0.363	31.022±0.242	39.686±0.907	28.726± 0.471
4HR	45.405±0.471	32.866±0.080	43.927±0.569	31.888±0.140	33.696±0.026	26.153±0.309	36.379±0.256	28.079±0.140
8HR	34.640±0.069	25.718±0.129	33.764±0.917	30.192±0.057	28.125±0.535	25.505±0.251	31.781±0.052	27.575±0.184
12HR	33.476±0.484	31.300±0.013	43.074±0.738	30.965±0.175	40.735±0.705	31.366±0.211	38.697±0.361	32.014±0.270
24HR	37.275±0. 230	30.688±0.114	40.659±0.055	29.360±0.371	41.433±0.919	28.374±0.295	38.800±0.077	29.781±0.297

Supplementary Table S20E Summary of Ct values. Target (TIFY3) and GAPDH genes during phytohormone treatment.

Time point	Abscisic Acid (ABA)		Methyl Jasmonate (MJ)		Jasmonic Acid (JA)		Salicylic Acid (SA)	
	TIFY3	GAPDH	TIFY3	GAPDH	TIFY3	GAPDH	TIFY3	GAPDH
0HR	35.980±0.328	29.767±0.189	26.271±0.228	29.840±0.168	31.003±0.287	29.767±0.375	31.003±0.228	30.753±0.270
1HR	31.643±0.494	32.836±0.323	28.843±0.345	28.611±0.179	32.024±0.360	27.823±0.002	25.331±0.128	31.366±0.013
2HR	29.523±0.558	29.434± 0.066	27.935±0.583	29.307±0.137	28.388±0.363	31.022±0.242	26.709±0.907	28.726± 0.471
4HR	31.049±0.471	32.866±0.080	31.774±0.569	31.888±0.140	22.118±0.026	26.153±0.309	23.362±0.256	28.079±0.140
8HR	23.048±0.069	25.718±0.129	22.905±0.917	30.192±0.057	29.399±0.535	25.505±0.251	22.200±0.052	27.575±0.184
12HR	23.567±0.484	31.300±0.013	32.822±0.738	30.965±0.175	31.421±0.705	31.366±0.211	32.795±0.361	32.014±0.270
24HR	32.069 ±0. 230	30.688±0.114	31.681±0.055	29.360±0.371	30.990±0.919	28.374±0.295	31.978±0.077	29.781±0.297

Supplementary Table S20F Summary of Ct values. Target (TIFY5) and GAPDH genes during phytohormone treatment.

	Abscisic Acid (ABA)		Methyl Jasmonate (MJ)		Jasmonic Acid (JA)		Salicylic Acid (SA)	
	TIFY5	GAPDH	TIFY5	GAPDH	TIFY5	GAPDH	TIFY5	GAPDH
0HR	35.980±0.328	29.767±0.189	26.271±0.228	29.840±0.168	31.003±0.287	29.767±0.375	31.003±0.228	30.753±0.270
1HR	38.527±0.494	32.836±0.323	37.777±0.345	28.611±0.179	39.347±0.360	27.823±0.002	35.926±0.128	31.366±0.013
2HR	39.107±0.558	29.434± 0.066	39.625±0.583	29.307±0.137	38.795±0.363	31.022±0.242	38.100±0.907	28.726± 0.471
4HR	38.290±0.471	32.866±0.080	39.417±0.569	31.888±0.140	38.711±0.026	26.153±0.309	38.562±0.256	28.079±0.140
8HR	39.446±0.069	25.718±0.129	37.008±0.917	30.192±0.057	38.701±0.535	25.505±0.251	37.226±0.052	27.575±0.184
12HR	38.614±0.484	31.300±0.013	39.374±0.738	30.965±0.175	38.155±0.705	31.366±0.211	40.408±0.361	32.014±0.270
24HR	36.084 ±0. 230	30.688±0.114	35.670±0.055	29.360±0.371	33.927±0.919	28.374±0.295	35.005±0.077	29.781±0.297

Supplementary Figure S21. A Model illustrating the role of TIFY TFs under *Puccinia triticina* induced biotic stress condition in wheat. Cellular defense signaling is triggered by recognition of pathogen derived PAMPs via distinct plasma membrane localized receptors. Mechano-sensory responses transduce MAP kinase cascades which regulate TFs that subsequently activate defense gene expression. SSV, substomatal vesicles; HMC, haustorial mother cell; G-protein, guanine nucleotide-binding proteins; PAMP, pathogen associated molecular pattern.

B. Strategies used by pathogens and pests to interfere with plant hormone biosynthesis and signaling pathways. Biotic factors secrete various effectors inside plant cells during the infection process. Once within the host cells, effectors bind to induce or repress target gene expression or protein activity. Consequently, ABA-, SA-, or Auxin-mediated defense mechanisms are activated/repressed. Abiotic factors also activate hormonal cascade and help in expression of defensive genes.

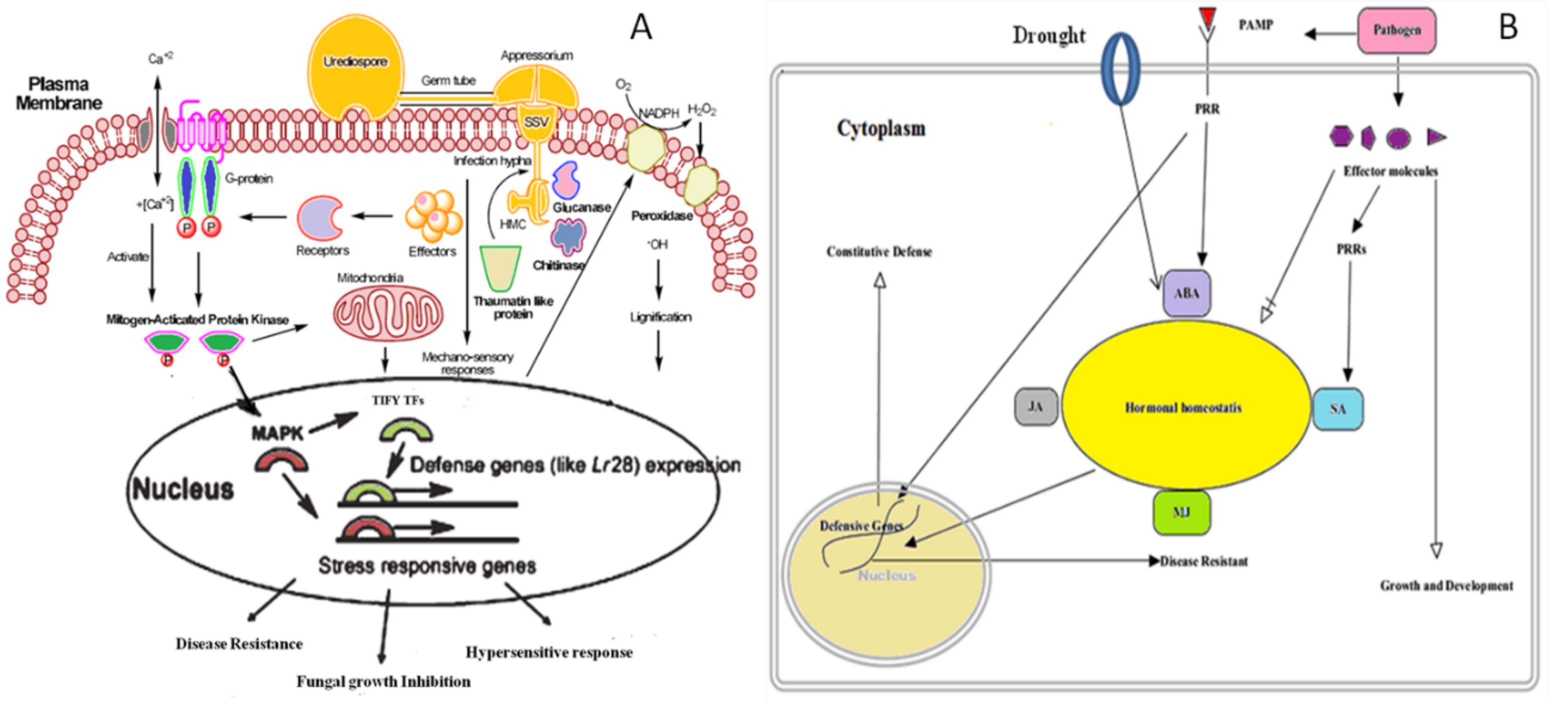

Supplementary Table S21 List of primers used for Real Time PCR studies.

Name of primer	Sequence (5' - 3')	No. of Bases
T10C F	CGAAGGCGCCCTACCAAATTA	21
T10C R	TGGAGCCTGGTCAAGTGTTCA	21
T10A F	CGTGGCGTATGCGTCTAGATT	21
T10A R	CATTCTTGCTGCTCGATCGA	20
TIFY3 F	TTTCCTTTCCACCAGCGTTTC	21
TIFY3 R	CGTTCTTCAGGCGACTCATCA	21
TIFY11A F	TTAGGCTTTAGGCCCCTCAC	20
TIFY11A R	GGTACATCGAGCTCAGTCGG	20
T11E F	TGATGTATCGCGAGCTTTGCT	21
T11E R	ACAGATACGCACGGCAAGACA	21
T11B F	AGAGAAAGCCAAGTGGTGGCA	21
T11B R	TGCTTGAAGGCACCACCAA	19
GAPDH F	AGGAAAAGATGCCTGCATTG	20
GAPDH R	CTATGTTTGCCGCGACTAGA	20
